# Recommended motor assessments based on psychometric properties in individuals with dementia: a systematic review

**DOI:** 10.1186/s11556-019-0228-z

**Published:** 2019-11-03

**Authors:** Sandra Trautwein, Philipp Maurus, Bettina Barisch-Fritz, Anela Hadzic, Alexander Woll

**Affiliations:** 1 Institute of Sports and Sports Science, Karlsruhe Institute of Technology, Engler-Bunte-Ring 15, 76131 Karlsruhe, Germany; 2 Faculty of Kinesiology, University of Calgary, Calgary, Alberta Canada

**Keywords:** Physical performance measurements, Cognitive impairment, Validity, Reliability, Frequency of use

## Abstract

**Background:**

Motor assessments are important to determine effectiveness of physical activity in individuals with dementia (IWD). However, inappropriate and non-standardised assessments without sound psychometric properties have been used. This systematic review aims to examine psychometric properties of motor assessments in IWD combined with frequency of use and effect sizes and to provide recommendations based on observed findings.

We performed a two-stage systematic literature search using Pubmed, Web of Science, Cochrane Library, ALOIS, and Scopus (inception - July/September 2018, English and German). The first search purposed to identify motor assessments used in randomised controlled trials assessing effectiveness of physical activity in IWD and to display their frequency of use and effect sizes. The second search focused on psychometric properties considering influence of severity and aetiology of dementia and cueing on test-retest reliability. Two reviewers independently extracted and analysed findings of eligible studies in a narrative synthesis.

**Results:**

Literature searches identified 46 randomised controlled trials and 21 psychometric property studies. While insufficient information was available for validity, we observed sufficient inter-rater and relative test-retest reliability but unacceptable absolute test-retest reliability for most assessments. Combining these findings with frequency of use and effect sizes, we recommend Functional Reach Test, Groningen Meander Walking Test (time), Berg Balance Scale, Performance Oriented Mobility Assessment, Timed Up & Go Test, instrumented gait analysis (spatiotemporal parameters), Sit-to-Stand assessments (repetitions> 1), and 6-min walk test. It is important to consider that severity and aetiology of dementia and cueing influenced test-retest reliability of some assessments.

**Conclusion:**

This review establishes an important foundation for future investigations. Sufficient relative reliability supports the conclusiveness of recommended assessments at group level, while unacceptable absolute reliability advices caution in assessing intra-individual changes. Moreover, influences on test-retest reliability suggest tailoring assessments and instructions to IWD and applying cueing only where it is inevitable. Considering heterogeneity of included studies and insufficient examination in various areas, these recommendations are not comprehensive. Further research, especially on validity and influences on test-retest reliability, as well as standardisation and development of tailored assessments for IWD is crucial.

This systematic review was registered in PROSPERO (CRD42018105399).

## Background

Physical activity has gained importance as therapeutic strategy for individuals with dementia (IWD), and in accordance, the number of trials investigating its effectiveness on motor and cognitive performance in IWD has increased [1]. However, methodological limitations, such as inappropriate or inconclusive motor assessments, affect the derivation of evidence. Thus, further high quality investigations are required [2–4].

Considering motor assessments, high quality is reflected by appropriateness for the intended population, sensitivity to change, sound psychometric properties, and standardisation [4–6]. In many cases, motor assessments used in previous trials failed to meet these criteria. The majority of applied assessments has predominately been developed for healthy older adults and does not consider specific characteristics of IWD [7]. However, IWD and unimpaired individuals differ in their cognitive and motor performance [8–12]. Thus, tailoring motor assessments to IWD is essential to ensure appropriateness. Furthermore, insufficient or inconsistent research regarding sensitivity to change and psychometric properties in IWD [13] restricts the derivation of meaningful conclusions from applied motor assessments [14, 15]. Referring to this, literature indicates that dementia affects reliability [6, 16–18], which was scarcely considered in previous trials. With regard to standardisation, previous research utilised a variety of motor assessments and modifications, affecting comparability [4, 13]. Therefore, inappropriateness, insensitivity, inconclusiveness, and non-standardisation limit the derivation of evidence.

Considering heterogeneous cognitive and motor impairments [10, 19], motor assessments may not be equally suitable for all IWD. Severity and aetiology of dementia, which are important determinants contributing to this heterogeneity [19, 20], potentially influence psychometric properties of motor assessments. Particularly, test-retest reliability may decrease with increasing severity of dementia, due to growing intra-individual variability or progressive difficulties to participate in motor assessments [6, 16–18]. Similarly, aetiology of dementia can influence test-retest reliability as cognitive and motor impairments vary in time of occurrence and severity in different aetiologies [14, 19]. Moreover, the influence of external cues on test-retest reliability, which are used to compensate for cognitive and motor impairments, has been discussed [16, 21].

Literature comprehensively addressing motor assessments for IWD is limited. The importance of research in this area is highlighted in a qualitative approach [22] of analysing the appropriateness of motor assessments for IWD. Additionally to elaborating recommendations, this article emphasises the need for tailoring and standardising motor assessments for IWD [22]. Moreover, three systematic reviews [7, 13, 23] and one scoping review [24] examined frequency of use, sensitivity to change, and psychometric properties. Bossers et al. [13] and McGough et al. [24] identified eight frequently applied, sensitive assessments, showing good to excellent relative test-retest reliability. Fox et al. [7] found appropriate relative test-retest reliability, but insufficient absolute test-retest reliability and limited information on validity for several motor assessments. While Lee et al. [23] determined similar intraclass correlation coefficients (ICC), they applied a more stringent rating, suggesting acceptable relative test-retest reliability only for the Berg Balance Scale (BBS). Additionally, they considered the influence of different aetiologies of dementia on relative test-retest reliability, but were not able to draw conclusions due to insufficient research. In summary, these reviews provide an important basis, but do not actually allow a comprehensive quantitative evaluation of motor assessments for IWD. Previous reviews focused on frequency of use and sensitivity to change [13, 24] or just considered relative reliability and neglected other psychometric properties such as absolute reliability or validity [13, 23, 24]. They only investigated psychometric properties of the most common motor assessments without taking into account the influences of the heterogeneity of IWD [7, 13, 24] or considering further outcomes such as frequency of use or sensitivity to change [7, 23]. Moreover, information on how psychometric properties were graded was rare [13, 23, 24], no specific recommendations were suggested [7, 23], and the results of different outcomes were not combined when drawing conclusions [7]. Finally, previous randomised controlled trials (RCT) with IWD applied additional motor assessments which were not considered in previous reviews [7, 13, 23, 24].

With respect to these limitations, we indicated the following main research gaps: (a) comprehensive quantitative approaches combining outcomes of identified reviews including psychometric properties, frequency of use, and effect sizes of motor assessments applied in previous RCT with IWD and (b) research on the influence of severity and aetiology of dementia and cueing on test-retest reliability. Therefore, the objectives of this systematic review are: (1) to quantitatively examine motor assessments for IWD used in previous RCT by comprehensively analysing psychometric properties (primary outcome), frequency of use, and effect sizes of those assessments (secondary outcomes) and (2) to assess the influence of severity and aetiology of dementia and cueing on test-retest reliability. Based on primary and secondary outcomes, this review derives recommendations, which contribute to create consensus and decrease heterogeneity of motor assessments for future research. It needs to be considered that there are several purposes and reasons for applying motor assessments. Motor assessments are essential for diagnostic purposes and to assess changes over time, e.g. in RCT. Regarding specific reasons, they are utilised to determine actual motor performance, but also to evaluate related outcomes, such as frailty [25] and risk of falls [26], or to draw conclusions on underlying cognitive performance [27]. This review focuses on motor assessments to assess changes over time, but does not further differentiate between various reasons for the use of motor assessments. Instead, it aims to provide a general overview.

## Methods

For this systematic review, we considered the guidelines and recommendations of the Preferred Reporting Items for Systematic Reviews and Meta-Analyses Statement [28, 29]. Furthermore, we registered the systematic review in PROSPERO (CRD42018105399).

We performed a two-stage literature search to address the objectives of this systematic review. A first search focused on the identification of motor assessments applied in RCT in IWD. Based on these findings, a second search (main search) aimed to determine publications examining psychometric properties of the identified motor assessments. This approach ensures to focus on those motor assessments commonly applied in IWD and allows the determination of various outcomes required for a comprehensive quantitative evaluation of motor assessments for IWD. The taxonomy of COnsensus-based Standards for the selection of health Measurement INstruments (COSMIN) initiative [30] provided the terminology and definitions of psychometric properties. In line with literature, we applied the terms relative and absolute reliability for reliability and measurement error, respectively [31]. Relative reliability, quantified by correlation coefficients, refers to the degree to which individual measurements maintain their position within a sample over repeated assessments, while absolute reliability, quantified by standard error of measurements or minimal detectable changes, is the degree to which individual measurements vary over repeated assessments [6, 31, 32].

### First search

For the first search, we examined the electronic databases Pubmed, Web of Science, Cochrane Library, and ALOIS between December 2016 and July 2018 without date restrictions. We applied terms related to dementia, physical activity, and motor performance to identify eligible trials (see Additional file 1 for complete search term), supplemented by manually checking references of indicative articles and reviews. Two reviewers independently screened titles and abstracts (ST and BB) and checked inclusion criteria during full-text analysis (ST and AH). Trials were eligible if they met the following criteria: (a) designed as (cluster) RCT, (b) included individuals with primary dementia (Alzheimer’s disease (AD), vascular dementia, frontotemporal dementia, and Lewy body disease) older than 65 years, (c) applied physical activity interventions,^1^ (d) used motor assessments independent of intended reasons, and (e) were published and written in English or German. We excluded comments, conference abstracts, protocols, and trial registrations. If there were disagreements, the two reviewers consulted a third reviewer (AW) to reach a consensual decision.

One reviewer (ST) extracted the following data from included RCT using a standardised extraction form: sample size, sample characteristics, motor assessments, means and standard deviations of baseline and post motor assessments, corresponding F/t statistics, and effect sizes. A second reviewer (AH) checked the outcomes. The two reviewers discussed ambiguities and disagreements in consensus meetings and consulted a third reviewer (BB) if they reached no agreement.

In addition to analysing frequency of use of identified motor assessments, we calculated time*group interaction effect sizes to represent their sensitivity to change. We determined Cohen’s d if F (time*group interaction) or t (between group baseline-post differences) statistics, or baseline-post differences including standard deviations were provided ([34] formulas see Additional file 2). A Cohen’s d of 0.2, 0.5, and 0.8 represents a small, medium, and large effect size, respectively [35]. Furthermore, we considered time*group interaction effect sizes provided in RCT.

This first search primarily aimed to identify motor assessments used in previous RCT with IWD and served as basis for the main search. Hence, we did not assess risk of bias.

### Main search

For the main search, we examined the electronic databases PubMed, Web of Science, Cochrane Library, and Scopus (no date restrictions) between August and September 2018 for terms related to dementia, psychometric properties, and motor assessments identified in the first search (see Additional file 3 for complete search term). Additionally, we manually checked reference lists of indicative articles. Two reviewers (ST and PM) independently screened titles and abstracts and checked inclusion criteria during full-text analysis. Trials were eligible if they fulfilled the following criteria: (a) examined psychometric properties (content validity, construct validity, criterion validity, internal consistency, intra-rater reliability, inter-rater reliability, test-retest reliability, relative and absolute reliability) of (b) motor assessments in (c) individuals with primary dementia (AD, vascular dementia, frontotemporal dementia, and Lewy body disease) aged above 65 years, (d) applied Mini-Mental State Examination (MMSE) [36], and (e) were written and published in English or German. We excluded comments and conference abstracts. The two reviewers discussed disagreements and consulted a third reviewer (BB) to resolve remaining discrepancies.

Two reviewers (ST and PM) independently extracted the following information from eligible investigations utilising a standardised data extraction form: sample size, sample characteristics, motor assessments, methodologies, and statistics of psychometric properties. Moreover, they independently assessed risk of bias of individual investigations with the COSMIN checklist [37, 38]. The two reviewers resolved disagreements through discussion and consulted a third reviewer (BB) if necessary.

Afterwards, we analysed findings of eligible investigations in a systematic narrative synthesis and summarised extracted information. In order to allow comparability of minimal detectable change values, we calculated percentage minimal detectable changes at 95% confidence interval (MDC_95%_) if any standard error of measurement or minimal detectable change was reported ([39, 40] formulas: see Additional file 4).

Moreover, we rated the results of each study against the COSMIN criteria for good measurement properties [41]. Since information on minimal important change of considered motor assessments in IWD is rare [17], and no other firm criteria for acceptable values [42] are available, we considered a MDC_95%_ higher than 30% as unacceptable [43, 44]. Based on COSMIN reliability criteria for good measurement properties [41] and indications for unacceptable values [43, 44], we rated relative and absolute reliability as follows:

- sufficient relative/absolute reliability (+): ICC ≥ 0.70/minimal detectable change at 95% confidence interval < minimal important change
- indeterminate relative/absolute reliability (?): ICC not reported/minimal important change not defined
- insufficient relative/absolute reliability (−): ICC < 0.70/minimal detectable change at 95% confidence interval > minimal important change
- unacceptable absolute reliability (↓): MDC_95%_ > 30%

Subsequently, we summarised overall evidence and graded quality of evidence using the Grading of Recommendations Assessment, Development, and Evaluation approach, which considers risk of bias, inconsistency, imprecision, and indirectness of included investigations [41, 45]. Additionally, we analysed the influence of severity and aetiology of dementia and cueing on test-retest reliability. Therefore, we determined severity of dementia according to reported MMSE values (mild: MMSE = 26–17, moderate: MMSE = 17–10, severe: MMSE< 10 [46–48]) and/or classification of publications if range of MMSE was not reported. Due to insufficient information on aetiology, we were only able to compare between AD and various or not reported types. In accordance with Muir-Hunter et al. [14] we defined cueing as “providing any additional verbal, visual, or tactile direction necessary to ensure correct performance of the task after the initial set of standardized instructions was given”. To investigate its influence on test-retest reliability, we classified cueing in five categories, considering information in identified psychometric property studies: (a) not reported, (b) no cueing, (c) verbal cueing, (d) verbal and visual/tactile cueing, and (e) more extensive cueing than (c) and (d) including physical assistance.

## Results

### Systematic searches (first and main search)

**Fig. 1:**
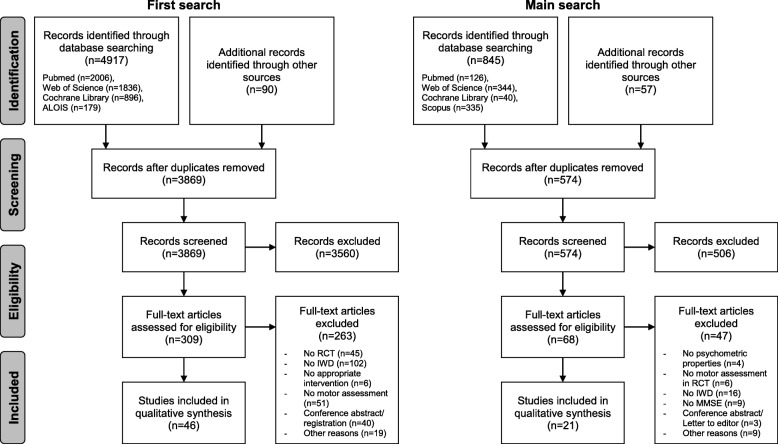
Flow of information (IWD: individuals with dementia, MMSE: Mini-Mental State Examination, n: number, RCT: randomised controlled trial)

The first search revealed 5007 publications. After removing duplicates and initial screening on titles and abstracts, we screened the full texts of 309 publications and included 46 RCT for further analysis. For the main search, we obtained 902 publications. Removing duplicates and initial screening on titles and abstracts yielded 68 publications, of which we scanned full texts. Eventually, we included 21 eligible investigations in the narrative data synthesis (see Fig. 1, further information on study characteristics and data extractions are provided in Additional files 5, 6, 7 and 8).

### Motor assessments applied in previous randomised controlled trials

Previous RCT with IWD utilised 57 different motor assessments to determine balance, mobility and gait, strength, endurance, flexibility, and functional performance. Psychometric properties of 28 of these assessments were investigated in IWD. Table 1 contains a short description of all identified motor assessments with available psychometric property studies (see Additional file 9 for motor assessments identified during first search without available information on psychometric properties).

**Table 1:** Description, frequency of use, and effect sizes of motor assessments applied in previous randomised controlled trials

Motor assessment	Description	Frequency of use	Time*group interaction effect size
*Balance*			
FICSIT-4 [49]	*Task*: performing four different stances with eyes open for 10 seconds: (a) feet together, (b) semi-tandem, (c) tandem, (d) single-leg *Measurement*: score [0–5], which rates performance according to ability to maintain stances	1 RCT (*n* = 109) [50]	–
Modified Clinical Test of Sensory Interaction of Balance [51]	*Task*: standing on a platform (NeuroCom Balance Master) as quietly as possible for 10 seconds under four sensory conditions: eyes open and closed standing on firm surface and foam *Measurements*: sway velocity [deg/s], composite score for all conditions	1 RCT (*n* = 40) [52]	–
Limits of Stability [51]	*Task*: standing on NeuroCom Balance Master and moving cursor from centre box directly to eight target boxes as fast and as close as possible by shifting weight *Measurements*: reaction time [s], movement velocity [deg/s], maximum excursion [%], directional control [%], summary composite score	1 RCT (n = 40) [52]	–
Physiomat-Trail-Making Task [53]	*Task*: standing on Physiomat and connecting digits by shifting weight *Measurements*: total duration [s], accuracy of sway path [digits/ms]	1 RCT (*n* = 84) [54]	–
Physiomat-Follow-The-Ball Task [53]	*Task*: standing on Physiomat and moving cursor from centre of screen directly to targets as fast as possible by shifting weight *Measurements*: total duration [s], accuracy of sway path [digits/ms]	1 RCT (n = 84) [54]	–
FR [55]	*Task*: standing next to a wall, holding one arm parallel to a metre stick attached to the wall at shoulder height, and reaching forward as far as possible without losing balance or changing foot position *Measurement*: distance from starting to end position [cm]	5 RCT (*n* = 204) [52, 56–59]	Small to large ^c^
Hill Step Test [60]	*Task*: stepping 1 foot onto a block and returning it to the floor as quickly as possible for 15 seconds *Measurement*: number of repetitions	2 RCT (*n* = 54) [52, 61]	–
Step Quick Turn Test [51]	*Task*: taking two steps forward on NeuroCom Balance Master, quickly turning, and returning to starting point *Measurements*: turn time [s], turn sway [deg/s]	1 RCT (n = 40) [52]	–
Figure of Eight Test [62]	*Task*: walking a lap of a standard figure-eight trajectory as quickly and accurately as possible *Measurements*: walking speed [m/s], number of oversteps	1 RCT (n = 109) [50]	–
GMWT [63]	*Task*: walking over a meandering curved line as quickly and accurately as possible *Measurements*: walking speed [m/s], number of oversteps	1 RCT (*n* = 109) [50]	–
BBS [64]	*Task*: 14-item functional balance assessment with simple everyday tasks (reaching, bending, transferring, standing, and rising), which are graded on a five-point ordinal scale (0 to 4) *Measurement*: score [0–56]	11 RCT (*n* = 648) [56, 57, 65–73]	Small to large ^c/r^
Modified BBS [64]	*Task*: abbreviated version of the original 14-item BBS, excluding three items (chair-to-chair transfer, forward reach with outstretched arm, and alternate stepping on-off stool) *Measurement*: score [0–44]	1 RCT (*n* = 23) [74]	–
POMA [75]	*Task*: scale with two parts, assessing balance (B) and gait (G) (B) sitting balance, rising from a chair and sitting down, standing balance (with eyes open and closed), and turning balance (G) gait initiation, step length and height, symmetry, continuity, path direction, and trunk sway *Measurements*: total score [0–28], balance score [0–16], gait score [0–12]	7 RCT (*n* = 300) [68, 70, 76–80]	No to large ^c/r^
*Mobility and gait*			
TUG [81]	*Task*: standing up from a chair, walking three metres, turning around, walking back to chair, and sitting down *Measurements*: time [s], number of steps	16 RCT (*n* = 1001) [50, 52, 56, 58, 59, 66, 68, 70, 73, 78, 79, 82–86]	No to large ^c/r^
Cognitive TUG [87]	*Task*: TUG with additional cognitive task (counting backwards by threes/evoke names of animals) *Measurement*: time [s]	2 RCT (*n* = 60) [52, 56]	–
Manual TUG [25, 87]	*Task*: TUG with additional manual task (carrying a glass of water) *Measurement*: time [s]	1 RCT (n = 40) [52]	–
6 m WT [88]	*Task*: walking six metres with comfortable pace *Measurements*: walking speed [m/s], step length [m]	3 RCT (*n* = 379) [50, 71, 89]	–
4 m WT [88]	*Task*: walking four metres with comfortable pace *Measurement*: walking speed [m/s]	2 RCT (*n* = 244) [90, 91]	Small ^c/r^
Instrumented gait analysis [92]	*Task*: walking with comfortable/fast pace over an electronic walkway (GAITRite, Bessou locometer, NeuroCom Balance Master) *Measurements*: walking speed [cm/s, m/s], cadence [steps/min], stride/step length [cm, m], stride time [s], double/single support [% of stride time], double limb support time [s], step width [cm], step time variability [CV], Walk-Ratio [step length/cadence]	6 RCT (*n* = 370) [52, 78, 93–96]	Small to large ^c/r^
*Strength*			
5x STS [97]	*Task*: performing five repetitions of the STS task without upper extremity assistance *Measurement*: time [s]	7 RCT (*n* = 358) [52, 58, 68, 77, 78, 94, 98]	No to large ^c/r^
STS on NeuroCom Balance Master [51]	*Task*: standing up from a seated position without upper extremity assistance *Measurements*: rising index [% of body weight], centre of gravity sway velocity [deg/s]	1 RCT (*n* = 40) [52]	–
ACSID [99]	*Task*: performing five repetitions of the STS task without upper extremity assistance while motor and cognitive aspects of movement process are qualitatively rated *Measurements*: total score [0–10], sub scores ‘recall and initiation’ [0–5], ‘effective performance’ [0–5]	1 RCT (*n* = 77) [100]	Large ^c/r^
30s CST [17, 101]	*Task*: performing as many repetitions of STS task as possible in 30 s *Modified version*: use of upper extremity assistance is allowed *Measurement*: number of repetitions	5 RCT (*n* = 408) [56, 71, 74, 80, 84] Modified: 1 RCT (*n* = 109) [50]	Large ^c/r^
Handgrip dynamometer [102]	*Task*: putting maximum force on a dynamometer *Measurement*: maximum handgrip strength [KPa, kg]	3 RCT (*n* = 263) [67, 78, 94]	No ^r^
Maximum isometric strength assessed with dynamometers [103]	*Task*: pushing as hard as possible against a dynamometer after adopting a standardised position *Measurements*: maximum strength [N] and integral over time [Ns] for knee extension, knee flexion, and ankle flexion	2 RCT (*n* = 216) [50, 78]	–
*Endurance*			
6 min WT [104]	*Task*: walking for 6 minutes with comfortable pace *Measurement*: distance [m, ft]	5 RCT (*n* = 359) [50, 57, 105–107]	–
*Functional performance*			
SPPB [108]	*Task*: three subtests including standing balance (tandem, semi-tandem, and side-by-side stands), walking speed over an 8-ft walking course, and 5x STS *Measurement*: score [0–12]	3 RCT (*n* = 313) [77, 90, 109]	Small to medium ^c/r^
E-ADL Test [110, 111]	*Task*: five items (pouring a drink, spreading butter on a sandwich and cutting the sandwich, open a small cupboard with a key, washing and drying hands, and tying a bow on a small wrapped present), which are rated according to correctly performed substeps (0–6 points) *Measurement*: score [0–30]	2 RCT (*n* = 192) [112, 113]	–

*4 m WT* 4-m walk test, *5x STS* Five Times Sit-to-Stand Test, *6 m WT* 6-m walk test, *6 min WT* 6-min walk test, *30s CST* 30-s chair stand test, *ACSID* Assessment of Compensatory Sit-to-Stand Maneuvers in People With Dementia, *BBS* Berg Balance Scale, *E-ADL Test* Erlangen Test of Activities of Daily Living, *FICSIT-4* Frailty and Injuries: Cooperative Studies of Intervention Techniques - subtest 4, *FR* Functional Reach Test, *GMWT* Groningen Meander Walking Test, *n* Number of analysed participants, *POMA* Performance Oriented Mobility Assessment, *RCT* Randomised controlled trial/s, *SPPB* Short Physical Performance Battery, *STS* Sit-to-Stand, *TUG* Timed Up & Go Test.

^c^ calculated effect size, ^r^ effect size provided of randomised controlled trial

### Psychometric properties

Seventeen of twenty-one studies examining psychometric properties focused on inter-rater and/or test-retest reliability. Herein, they determined consistency among different evaluators simultaneously rating the same participant, and between repeated measurements, respectively [32]. Investigations assessing content, construct, and criterion validity, internal consistency, and intra-rater reliability were rare. Thus, we only summarised results and did not derive conclusions.

#### Summary for content, construct, and criterion validity, internal consistency, and intra-rater reliability^2^

The systematic search did not identify any investigation examining content validity. Based on hypotheses testing or revealing known group differences, construct validity was suggested for Physiomat assessments, the Erlangen Test of Activities of Daily Living (E-ADL Test), and knee extensor strength assessed with dynamometers [53, 110, 111, 114]. Seven investigations include information on criterion validity (concurrent and predictive validity), correlation with, or prediction of external criteria. For the E-ADL Test, criterion related validity was determined based on the relation between achieved scores and level of care [111]. Concurrent validity with spatiotemporal gait parameters or 2D-video motion analysis was established for a modified BBS, Short Physical Performance Battery (SPPB), and Assessment of Compensatory Sit-to-Stand Maneuvers in People With Dementia (ACSID) [26, 99]. Moreover, both the SPPB and 6-min walk test (6 min WT) significantly correlated with peak oxygen consumption (assessed with a cycle ergometer test), suggesting that these assessments are useful in identifying individuals with low aerobic capacity [115]. Furthermore, knee extensor strength was found to be a significant predictor for several activities of daily living, gait, and sit-to-stand (STS) performance [114, 116]. No predictive validity concerning future falls could be observed for Timed Up & Go Test (TUG), Performance Oriented Mobility Assessment (POMA), and Five Times Sit-to-Stand Test (5x STS) [117].

Considering internal consistency, three studies observed Cronbach’s α between 0.37 and 0.77 for E-ADL Test [110, 111] and 0.95 for BBS [15]. Furthermore, one study examining ACSID total score determined intra-rater reliability based on ICC ranging between 0.72 and 0.90 [99].

#### Inter-rater reliability (relative and absolute reliability)

Five studies assessed inter-rater reliability of nine assessments. ICC ranged from 0.72 to 1.00 and MDC_95_ included values between 0.0 and 98.0% [14, 15, 43, 99, 118]. Accordingly, all assessments reached sufficient relative inter-rater reliability. Quality of evidence for relative inter-rater reliability was high for BBS, moderate for TUG, and low or very low for all other assessments. Grading MDC_95%_, TUG and 6-m walk test (6 m WT) showed sufficient absolute inter-rater reliability, while it was insufficient/unacceptable for 4-m walk test (4 m WT), and indeterminate for all other assessments. Quality of evidence for absolute inter-rater reliability was low for 6 m WT and 30-s chair stand test (30s CST), and moderate for all remaining assessments (see Table 2).

**Table 2:** Relative and absolute inter-rater reliability

	Variable	Study	Relative inter-rater reliability							Absolute inter-rater reliability						
			ICC	Rating	Risk of bias	Inconsistency	Imprecision	Indirectness	Quality of evidence	MDC_95%_	Rating	Risk of bias	Inconsistency	Imprecision	Indirectness	Quality of evidence
Balance																
FR	Distance	1 study of adequate quality (*n* = 15) [14]	0.79	+	Serious	No	*n* < 50	No	Very low	Not assessed						
GMWT	Time	1 study of adequate/very good quality (*n* = 53) [43]	0.99	+	Serious	No	*n* = 50–100	No	Low	14.5%	?	No	No	n = 50–100	No	Moderate
	Number of oversteps	1 study of adequate/very good quality (n = 53) [43]	0.99	+	Serious	No	n = 50–100	No	Low	17.1%	?	No	No	n = 50–100	No	Moderate
BBS	Score	3/2 studies of adequate/very good quality (*n* = 101/86) [14, 15, 43]	0.72–0.99	+	No	No	No	No	High	5.9–7.1%	?	No	No	n = 50–100	No	Moderate
Mobility and gait																
TUG	Time	2 studies/1 study of adequate/very good quality (*n* = 68/53) [14, 43]	0.98–0.99	+	No	No	n = 50–100	No	Moderate	7.9%	+^b^	No	No	n = 50–100	No	Moderate
6 m WT	Walking speed	1 study of adequate/very good quality (*n* = 33) [15]	0.97	+	Serious	No	n < 50	No	Very low	15.7%	+^c^	No	No	n < 50	No	Low
4 m WT	Time	1 study of adequate/very good quality (n = 53) [43]	0.82	+	Serious	No	n = 50–100	No	Low	98.0%	-^c^/↓	No	No	n = 50–100	No	Moderate
Strength																
ACSID	Score	1 study of very good quality (*n* = 94) [99]	0.85	+	No	No	n = 50–100	No	Moderate	Not assessed						
30s CST	Repetitions	1 study of adequate/very good quality (n = 33) [15]	1.00	+	Serious	No	n < 50	No	Very low	0.0%	?	No	No	n < 50	No	Low
Endurance																
6 min WT	Distance	1 study of adequate quality (n = 33)^a^ [118]	0.97–0.99	+	Serious	No	n < 50	No	Very low	Not assessed						
	Walking speed	1 study of adequate quality (n = 33)^a^ [118]	0.96–0.98	+	Serious	No	n < 50	No	Very low	Not assessed						

*4 m WT* 4-m walk test, *6 m WT* 6-m walk test, *6 min WT* 6-min walk test, *30s CST* 30-s chair stand test, *ACSID* Assessment of Compensatory Sit-to-Stand Maneuvers in People With Dementia, *BBS* Berg Balance Scale, *FR* Functional Reach Test, *GMWT* Groningen Meander Walking Test, *ICC* Intraclass correlation coefficient, *MDC*_*95%*_ Percentage minimal detectable changes at 95% confidence interval, *n* Total number of participants, *TUG* Timed Up & Go Test.

Rating according to COSMIN criteria for good measurement properties: + = sufficient, − = insufficient, ? = indeterminate, ↓ = unacceptable absolute inter-rater reliability.

^a^ inter-rater reliability was determined on 2 times of measurement, ^b^ minimal important change (TUG) = 10.1 s [17, 119], ^c^ minimal important change (walking speed) = 0.21 m/s [17, 119]

Regarding balance assessments, ICC were higher for Groningen Meander Walking Test (GMWT) and BBS than for Functional Reach Test (FR). Furthermore, MDC_95%_ were lower for BBS compared to GMWT. Focusing on GMWT, time measurement showed lower MDC_95%_ than number of oversteps. For mobility and gait, ICC increased and MDC_95%_ decreased from 4 m WT, through 6 m WT, to TUG. Considering strength assessments, ICC were higher for 30s CST counting repetitions than for ACSID rating STS performance, while MDC_95%_ was only determined for 30s CST. Since ICC was only assessed for 6 min WT, a comparison of inter-rater reliability of endurance assessments was not possible (see Table 2).

#### Test-retest reliability (relative and absolute reliability)

Fifteen studies investigated test-retest reliability considering 24 assessments. ICC ranged between 0.02 and 0.99 and MDC_95%_ varied from 6.8 to 225.7% [5, 6, 14, 17, 26, 43, 51, 53, 63, 102, 110, 114, 118, 120, 121] (see Table 3).

**Table 3:** Relative and absolute test-retest reliability

	Variable	Study	Relative test-retest reliability							Absolute test-retest reliability						
			ICC	Rating	Risk of bias	Inconsistency	Imprecision	Indirectness	Quality of evidence	MDC_95%_	Rating	Risk of bias	Inconsistency	Imprecision	Indirectness	Quality of evidence
Balance																
FICSIT-4	Score	1 study of adequate quality (*n* = 58)^a^ [17]	0.79–0.82	+	Serious	No	n = 50–100	No	Low	58.9–71.1%	↓	Serious	No	n = 50–100	No	Low
Modified Clinical Test of Sensory Interaction of Balance	Sway velocity	1 study of adequate quality (*n* = 14) [51]	0.91	+	Serious	No	n < 50	No	Very low	36.5%	↓	Serious	No	*n* < 50	No	Very low
Limits of Stability	Reaction time	1 study of adequate quality (*n* = 14) [51]	0.52	–	Serious	No	n < 50	No	Very low	38.0%	↓	Serious	No	n < 50	No	Very low
	Movement velocity	1 study of adequate quality (n = 14) [51]	0.48	–	Serious	No	n < 50	No	Very low	38.9%	↓	Serious	No	n < 50	No	Very low
	Maximum excursion	1 study of adequate quality (n = 14) [51]	0.68	–	Serious	No	*n* < 50	No	Very low	15.9%	?	Serious	No	n < 50	No	Very low
	Directional control	1 study of adequate quality (n = 14) [51]	0.71	+	Serious	No	n < 50	No	Very low	21.8%	?	Serious	No	n < 50	No	Very low
Physiomat-Trail-Making Task [53]	Score	1 study of adequate quality (*n* = 74) [53]	0.90	+	Serious	No	n = 50–100	No	Low	Not assessed						
	Sway Path	1 study of adequate quality (*n* = 47–73)^b^ [53]	0.47–0.82	+/− depending on condition	Serious	No	*n* = 50–100	No	Low	Not assessed						
	Time	1 study of adequate quality (n = 47–73)^b^ [53]	0.55–0.83	+/− depending on condition	Serious	No	n = 50–100	No	Low	Not assessed						
Physiomat-Follow-The-Ball Task	Sway Path	1 study of adequate quality (*n* = 73) [53]	0.84	+	Serious	No	n = 50–100	No	Low	Not assessed						
	Time	1 study of adequate quality (n = 73) [53]	0.79	+	Serious	No	*n* = 50–100	No	Low	Not assessed						
FR	Distance	2 studies of adequate quality (*n* = 29) [14, 51]	0.81–0.84	+	No	No	n < 50	No	Low	15.4–68.9%	?/↓	No	Yes	n < 50	No	Not assigned (inconsistency)
Hill Step Test	Number of steps	1 study of adequate quality (n = 14) [51]	0.87	+	Serious	No	n < 50	No	Very low	26.2%	?	Serious	No	n < 50	No	Very low
Step Quick Turn Test	Time	1 study of adequate quality (n = 14) [51]	0.55	–	Serious	No	n < 50	No	Very low	38.1%	↓	Serious	No	n < 50	No	Very low
	Sway	1 study of adequate quality (n = 14) [51]	0.64	–	Serious	No	n < 50	No	Very low	29.7%	?	Serious	No	n < 50	No	Very low
Figure of Eight Test	Time	1 study of adequate quality (*n* = 46)^a^ [17]	0.85–0.94	+	Serious	No	n < 50	No	Very low	36.9–37.9%	↓	Serious	No	n < 50	No	Very low
GMWT	Time	2 studies of adequate quality (*n* = 95)^a^ [43, 63]	0.93–0.99	+	No	No	n = 50–100	No	Moderate	19.6–31.2%	?/↓	No	No	n = 50–100	No	Moderate
	Number of oversteps	2 studies of adequate quality (n = 95)^a^ [43, 63]	0.57–0.96	?	No	Yes	n = 50–100	No	Not assigned (inconsistency)	33.3–225.7%	↓	No	Yes	n = 50–100	No	Not assigned (inconsistency)
BBS	Score	2 studies of adequate quality (n = 68) [14, 43]	0.95–0.99	+	No	No	n = 50–100	No	Moderate	10.2–38.6%	?/↓	No	No	n = 50–100	No	Moderate
Mobility and gait																
TUG	Time	6/5 studies of adequate quality (*n* = 200/191)^a^ [6, 14, 17, 43, 51, 102]	0.72–0.99	+	No	No	No	No	High	15.8–39.6%	+^h^/↓	No	No	No	No	High
Cognitive TUG	Time	1 study of adequate quality (n = 10) [51]	0.51	–	Serious	No	n < 50	No	Very low	36.2%	+^h^/↓	Serious	No	n < 50	No	Very low
Manual TUG	Time	1 study of adequate quality (n = 14) [51]	0.70	+	Serious	No	n < 50	No	Very low	26.7%	+^h^	Serious	No	n < 50	No	Very low
6 m WT	Walking speed	1 study of adequate quality (n = 58)^a^ [17]	0.83–0.89	+	Serious	No	n = 50–100	No	Low	31.6–41.5%	-^i^/↓	Serious	No	n = 50–100	No	Low
	Time	1 study of adequate quality (n = 9–10)^b^ [102]	0.92–0.95	+	Serious	No	*n* < 50	No	Very low	Not assessed						
	Number of steps	1 study of adequate quality (n = 9–10)^b^ [102]	0.80–0.90	+	Serious	No	n < 50	No	Very low	Not assessed						
4 m WT	Time	1 study of adequate quality (n = 53) [43]	0.85	+	Serious	No	n = 50–100	No	Low	84.3%	-^i^/↓	Serious	No	n = 50–100	No	Low
Instrumented gait analysis	Walking speed	4/3 studies of adequate quality (*n* = 93/85)^a, d, e^ [6, 26, 51, 121]	0.50–0.98	+ (except for NeuroCom Balance Master)	No	No	n = 50–100	No	Moderate	10.2–48.3%	+^i^/↓	No	No	n = 50–100	No	Moderate
	Step length	2 studies of adequate quality (*n* = 34)^a, d, e^ [51, 121]	0.75–0.98	+	No	No	n < 50	No	Low	7.0–35.6%	?/↓	No	No	n < 50	No	Low
	Step width	2 studies of adequate quality (n = 34) ^a, d, e^ [51, 121]	0.89–0.95	+	No	No	n < 50	No	Low	20.0–24.7%	?	No	No	n < 50	No	Low
	Stride length	2 studies/1 study of adequate quality (*n* = 28/20)^e^ [26, 121]	0.97–0.98	+	No	No	n < 50	No	Low	6.8–8.5%	?	Serious	No	n < 50	No	Very low
	Cadence	2 studies/1 study of adequate quality (n = 28/20)^e^ [26, 121]	0.88–0.91	+	No	No	n < 50	No	Low	7.1–7.5%	?	Serious	No	n < 50	No	Very low
	Swing time	2 studies/1 study of adequate quality (n = 28/20)^e^ [26, 121]	0.89–0.96	+	No	No	n < 50	No	Low	7.0–7.1%	?	Serious	No	n < 50	No	Very low
	Stance time	1 study of adequate quality (n = 20)^e^ [121]	0.70–0.73	+	Serious	No	n < 50	No	Very Low	8.6–8.7%	?	Serious	No	n < 50	No	Very low
	Toe in/out angle	1 study of adequate quality (n = 20)^e^ [121]	0.91–0.93	+	Serious	No	n < 50	No	Very Low	28.2–33.5%	?/↓	Serious	No	n < 50	No	Very low
	Walking speed variability	1 study of adequate quality (*n* = 16) [5]	0.66	–	Serious	No	*n* < 50	No	Very Low	77.8%	↓	Serious	No	n < 50	No	Very low
	Stride length variability	1 study of adequate quality (n = 16) [5]	0.80	+	Serious	No	*n* < 50	No	Very Low	71.7%	↓	Serious	No	n < 50	No	Very low
	Stride width variability	1 study of adequate quality (n = 16) [5]	0.83	+	Serious	No	*n* < 50	No	Very Low	46.9%	↓	Serious	No	n < 50	No	Very low
	Cadence variability	1 study of adequate quality (n = 16) [5]	0.65	–	Serious	No	n < 50	No	Very Low	41.4%	↓	Serious	No	n < 50	No	Very low
Strength																
5x STS	Time	2 studies/1 study of adequate quality (n = 24/14) [51, 102]	0.80–0.94	+	No	No	n < 50	No	Low	29.9%	?	Serious	No	n < 50	No	Very low
STS on NeuroCom Balance Master	Rising Index	1 study of adequate quality (n = 14) [51]	0.95	+	Serious	No	n < 50	No	Very low	21.8%	?	Serious	No	n < 50	No	Very low
	COG sway velocity	1 study of adequate quality (n = 14) [51]	0.02	–	Serious	No	n < 50	No	Very low	80.2%	↓	Serious	No	n < 50	No	Very low
Modified 30s CST	Repetitions	1 study of adequate quality (*n* = 52)^a^ [17]	0.79–0.88	+	Serious	No	n = 50–100	No	Low	33.2–45.7%	↓	Serious	No	*n* = 50–100	No	Low
Handgrip dynamometer	Force	3 studies/1 study of adequate quality (*n* = 143/57)^a^ [17, 102, 120]	0.42–0.98	+ (except for severe dementia)	No	No	No	No	High	34.9–36.8%	↓	Serious	No	n = 50–100	No	Low
Maximum isometric strength assessed with dynamometers	Peak force	1 studies of adequate quality (*n* = 11–12)^f^ [102]	0.63–0.71	?	Serious	Yes	n < 50	No	Not assigned (inconsistency)	Not assessed						
	(Normalised) torque	1 studies of adequate quality (n = 60)^a^ [114]	0.95–0.98	+	Serious	No	n = 50–100	No	Low	Not assessed						
Endurance																
6 min WT	Distance	2 studies/1 study of adequate quality (n = 84/51)^a, c^ [6, 118]	0.76–0.98	+	No	No	n = 50–100	No	Moderate	21.2–28.9%	?	Serious	No	n = 50–100	No	Low
	Walking speed	1 study of adequate quality (n = 33)^c^ [118]	0.75–0.89	+	Serious	No	n < 50	No	Very Low	Not assessed						
Functional performance																
E-ADL Test	Score	1 study of doubtful quality (*n* = 42) [110]	r = 0.73^g^	?	Very serious	No	n < 50	No	Very Low	Not assessed						

*4 m WT* 4-m walk test, *5x STS* Five Times Sit-to-Stand Test, *6 m WT* 6-m walk test, *6 min WT* 6-min walk test, *30s CST* 30-s chair stand test, *BBS* Berg Balance Scale, *COG* Centre of gravity, *E-ADL Test* Erlangen Test of Activities of Daily Living, *FICSIT-4* Frailty and Injuries: Cooperative Studies of Intervention Techniques - subtest 4, *FR* Functional Reach Test, *GMWT* Groningen Meander Walking Test, *ICC* Intraclass correlation coefficient, *MDC*_*95%*_ Percentage minimal detectable changes at 95% confidence interval, *n* Total number of participants, *STS* Sit-to-Stand, *TUG* Timed Up & Go Test.

Rating according to COSMIN criteria for good measurement properties: + = sufficient, − = insufficient,? = indeterminate, ↓ = unacceptable absolute test-retest reliability.

^a^ test-retest reliability was assessed for different subgroups, ^b^ test-retest reliability was assessed for different conditions, ^c^ test-retest reliability was assessed for 2 different raters and 2 different between-test intervals, ^d^ test-retest reliability was assessed with 2 different devices, ^e^ test-retest reliability was assessed with 2 analysis sets, ^f^ test-retest reliability was assessed for 3 muscle groups, ^g^ Spearman’s rank correlation coefficient, ^h^ minimal important change (TUG) = 10.1 s [17, 119], ^i^ minimal important change (walking speed) = 0.21 m/s [17, 119]

Most studies focused on between-day test-retest reliability, while some studies examined within-day and within-session test-retest reliability. Comparing these studies, ICC increased and MDC_95%_ decreased, respectively, from between-day (ICC = 0.02–0.99, MDC_95%_ = 6.8–225.7% [5, 14, 17, 43, 51, 53, 63, 102, 118, 120, 121]), through within-day (ICC = 0.79–0.99, MDC_95%_ = 21.1–30.0% [6, 26, 118]), to within-session test-retest reliability (ICC = 0.95–0.98 [114]).

##### Balance

Six investigations assessing test-retest reliability of eleven balance assessments determined ICC and MDC_95%_ ranging between 0.32–0.99 and 10.2–225.7%, respectively [14, 17, 43, 51, 53, 63]. Relative test-retest reliability was sufficient for all balance assessments except for Limits of Stability, Step Quick Turn Test, and simple condition of Physiomat-Trail-Making Task. However, quality of evidence for relative test-retest reliability was low or very low for most assessments. Only GMWT (time) and BBS reached moderate quality of evidence. Absolute test-retest reliability for balance assessments was indeterminate or unacceptable with moderate to very low quality of evidence (see Table 3).

GMWT (time) and BBS showed the highest ICC, while we could not observed a clear tendency for MDC_95%_. Comparing different outcomes of GMWT, ICC were higher and MDC_95%_ were lower for time than for number of oversteps (see Table 3).

##### Mobility and gait

Nine studies investigated test-retest reliability of six mobility and gait assessments. They reported ICC between 0.50 and 0.99 and MDC_95%_ from 6.8 to 84.3% [5, 6, 14, 17, 26, 43, 51, 102, 121]. Relative test-retest reliability was sufficient for TUG, manual TUG, 6 m WT, 4 m WT, and instrumented gait analysis (except for cadence variability, walking speed variability, and walking speed assessed with NeuroCom Balance Master), while it was insufficient for cognitive TUG. Quality of evidence for relative test-retest reliability was high for TUG, moderate to very low for instrumented gait analysis, and low or very low for all other assessments. Absolute test-retest reliability was indeterminate for spatiotemporal gait parameters, insufficient/unacceptable for variability gait parameters, 4 m WT, and 6 m WT, and sufficient for manual TUG. For TUG, cognitive TUG, and walking speed assessed with instrumented gait analysis, absolute test-retest reliability was sufficient according to COSMIN criteria but unacceptable when applying MDC_95%_ limit of 30%. Except for TUG and walking speed assessed with instrumented gait analysis (high/moderate quality of evidence), quality of evidence for absolute test-retest reliability was low or very low (see Table 3).

Considering up and go tasks, ICC were higher for single than for dual task conditions. Focusing on short distance walk tests (WT), MDC_95%_ were lower for 6 m WT than for 4 m WT. Furthermore, the comparison of different gait parameters assessed with instrumented gait analysis, determined lower ICC and higher MDC_95%_ for variability measures than for spatiotemporal gait parameters. Comparing different assessments to determine short distance walking speed showed higher ICC and lower MDC_95%_ for instrumented gait analysis (except for NeuroCom Balance Master) than for simple short distance WT (see Table 3).

##### Strength

Five studies focusing on test-retest reliability of strength assessments reported ICC and MDC_95%_ ranging between 0.02–0.98 and 21.8–80.2%, respectively [17, 51, 102, 114, 120]. Relative test-retest reliability was sufficient for modified 30s CST, 5x STS, handgrip dynamometers (except for severe dementia and one-time measuring), and maximum isometric strength assessed with dynamometers (except for dorsiflexor and iliopsoas muscle strength), while it was insufficient for STS on NeuroCom Balance Master (except for Rising Index). Quality of evidence for relative test-retest reliability was high for handgrip dynamometers and low or very low for all other strength assessments. Absolute test-retest reliability was indeterminate for 5x STS and Rising Index of STS on NeuroCom Balance Master, and unacceptable for modified 30s CST, centre of gravity sway velocity of STS on NeuroCom Balance Master, and handgrip dynamometers. Quality of evidence for absolute test-retest reliability was low or very low for all assessments (see Table 3).

Comparing different STS assessments, ICC for assessments performing only one STS repetition were lower (except for Rising Index) than STS assessments with more repetitions. Moreover, MDC_95%_ increased from 5x STS, through modified 30s CST, to STS on NeuroCom Balance Master (except for Rising Index) (see Table 3).

##### Endurance

Considering endurance, test-retest reliability was only determined for 6 min WT. Two studies observed ICC between 0.75 and 0.98, while MDC_95%_ ranged from 21.2 to 28.9% [6, 118]. Accordingly, relative test-retest reliability was sufficient with moderate to very low quality of evidence. Absolute test-retest reliability was indeterminate with low quality of evidence (see Table 3).

##### Functional performance

Functional performance was rarely assessed. One study focusing on the E-ADL Test did not determine ICC and MDC_95%_, but found significant correlations for the whole test (r = 0.73) and separate items (r = 0.35–0.63) [110]. Quality of evidence was very low.

#### Influence of severity and aetiology of dementia and cueing on test-retest reliability

With respect to severity of dementia, the Frailty and Injuries: Cooperative Studies of Intervention Techniques - subtest 4 (FICSIT-4) and GMWT tend to yield higher ICC and/or lower MDC_95%_ with less cognitive impairment. In contrast, ICC were slightly higher and/or MDC_95%_ lower with stronger cognitive impairment for BBS, 6 m WT, modified 30s CST, and 5x STS (see Table 4).

**Table 4:** Subgroup analysis of test-retest reliability considering severity of dementia

	Mild dementia	Mild to moderate dementia	Moderate dementia	Severity not reported
FICSIT-4	MMSE [mean (SD)]: 22.7 (2.1) ICC = 0.82 MDC_95%_ = 58.9% [17]	MMSE [mean (SD)]: 19.2 (4.4) ICC = 0.79 MDC_95%_ = 59.4% [17]	MMSE [mean (SD)]: 15.5 (2.4) ICC = 0.80 MDC_95%_ = 71.1% [17]	
GMWT	MMSE [mean (SD)]: n.r. ICC = 0.79–0.96 MDC_95%_ = n.r [63].	MMSE [mean (SD)]: 17.4 (4.3) ICC = 0.63–0.94 MDC_95%_ = 31.2–225.7% [63]	MMSE [mean (SD)]: n.r. ICC = 0.57–0.93 MDC_95%_ = n.r [63].	MMSE [mean (SD)]: 13.8 (5.7) ICC = 0.96–0.99 MDC_95%_ = 19.6–33.3% [43]
BBS		MMSE [mean (SD)]: 20.0 (5.5) ICC = 0.95 MDC_95%_ = 38.6% [14]		MMSE [mean (SD)]: 13.8 (5.7) ICC = 0.99 MDC_95%_ = 10.2% [43]
6 m WT	MMSE [mean (SD)]: 22.7 (2.1) ICC = 0.83 MDC_95%_ = 41.5% [17]	MMSE [mean (SD)]: 19.2 (4.4) ICC = 0.86 MDC_95%_ = 36.5% [17]	MMSE [mean (SD)]: 15.5 (2.4) ICC = 0.89 MDC_95%_ = 31.6% [17]	MMSE [mean (SD)]: 16.9 (7.3) ICC = 0.80–0.95 MDC_95%_ = n.r [102].
5x STS	MMSE [mean (SD)]: 21.4 (5.0) ICC = 0.80 MDC_95%_ = 29.9% [51]			MMSE [mean (SD)]: 16.9 (7.3) ICC = 0.94 MDC_95%_ = n.r [102].
Modified 30s CST	MMSE [mean (SD)]: 22.7 (2.1) ICC = 0.79 MDC_95%_ = 45.7% [17]	MMSE [mean (SD)]: 19.2 (4.4) ICC = 0.84 MDC_95%_ = 42.5% [17]	MMSE [mean (SD)]: 15.5 (2.4) ICC = 0.88 MDC_95%_ = 33.2% [17]	

*5x STS* Five Times Sit-to-Stand Test, *6 m WT* 6-m walk test, *30s CST* 30-s chair stand test, *BBS* Berg Balance Scale, *FICSIT-4* Frailty and Injuries: Cooperative Studies of Intervention Techniques - subtest 4, *GMWT* Groningen Meander Walking Test, *ICC* Intraclass correlation coefficient, *MDC*_*95%*_ Percentage minimal detectable changes at 95% confidence interval, *MMSE* Mini-Mental State Examination, *n.r.* Not reported, *SD* Standard deviation.

Regarding aetiology of dementia, maximum isometric strength assessed with dynamometers and short distance walking speed (except for instrumented gait analysis with NeuroCom Balance Master) resulted in somewhat higher ICC and/or lower MDC_95%_ for AD vs. various or not reported types. In contrast, ICC were slightly higher and/or MDC_95%_ were lower for various or not reported types vs. AD for BBS, TUG (between-day reliability), up and go tasks in general (between-day reliability), 5x STS, and STS tasks in general (except for Rising Index) (see Table 5).

**Table 5:** Subgroup analysis of test-retest reliability considering aetiology of dementia

	Alzheimer’s disease	Various types/not reported
BBS	ICC = 0.95 MDC_95%_ = 38.6% [14]	ICC = 0.99 MDC_95%_ = 10.2% [43]
TUG (between-day reliability)	ICC = 0.72–0.76 (MDC_95%_ = 20.3–24.9%) [14, 51]	ICC = 0.87–0.99 (MDC_95%_ = 15.8–39.6%) [17, 43, 102]
Up and go tasks (between-day reliability)	ICC = 0.51–0.76 (MDC_95%_ = 20.3–36.2%) [14, 51]	ICC = 0.87–0.99 (MDC_95%_ = 15.8–39.6%) [17, 43, 102]
Short distance walking speed (without NeuroCom Balance Master)	ICC = 0.95–0.98 MDC_95%_ = 10.2–28.9% [6, 121]	ICC = 0.83–0.95 MDC_95%_ = 31.6–84.3% [17, 26, 43]
5x STS	ICC = 0.80 MDC_95%_ = 29.9% [51]	ICC = 0.94 MDC_95%_ = n.r [102].
STS assessments (without Rising Index)	ICC = 0.02–0.80 MDC_95%_ = 29.9–80.2% [51]	ICC = 0.79–0.94 MDC_95%_ = 33.2–45.7% [17, 102]
Maximum isometric strength assessed with dynamometers	ICC = 0.95–0.98 MDC_95%_ = n.r [114].	ICC = 0.63–0.71 MDC_95%_ = n.r [102].

*5x STS* Five Times Sit-to-Stand Test, *BBS* Berg Balance Scale, *ICC* Intraclass correlation coefficient, *MDC*_*95%*_ Percentage minimal detectable changes at 95% confidence interval, *n.r.* Not reported, *STS* Sit-to-Stand, *TUG* Timed Up & Go Test.

Considering cueing, GMWT and TUG showed somewhat higher ICC and/or lower MDC_95%_ when cueing was allowed or more extensive. In contrast, ICC were slightly higher and/or MDC_95%_ were lower for no cueing or less extensive cueing in FR, short distance WT, and short distance walking speed (see Table 6).

**Table 6:** Subgroup analysis of test-retest reliability considering cueing

	No cueing	Verbal cueing or verbal and visual/tactile cueing	More extensive cueing including physical assistance
FR		ICC = 0.84 MDC_95%_ = 15.4% [51]	ICC = 0.81 MDC_95%_ = 68.9% [14]
GMWT	ICC = 0.57–0.96 MDC_95%_ = 31.2–225.7% [63]		ICC = 0.96–0.99 MDC_95%_ = 19.6–33.3% [43]
TUG		ICC = 0.76–0.96 MDC_95%_ = 23.3–39.6% [17, 51, 102]	ICC = 0.72–0.99 MDC_95%_ = 15.8–30.0% [6, 14, 43]
Short distance WT		ICC = 0.80–0.95 MDC_95%_ = 31.6–41.5% [17, 102]	ICC = 0.85 MDC_95%_ = 84.3% [43]
Short distance walking speed	ICC = 0.95–0.96 MDC_95%_ = 10.2–12.0% [121]	ICC = 0.50–0.95 MDC_95%_ = 31.6–48.3% [17, 26, 51]	ICC = 0.85–0.98 MDC_95%_ = 25.5–84.3% [6, 43]

*FR* Functional Reach Test, *GMWT* Groningen Meander Walking Test, *ICC* Intraclass correlation coefficient, *MDC*_*95%*_ Percentage minimal detectable changes at 95% confidence interval, *TUG* Timed Up & Go Test, *WT* Walk tests.

### Frequency of use and effect sizes of motor assessments applied in previous randomised controlled trials

TUG, BBS, 5x STS, POMA, 30s CST, and instrumented gait analysis, were the most frequently applied assessments, utilised in six to 16 RCT. We were only able to calculate effect sizes for 12 studies, as F/t statistics and/or standard deviations of baseline-post differences were infrequently reported. Effect sizes were large for FR, BBS, POMA, TUG, instrumented gait analysis, 5x STS, ACSID, and 30s CST (see Table 1/Additional file 9 for motor assessments identified during first search without available information on psychometric properties).

### Summary and derivation of recommendations

Aiming to derive comprehensive recommendations on motor assessments for IWD, we combined the results of primary and secondary outcomes for each physical domain as summarised in Table 7.

**Table 7:** Summary of outcomes to derive recommendations for motor assessments for individuals with dementia

Motor assessment	Inter-rater reliability		Test-retest reliability		Frequency of use	Time^*^group interaction effect size
	relative	absolute	relative	absolute		
Balance						
FICSIT-4	?	?	0	–	–	?
Modified Clinical Test of Sensory Interaction of Balance	?	?	0	–	–	?
Limits of Stability	?	?	–	–	–	?
Physiomat-Trail-Making Task	?	?	0	?	–	?
Physiomat-Follow-The-Ball Task	?	?	0	?	–	?
FR	0	?	0	0	0	+
Hill Step Test	?	?	0	0	0	?
Step Quick Turn Test	?	?	–	–	–	?
Figure of Eight Test	?	?	0	–	–	?
GMWT	0	0	+	0	–	?
BBS	+	0	+	0	+	+
Modified BBS	?	?	?	?	–	?
POMA	?	?	?	?	+	+
Mobility and gait						
TUG	+	+	+	+	+	+
Cognitive TUG	?	?	–	0	0	?
Manual TUG	?	?	+	0	–	?
6 m WT	0	0	0	–	0	?
4 m WT	0	–	0	–	0	0
Instrumented gait analysis	?	?	0	0	+	+
Strength						
5x STS	?	?	0	0	+	+
STS on NeuroCom Balance Master	?	?	–	–	–	?
ACSID	+	?	?	?	–	+
30s CST	0	0	0	–	+	+
Handgrip dynamometer	?	?	+	–	0	–
Maximum isometric strength assessed with dynamometers	?	?	0	?	0	?
Endurance						
6 min WT	0	?	+	0	0	?
Functional performance						
SPPB	?	?	?	?	0	0
E-ADL Test	?	?	0	?	0	?

*4 m WT* 4-m walk test, *5x STS* Five Times Sit-to-Stand Test, *6 m WT* 6-m walk test, *6 min WT* 6-min walk test, *30s CST* 30-s chair stand test, *ACSID* Assessment of Compensatory Sit-to-Stand Maneuvers in People With Dementia, *BBS* Berg Balance Scale, *E-ADL Test* Erlangen Test of Activities of Daily Living, *FICSIT-4* Frailty and Injuries: Cooperative Studies of Intervention Techniques - subtest 4, *FR* Functional Reach Test, *GMWT* Groningen Meander Walking Test, *POMA* Performance Oriented Mobility Assessment, *SPPB* Short Physical Performance Battery, *STS* Sit-to-Stand, *TUG* Timed Up & Go Test.

*Relative reliability*: - = insufficient, 0 = sufficient, very low/low quality of evidence, + = sufficient, moderate/high quality of evidence,? = not investigated.

*Absolute reliability*: - = insufficient/unacceptable, 0 = indeterminate/inconsistent/sufficient, very low/low quality of evidence, + = sufficient, moderate/high quality of evidence,? = not investigated.

*Frequency of use*: - = 1 randomised controlled trial, 0 = 2–5 randomised controlled trials, += > 5 randomised controlled trials.

Time^*^group interaction effect size: - = no effect, 0 = at least one trial with small or medium effect, + = at least one trial with large effect,? = could not be calculated/not reported.

Considering all information on primary and secondary outcomes, the derived recommendations include the following motor assessments:

- Balance: FR, GMWT (time), BBS, and POMA
- Mobility and gait: TUG and instrumented gait analysis to assess spatiotemporal gait parameters
- Strength: STS assessments with more than one repetition
- Endurance: 6 min WT
- Functional Performance: No recommendation possible, due to insufficient research on psychometric properties

These recommendations are based on several outcomes rated in the highest category or one outcome rated in the highest and at least two in the second category (see Table 7).

## Discussion

We addressed the purpose of this systematic review to quantitatively examine motor assessments for IWD by comprehensively analysing psychometric properties (primary outcome), frequency of use, and effect sizes (secondary outcomes) in a two-stage literature search. Recommendations on motor assessments are based on primary and secondary outcomes. Additionally, we analysed the influence of severity and aetiology of dementia and cueing on test-retest reliability.

### Findings on primary and secondary outcomes

The systematic search identified only few investigations examining validity, internal consistency, and intra-rater reliability of motor assessments in IWD. Thus, we were not able to draw further conclusions or consider these outcomes for deriving recommendations. Summarizing findings for inter-rater reliability shows sufficient relative inter-rater reliability and relatively low MDC_95%_ of considered motor assessments. Hence, they are objective measures to determine motor performance in IWD. Motor assessments analysing time in tasks of short duration, such as 4 m WT, should, however, be treated with caution, as small measurement errors may significantly influence absolute inter-rater reliability. With respect to test-retest reliability, the majority of identified investigations observed sufficient relative test-retest reliability, while absolute test-retest reliability was mainly indeterminate or unacceptable. This supports their usage to investigate changes on a group level, but does not allow assessing intra-individual changes [7, 17, 31]. Moreover, decreasing test-retest reliability from between-day, through within-day, to within-session investigations may be related to fluctuating daily forms in IWD. We expect that characteristics of daily form, such as mood or motivational aspects, remain relatively constant within short intervals, while they potentially alter with increasing time. More research is necessary to develop criteria to determine daily form, aiming to ensure comparable conditions in longitudinal investigations. Besides, fluctuating daily forms in IWD may have contributed to observed unacceptable absolute test-retest reliability. Other explanations refer to high intra-individual variability in IWD and related inappropriate or naive selection of metrics, which do not account for this variability.

Regarding frequency of use, previous trials predominately applied clinical motor assessments established in healthy older adults or various clinical populations, while those considering specific characteristics of IWD such as GMWT, Physiomat, or ACSID, were less frequently applied. This may be related to their first introduction between 2014 and 2018. Due to insufficient information in previous RCT, we were only able to determine time*group interaction effect sizes for 38% of analysed motor assessments. Based on large effect sizes reported in at least one RCT, we assumed sensitivity to change for most of these assessments.

### Findings on influence of severity and aetiology of dementia and cueing on test-retest reliability

Considering severity of dementia, we expected decreasing test-retest reliability with increasing cognitive impairment. This assumption was true for FICSIT-4 and GMWT but not for all assessments. Severity of dementia may only influence specific assessments, for example those with complex instructions or assessing outcomes frequently impaired in IWD, such as balance [10]. Unexpectedly, we observed increasing test-retest reliability with increasing severity of dementia for BBS, 6 m WT, modified 30s CST, and 5x STS. However, these observations were only based on single studies, which partly differed in characteristics, such as aetiology of dementia.

Regarding the aetiology of dementia, test-retest reliability of BBS and up and go tasks was lower for AD than for various or not reported types. Both assessments consist of several short tasks and include multi-step instructions. Compared to other aetiologies, individuals with AD may have more difficulties in understanding and/or remembering such instructions, which potentially influences test-retest reliability [14, 23, 122]. In contrast, test-retest reliability of walking speed was higher in AD which could be related to later occurring gait impairments in AD [20]. Additional research on aetiologies, however, is required to understand lower test-retest reliability of STS tasks and higher test-retest reliability of maximum isometric strength assessed with dynamometers in AD.

Analysing the influence of cueing on test-retest reliability revealed higher test-retest reliability when cueing was allowed or more extensive for GMWT and TUG, which are assessments consisting of unfamiliar or several short tasks. Cueing possibly stabilises motor performance by supporting impaired cognitive performance and thus improves test-retest reliability. In contrast, short distance WT, for which test-retest reliability was higher when cueing was not allowed or less extensive, are close to everyday life, include single-stage tasks, and consider well automated movement processes not requiring additional cognitive support. Accordingly, cueing rather may distract IWD leading to destabilised performance decreasing test-retest reliability. No explanation for the same association in FR is available.

Based on these observed influences, we derived the following suggestions:

- Put emphasis on simple instructions, especially for IWD with advanced stages or AD.
- Consider individual cognitive and motor deficits, when selecting motor assessments.
- Only use cueing for motor assessments where it is inevitable.

### Recommendations and need for future research

Recommendations for balance assessments include FR, GMWT (time), BBS, and POMA. Due to infrequent use and insufficient research on psychometric properties, feasibility and sensitivity to change of GMWT and psychometric properties of POMA require further investigation. Focusing on mobility and gait, we suggest to apply TUG and spatiotemporal gait parameters assessed with instrumented gait analysis. Comparing different gait analysis systems, NeuroCom Balance Master, however, seems to be less suitable. Despite insufficient or equivocal results, future research should investigate short distance WT of different distances, as instrumented gait analysis systems may not be available for all studies. Considering strength, we suggest to apply STS assessments comprising more than one repetition, which, however, predominately determine functional performance of lower limbs. Thus, further evaluation of strength assessments including upper limb strength and measures allowing conclusion on actual strength performance are required. Moreover, we suggest to use the 6 min WT as an endurance assessment for IWD. Future research on endurance assessment, however, is crucial since this was the only identified assessment. As information on psychometric properties is insufficient, we are not able to recommend any functional performance assessment. Based on secondary outcomes some indications are available for SPPB. However, psychometric properties of SPPB and other functional performance assessments need to be investigated in future studies.

### Comparison with state of research

Recommendations of motor assessments in this review are largely in line with those of previous reviews [13, 24]. Small discrepancies may be related to distinctions in identified assessments and studies, different prioritisation of considered outcomes, and divergent criteria for good measurement properties. Additionally, this review, consistently to Fox et al. [7], determined sufficient relative test-retest reliability for the majority of motor assessments in IWD, but remarked high MDC_95%_ reflecting unacceptable absolute test-retest reliability.

Similarly, motor assessments recommended in this review are mainly in line with those elaborated in a qualitative approach [22]. However, FICSIT, 6 m WT, SPPB, and Physical Performance Test were rated appropriate in the qualitative approach, but could not be recommended based on quantitative outcomes as they were infrequently used or insufficiently investigated. Further discrepancies on FR, which was rated inappropriate but can be recommended based on quantitative outcomes, require additional examination. Moreover, some general indications, related to consideration of specific characteristics and cueing are consistently suggested. Accordingly, this review largely sustains the recommendations elaborated in a qualitative approach.

### General considerations on primary and secondary outcomes

The interpretation of findings regarding psychometric properties is challenging as there are no firm criteria for acceptable reliability in literature [31]. Regardless of concrete criteria, ICC do not only reflect relative reliability but also can be related to sample size or variability in the sample [123]. Accordingly, trial-to-trial consistency can be poor, despite high ICC. Thus, it is advised not to focus on single estimates of reliability and to additionally consider absolute reliability [17, 31]. Due to lack of information on minimal important change of motor assessments in IWD, we could scarcely apply COSMIN criteria for absolute reliability. Besides, Smidt et al. [42] arbitrarily defined that a difference of 10% in minimal detectable change would be acceptable. Other research groups referred to them and introduced another cut-off of 30% without any justification [43, 44]. In absence of other criteria, we adopted this cut-off of 30% to identify unacceptable MDC_95%_ but not to conclude on sufficient absolute reliability.

Frequency of use and effect sizes do not necessarily allow conclusions to be drawn on quality of motor assessments and should not be overestimated. Regardless of appropriateness and meaningfulness, researchers may decide to apply motor assessments as they are commonly used or easy to utilise. Nonetheless, frequency of use can provide indications about feasibility of motor assessments, which is based on the assumption that unfeasible motor assessments do not disseminate as good as feasible ones. Comparably, effect sizes can provide information on sensitivity to change, but are also dependent on effectiveness of interventions.

### Strengths and limitations

To our knowledge, this is the first systematic review utilising a comprehensive approach combining different outcomes of previous reviews by performing an extensive two-stage literature search. We need to state potential risk of bias regarding the selection of considered motor assessments. Due to restricting the analysis of motor assessments to those applied in RCT, some assessments may be missing. Furthermore, large heterogeneity of included psychometric property studies limits the meaningfulness of derived recommendations. As psychometric properties are potentially influenced by various determinants, such as sample size, sample characteristics including severity and aetiology of dementia, cueing, test-retest interval, or considered outcomes, we cannot ensure that the deductions on psychometric properties are true and not randomly caused by differing determinants. Therefore, false assumptions, undetected influences or relations, and random observations may have occurred. Similarly, the consideration of several influences on test-retest reliability only allows rough estimations, which could be also affected by heterogeneity of analysed studies. Moreover, insufficient information on execution of motor assessments, severity and aetiology of dementia, and cueing in available investigations impeded detailed analyses and limited meaningfulness of observations. Accordingly, the elaborated recommendations should be used with care and further research investigating psychometric properties and dementia specific influences on test-retest reliability is required.

## Conclusion

Despite the necessity for further research in various areas, this review establishes an important foundation for future investigations. Additionally, direct implications for studies determining effectiveness of physical activity on motor performance in IWD can be derived. However, elaborated recommendations cannot be considered as final conclusions since the analysis of primary and secondary outcomes reveals several challenges and areas of insufficient research, and only focus on quantitative aspects. Furthermore, new assessments, especially developed for IWD, are required. Such assessments can be based on prior tasks but should consider specific characteristics of IWD. Additionally, it is of high importance to standardise motor assessments and cueing to ensure comparability between studies. Herein, standardisation refers to selection and performance procedures of motor assessments and external cues. Currently, a wide range of motor assessments (e.g. previous RCT applied 19 different balance assessments) with different performance procedures (e.g. different ratings or modifications) as well as various external cues (e.g. clearly defined verbal cues vs. as much assistance as needed) are frequently applied to determine the same motor functions or quantities. Accordingly, recommendations on specific motor assessments as well as indications on assessment procedures elaborated in quantitative and qualitative (see [22]) approaches are important to improve standardisation. Evidence on effectiveness of physical activity can contribute to gain access to physical activity interventions and thereby positively influence quality of life in IWD. Determining evidence, however, is not possible without appropriate, sensitive, valid, reliable, and standardised motor assessments, which consider the individual characteristics of single individuals.

## Supplementary information

**Additional file 1.** Search term first search.

**Additional file 2.** Formulas for calculating time*group interaction effect sizes.

**Additional file 3.** Search term main search.

**Additional file 4.** Formulas for calculating minimal detectable change at 95% confidence interval.

**Additional file 5.** Study characteristics first search.

**Additional file 6.** Study characteristics main search.

**Additional file 7.** Data extraction first search.

**Additional file 8.** Data extraction main search.

**Additional file 9.** Description, frequency of use, and effect sizes of motor assessments applied in previous randomised controlled trials without available information on psychometric properties.

## Data Availability

Not applicable.

## Abbreviations

30 s CST: 30-s chair stand test
4 m WT: 4-m walk test
5x STS: Five Times Sit-to-Stand Test
6 m WT: 6-m walk test
6 min WT: 6-min walk test
ACSID: Assessment of Compensatory Sit-to-Stand Maneuvers in People With Dementia
AD: Alzheimer’s disease
BBS: Berg Balance Scale
COSMIN: COnsensus-based Standards for the selection of health Measurement INstruments
E-ADL Test: Erlangen Test of Activities of Daily Living
FICSIT-4: Frailty and Injuries: Cooperative Studies of Intervention Techniques - subtest 4
FR: Functional Reach Test
GMWT: Groningen Meander Walking Test
ICC: Intraclass correlation coefficient/s
IWD: Individuals with dementia
MDC_95%_: Percentage minimal detectable change/s at 95% confidence interval
MMSE: Mini-Mental State Examination
POMA: Performance Oriented Mobility Assessment
RCT: Randomised controlled trial/s
SPPB: Short Physical Performance Battery
STS: Sit-to-Stand/sit-to-stand
TUG: Timed Up & Go Test
WT: Walk test/s

## Footnotes

1 defined as all types of physical activity that are planned, structured, repetitive, and purposive aiming to improve or maintain one or more components of physical fitness [33]

2 This summary utilises psychometric property terms indicated in original studies. These terms have not been consistently used throughout the literature and should have been adapted according to the COSMIN checklist [41].
