# Supplementary material for: Recommended motor assessments based on psychometric properties in individuals with dementia: a systematic review
Source: Eur Rev Aging Phys Act. 2019 Nov 3;16:20. doi: 10.1186/s11556-019-0228-z (PMC6825725; doi:10.1186/s11556-019-0228-z)
Supplement: Supplementary file 1 — Additional file 1. Search term first search. [file 11556_2019_228_MOESM1_ESM.pdf]

Additional file 1

Search term first search

Details of the search strategy used in Pubmed are provided below. This search was modified as appropriate for other databases. All fields were searched for MeSH and free search terms:

1. dementia
2. “Alzheimer disease”
3. “Vascular dementia”
4. or/1-3
5. “physical activity”
6. “motor activity”
7. exercise
8. “physical training”
9. training
10. or/5-9
11. “functional performance”
12. fitness
13. “physical fitness”
14. balance
15. equilibrium
16. gait
17. mobility
18. strength
19. flexibility
20. endurance
21. or/11-20
22. 4 and 10 and 21
