# Supplementary material for: Recommended motor assessments based on psychometric properties in individuals with dementia: a systematic review
Source: Eur Rev Aging Phys Act. 2019 Nov 3;16:20. doi: 10.1186/s11556-019-0228-z (PMC6825725; doi:10.1186/s11556-019-0228-z)
Supplement: Supplementary file 2 — Additional file 2. Formulas for calculating time*group interaction effect sizes. [file 11556_2019_228_MOESM2_ESM.pdf]

Additional file 2

Formulas for calculating time*group interaction effect sizes

$$d = \sqrt{F\left(\frac{n_{IG} + n_{CG}}{n_{IG} * n_{CG}}\right) * \left(\frac{n_{IG} + n_{CG}}{n_{IG} + n_{CG} - 2}\right)}$$

$$d = t\sqrt{\left(\frac{n_{IG} + n_{CG}}{n_{IG} * n_{CG}}\right) * \left(\frac{n_{IG} + n_{CG}}{n_{IG} + n_{CG} - 2}\right)}$$

$$d = \frac{mean_{DIFF\ IG} - mean_{DIFF\ CG}}{\sqrt{\frac{(n_{IG} - 1) * SD^2_{DIFF\ IG} + (n_{CG} - 1) * SD^2_{DIFF\ CG}}{n_{IG} + n_{CG}}}}$$

with d=Cohen's d effect size, F=F statistic, $mean_{DIFF\ CG}$=baseline-post mean difference in control group, $mean_{DIFF\ IG}$=baseline-post mean difference in intervention group, n_{CG}=number of subjects in control group, n_{IG}= number of subjects in intervention group, $SD^2_{DIFF\ CG}$=standard deviation of baseline-post difference in control group, $SD^2_{DIFF\ IG}$= standard deviation of baseline-post difference in intervention group, t=t statistic [34].

References

34. Thalheimer W, Cook S. How to calculate effect sizes from published research: A simplified methodology. 2002.
