# Supplementary material for: Recommended motor assessments based on psychometric properties in individuals with dementia: a systematic review
Source: Eur Rev Aging Phys Act. 2019 Nov 3;16:20. doi: 10.1186/s11556-019-0228-z (PMC6825725; doi:10.1186/s11556-019-0228-z)
Supplement: Supplementary file 3 — Additional file 3. Search term main search. [file 11556_2019_228_MOESM3_ESM.pdf]

Additional file 3

Search term main search

Details of the search strategy used in Pubmed are provided below. This search was modified as appropriate for other databases. Title and abstract were searched for MeSH and free search terms:

1. dementia
2. Alzheimer*
3. “vascular dementia”
4. “frontotemporal disease”
5. or/1-4
6. validity
7. valid
8. “content validity”
9. “structural validity”
10. “criterion validity”
11. reliability
12. reliable
13. consistency
14. “Cronbach’s alpha”
15. reproducibility
16. repeatability
17. “intra-rater”
18. “intra rater”
19. “inter-rater”
20. “inter rater”
21. “relative reliability”
22. correlation
23. kappa
24. “intra class correlation”
25. “intra class”
26. “intra-class”
27. ICC
28. “limits of agreement”
29. LOA
30. “absolute reliability”
31. “standard error of measurement”
32. SEM
33. “minimal detectable change”
34. MDC
35. “smallest detectable change”
36. SDC
37. or/6-36

72. “TUGT”
73. “timed get-up-and-go test”
74. “TGUG”
75. “modified timed up and go”
76. “TUG mod”
77. “get up and go test”
78. “get-up-and-go test”
79. “timed up and go test with a secondary cognitive”
80. “timed up and go test with a secondary motor”
81. “6-meter walk test”
82. “six-meter walking test”
83. “6-meter walking speed”
84. “10-m walk test”
85. “walking speed over 10 m”
86. “8-ft walk test”
87. “Timed 8-foot walk”
88. “4-m usual gait speed”
89. “gait analysis”
90. “gait performance”
91. “Bessou locometer”
92. “Southampton Assessment of Mobility”
93. “Southampton Mobility Assessment”
94. “Hierarchical Assessment of Balance and Mobility”
95. “HABAM”
96. “Sit-to-Stand”
97. “STS”
98. “Chair sit to stand test”
99. “CST”
100. “chair rise test”
101. “Timed Chair Stands”
102. “5-time-sit-to-stand test”
103. “5-chair-stand”
104. “30-second chair stand test”
105. “30-s chair stand test”
106. “30-second sit-to-stand test”
107. “stair-climbing performance”
108.” “arm curl test”

38. “near-tandem test”
39. “single leg stance”
40. “SLS”
41. “one-leg balance test”
42. “One Leg Standing Balance Test”
43. “OLST”
44. “frailty and injuries cooperative studies of intervention techniques – subtest 4”
45. “FICSIT-4”
46. “Posturography platform”
47. “Wii Balance Board”
48. “NeuroCom Balance Master”
49. “functional reach test”
50. “functional reach”
51. “FR”
52. “Hill Step Test”
53. “Step test”
54. “figure of eight test”
55. “Groningen meander walking test”
56. “Berg Balance Scale”
57. “BERG”
58. “BBS”
59. “modified Berg Balance Scale”
60. “m-BBS”
61. “Performance Oriented Mobility Assessment”
62. “POMA”
63. “Performance Oriented Motor Assessment”
64. “Tinetti’s Performance Oriented Motor Assessment”
65. “Tinetti scale”
66. “Tinetti Test”
67. “TT”
68. “Tinetti balance assessment”
69. “Tinetti Balance Evaluation Test”
70. “timed up and go”
71. “TUG”
109. “handgrip”
110. “dynamometer
111. “one-repetition maximum”
112. “1-RM”
113. “leg press”
114. “Physical therapy assessment”
115. “2-min walk test”
116. “Two Minute Walking Test”
117. “6-minute walk test”
118. “6-minute walking test”
119. “6WT”
120. “The 6-Minute Walk”
121. “Modified 6-Minute Walk”
122. “400-m walk test”
123. “3-speed walking test”
124. “6-min Astrand Cycle Ergometer test”
125. “Ergometric test and rest electrocardiogram”
126. “ECG”
127. “pedal power”
128. “Chair sit and reach”
129. “Short Physical Performance Battery”
130. “SPPB”
131. “Physical Performance Test”
132. “PPT”
133. “7-item Physical Performance Test”
134. “PPT-7”
135. “Erlangen-ADL test”
136. “E-ADL”
137. “Senior Fitness test”
138. “Jebsen hand function test”
139. “JHFT”
140. “Jebsen Total Time”
141. “JTT”
142. “Physiological Profile Assessment”
143. “PPA”
144. or/38-143
145. 5 and 37 and 144
