# Supplementary material for: Recommended motor assessments based on psychometric properties in individuals with dementia: a systematic review
Source: Eur Rev Aging Phys Act. 2019 Nov 3;16:20. doi: 10.1186/s11556-019-0228-z (PMC6825725; doi:10.1186/s11556-019-0228-z)
Supplement: Supplementary file 4 — Additional file 4. Formulas for calculating minimal detectable change at 95% confidence interval. [file 11556_2019_228_MOESM4_ESM.pdf]

Additional file 4

Formulas for calculating minimal detectable change at 95% confidence interval

$$MDC_{95\%} = \frac{MDC_{95}}{mean} * 100$$

$$MDC_{95\%} = \frac{SEM * 1.96 * \sqrt{2}}{mean} * 100$$

with MDC_{95}=minimal detectable change at 95% confidence interval, mean=mean of all available scores for an assessment, and SEM=standard error of measurement [39, 40]

References

39. Portney LG, Watkins MP. Foundations of clinical research: Applications to practice. 3rd ed. Upper Saddle River, N.J.: Pearson/Prentice Hall; 2015.
40. Schwenk M, Gogulla S, Englert S, Czempik A, Hauer K. Test-retest reliability and minimal detectable change of repeated sit-to-stand analysis using one body fixed sensor in geriatric patients. Physiol Meas. 2012;33:1931–46. doi:10.1088/0967-3334/33/11/1931.
