# Supplementary material for: Recommended motor assessments based on psychometric properties in individuals with dementia: a systematic review
Source: Eur Rev Aging Phys Act. 2019 Nov 3;16:20. doi: 10.1186/s11556-019-0228-z (PMC6825725; doi:10.1186/s11556-019-0228-z)
Supplement: Supplementary file 5 — Additional file 5. Study characteristics first search. [file 11556_2019_228_MOESM5_ESM.pdf]

Additional file 5

Study characteristics first search

Table S1. Study characteristics first search

Reference	Study design	Sample size	Setting	Group	Sample characteristics				Intervention
					Gender [% female]	Age [years] Mean (SD)	MMSE Mean (SD)	Aetiology of dementia	
Aguiar et al., 2014 [86]	RCT	n_r=40 n_a=34	n.r.	CG	71%	74.7 (7.4)	20.8 (4.0)	AD	Rivastigmine transdermal patch
				IG	77%	78.6 (8.4)	20.1 (4.5)		Rivastigmine transdermal patch + exercise (aerobic, flexibility, strength, balance; 2x/week, 40min)
				All	74%	n.r.	n.r., range: n.r.		6 months
Arcoverde et al., 2014 [56]	RCT	n_r=20 n_a=20	Outpatient unit	CG	50%	79.0 (n.r.)	19.9 (3.4)	AD, MD	No intervention
				IG	60%	78.5 (n.r.)	20.4 (2.7)		Treadmill walking (2x/week, 30min)
				All	n.r.	n.r.	n.r., CI_{95}: 15-25		3 months
Bossers et al., 2015 [50]	RCT	n_r=123 n_a=109	Care facility	CG	69%	85.4 (5.0)	15.9 (4.2)	AD, VD, MD, unknown	Social visits (4x/week, 30min)
				IG1	78%	85.7 (5.1)	15.8 (4.3)		Strength + walking (4x/week, 30min)
				IG2	78%	85.4 (5.4)	15.2 (4.8)		Walking (4x/week, 30min)
				All	n.r.	85.5 (5.1)	n.r., range: 9-23		9 weeks
Bossers et al., 2016 [112]	RCT	n_r=118 n_a=105	Care facility	CG	69%	85.7 (4.8)	15.9 (4.3)	AD, VD, MD, unknown	Social visits (4x/week, 30min)
				IG1	77%	85.7 (5.2)	15.9 (4.4)		Strength + walking (4x/week, 30min)
				IG2	77%	85.5 (5.4)	15.3 (4.8)		Walking (4x/week, 30min)
				All	n.r.	85.6 (5.1)	n.r., range: 9-23		9 weeks

Table S1. Study characteristics first search *(Continued)*

Reference	Study design	Sample size	Setting	Group	Sample characteristics				Intervention
					**Gender [% female]**	**Age [years] Mean (SD)**	**MMSE Mean (SD)**	**Aetiology of dementia**	
Burgener et al., 2008 [65]	RCT	n_r=43<br>n_a=33	n.r.	CG	47%	76.0 (8.1)	22.9 (5.2)	AD, VD, MD, LBD, FLD	Attention-control educational program
				IG	46%	77.9 (7.9)	24.8 (3.5)		Taiji exercise (3x/week, 60min), cognitive-behavioural therapy, support group (both bi-weekly, 90min)
				All	47%	77.0 (n.r.)	n.r., range: n.r.		20 weeks
Cancela et al., 2016 [82]	RCT	n_r=189<br>n_a=189/114*	Care facility	CG	81%	82.9 (7.4)	n.r.	n.r.	Non-physical distractive recreational activities
				IG	44%	80.6 (8.3)	n.r.		Cycling (daily, min. 15min)
				All	67%	n.r.	n.r., range: n.r.		15 months
Christofoletti et al., 2008 [66]	RCT	n_r=54<br>n_a=41	Long-term psychiatric institution	CG	70%	79.4 (2.0)	14.6 (1.2)	MD	No motor intervention
				IG1	65%	70.0 (1.8)	18.7 (1.7)		Physiotherapy, occupational therapy, physical education (5x/week, 120min)
				IG2	71%	72.9 (2.3)	12.7 (2.1)		Physiotherapy (3x/week, 60min)
				All	69%	74.3 (1.4)	n.r., range: n.r.		6 months
Cott et al., 2002 [124]	RCT	n_r=86<br>n_a=74	Care facility	CG	42%	79.8 (8.3)	6.31 (7.46)	AD	No intervention
				IG1	53%	83.2 (8.3)	6.16 (6.16)		Conversation while walking (5x/week, 30min)
				IG2	60%	81.7 (7.4)	5.44 (5.98)		Conversation (5x/week, 30min)
				All	53%	82 (8)	6 (6), range: 0-21		16 weeks
Dawson et al., 2019 [74]	RCT	n_r=23<br>n_a=23	Community-dwelling	CG	70%	74.0 (10.4)	22.0 (3.1)	n.r.	No intervention
				IG	46%	73.8 (8.5)	19.9 (6.1)		Moderate-intensity home-based functional exercise (strength, balance; 2x/week)
				All	57%	73.9 (9.1)	20.8 (5.0), range: 9-28		12 weeks

Table S1. Study characteristics first search *(Continued)*

Reference	Study design	Sample size	Setting	Group	Sample characteristics				Intervention
					Gender [% female]	Age [years] Mean (SD)	MMSE Mean (SD)	Aetiology of dementia	
Francese et al., 1997 [76]	RCT	n_r=12<br>n_a=11	Care facility	CG	n.r.	n.r.	n.r.	AD	Watching a music video sing-a-along (3x/week, 20min)
				IG	n.r.	n.r.	n.r.		Exercise (3x/week, 20min)
				All	n.r.	n.r.	n.r.		7 weeks
Hauer et al., 2017 [77]	RCT	n_r=34<br>n_a=28	Post-ward geriatric rehabilitation	CG	71%	83.3 (5.7)	18.2 (4.4)	n.r.	Usual care
				IG	65%	81.4 (6.6)	19.5 (4.6)		Postural control and strength home training (at least once per day)
				All	n.r.	81.9 (5.7)	18.8 (4.7), range: n.r.		6 weeks
Hauer et al., 2012 [78]	RCT	n_r=122<br>n_a=107	Outpatient geriatric rehabilitation	CG	73%	82.9 (7.0)	21.9 (3.2)	AD, VD, other	Motor placebo group training (2x/week, 60min)
				IG	74%	82.3 (6.6)	21.7 (2.8)		Progressive resistance and functional training (2x/week, 120min)
				All	n.r.	n.r.	n.r., range: n.r.		3 months
Henskens et al., 2018 [113]	RCT	n_r=87<br>n_a=87/ 16*	Care facility	CG1	77%	84.7 (4.6)	10.2 (5.7)	AD, VD, MD, other/ unknown	Social activity
				CG2	77%	85.1 (4.6)	12.1 (6.4)		Multicomponent aerobic and strength exercise (3x/week, 30-45min)
				IG1	91%	86.1 (5.9)	13.2 (3.7)		Activities of daily living training + social activity
				IG2	63%	87.0 (7.2)	13.6 (5.6)		Activities of daily living training + multicomponent aerobic and strength exercise (3x/week, 30-45min)
				All	n.r.	n.r.	n.r., range: n.r.		6 months

Table S1. Study characteristics first search *(Continued)*

Reference	Study design	Sample size	Setting	Group	Sample characteristics				Intervention
					Gender [% female]	Age [years] Mean (SD)	MMSE Mean (SD)	Aetiology of dementia	
Kampragkou et al., 2017 [83]	RCT	n_r=36 n_a=30	Hospitalised	CG	n.r.	n.r.	16.0 (3.0)	AD	Memory games (3x/week, 40min)
				IG	n.r.	n.r.	14.7 (3.1)		Aerobic exercise (3x/week, 30min), memory games, attention, speech, and music (3x/week, 10min)
				All	n.r.	n.r.	n.r., range: n.r.		12 weeks
Kemoun et al., 2010 [96]	RCT	n_r=38 n_a=31	Care facility	CG	73%	81.7 (5.1)	12.9	AD	No intervention
				IG	75%	82.0 (5.8)	12.6		Exercise (walking, equilibrium, stamina; 3x/week, 60min)
				All	74%	81.8 (5.3)	n.r., range: 7-20		15 weeks
Kim et al., 2016 [67]	RCT	n_r=38 n_a=31	Care facility	CG	86%	80.9 (6.1)	16.6 (4.0)	AD	Multicomponent intervention (5x/week, 2x/day, 60min)
				IG	68%	81.9 (7.0)	13.4 (4.2)		Lower-limb aerobic exercise (5x/week, 60min) + multicomponent intervention (5x/week, 2x/day, 60min)
				All	76%	81.5 (6.6)	14.8 (4.4), range: n.r.		6 months
Kovács et al., 2013 [79]	RCT	n_r=86 n_a=62	Care facility	CG	79	79.3 (12.7)	20.9 (3.8)	n.r.	Usual care including social activities
				IG	83	76.4 (9.4)	20.9 (3.2)		Exercise (strengthening, balance, walking; 2x/week)
				All	81%	n.r.	n.r., range: n.r.		12 months
Lam et al., 2018 [68]	RCT	n_r=54 n_a=54	Day-care facility	CG	78%	79.9 (6.7)	15.6 (4.5)	n.r.	Routine program including 30-60min of exercise (2x/week)
				IG	70%	79.7 (5.5)	13.6 (4.7)		Routine program including 30-60min of exercise + whole-body vibration training (4-6min + rest, 2x/week)
				All	74%	79.8 (6.1)	n.r., range: n.r.		9 weeks

Table S1. Study characteristics first search *(Continued)*

Reference	Study design	Sample size	Setting	Group	Sample characteristics				Intervention
					Gender [% female]	Age [years] Mean (SD)	MMSE Mean (SD)	Aetiology of dementia	
Miu et al., 2008 [57]	RCT	n_r=85 n_a=82	Community-dwelling	CG	63%	78 (6)	Median=20	AD, VD, MD, PD	Conventional medical treatment
				IG	43%	75 (7)	Median=20		Aerobic exercise (treadmill, bicycle, arm ergometer, flexibility exercises; 2x/week, 45-60min)
				All	54%	76 (6)	Median=20, range: n.r.		12 weeks
Netz et al., 2007 [58]	RCT	n_r=29 n_a=24	Day-care facility	CG	n.r.	n.r.	n.r.	n.r.	Social activity
				IG	n.r.	n.r.	n.r.		Group physical activity (2x/week, 45min)
				All	52%	76.9 (6.7)	13.3 (5.8), range: n.r.		12 weeks
Padala et al., 2017 [69]	RCT	n_r=30 n_a=30	Community-dwelling	CG	40%	73.9 (7.1)	22.7 (2.3)	AD	Walking (5x/week, 30min)
				IG	33%	72.1 (5.3)	23.3 (2.2)		Wii-Fit (5x/week, 30min)
				All	37%	73.0 (6.2)	22.9 (2.2), range: n.r.		8 weeks
Padala et al., 2012 [70]	RCT	n_r=22 n_a=22	Care facility	CG	73%	81.6 (5.2)	24.9 (3.6)	AD	Walking (5x/week, 30min)
				IG	73%	79.3 (9.8)	22.6 (4.3)		Wii-Fit (5x/week, 30min)
				All	73%	n.r.	n.r., range: n.r.		8 weeks
Pedrinolla et al., 2018 [93]	RCT	n_r=53 n_a=34	Community-dwelling	CG	61%	79 (6)	21 (5)	AD	Standard cognitive treatment (3x/week, 90min)
				IG	63%	80 (7)	22 (5)		Aerobic and strength training (3x/week, 90min)
				All	62%	n.r.	n.r., range: n.r.		6 months

Table S1. Study characteristics first search *(Continued)*

Reference	Study design	Sample size	Setting	Group	Sample characteristics				Intervention
					Gender [% female]	Age [years] Mean (SD)	MMSE Mean (SD)	Aetiology of dementia	
Pitkälä et al., 2013 [109]	RCT	n_r=210 n_a=194	Community-dwelling	CG	37%	78.1 (5.3)	17.7 (6.2)	AD	Usual community care
				IG1	36%	78.3 (5.1)	18.5 (6.3)		Group-based exercise (2x/week, 60min)
				IG2	43%	77.7 (5.4)	17.8 (6.6)		Home-based exercise (2x/week, 60min)
				All	39%	n.r.	n.r., range: n.r.		12 months
Pomeroy et al., 1999 [125]	RCT	n_r=81 n_a=78	Community-dwelling	CG	74%	81.8 (8.4)	n.r.	AD, MD, MID, undefined	Non-physical activities
				IG	74%	82 (8.0)	n.r.		Physiotherapy (up to 10x 30min)
				All	74%	81.9 (n.r.)	n.r., range: n.r.		2 weeks
Roach et al., 2011 [105]	RCT	n_r=105 n_a=82	Care facility	CG	n.r.	88.2 (5.8)	9.4 (7.2)	AD	Conversation (5x/week, 15-30min)
				IG1	n.r.	89.2 (6.5)	8.7 (7.8)		Exercise (strength, flexibility, balance, walking; 5x/week, 25-50min)
				IG2	n.r.	87.3 (6.1)	12.2 (7.5)		Walking (5x/week, 15-30min)
				All	n.r.	88.2 (6.1)	10.2 (7.6), range: n.r.		16 weeks
Rolland et al., 2007 [89]	RCT	n_r=134 n_a=110	Care facility	CG	79%	83.1 (7.0)	7.9 (6.4)	AD	Routine medical care
				IG	72%	82.8 (7.8)	9.7 (6.8)		Exercise (walking, strength, balance, flexibility; 2x/week, 60min)
				All	75%	83 (7.4)	8.8 (6.6), range: n.r.		12 months
Santana-Sosa et al., 2008 [80]	RCT	n_r=16 n_a=16	Care facility	CG	63%	73 (4)	19.9 (1.7)	AD	Routine nursing/medical care
				IG	63%	76 (4)	20.1 (2.3)		Training program (resistance, flexibility, joint mobility, balance/ coordination; 3x/week, 75min)
				All	63%	n.r.	n.r., range: n.r.		12 weeks

Table S1. Study characteristics first search *(Continued)*

Reference	Study design	Sample size	Setting	Group	Sample characteristics				Intervention
					Gender [% female]	Age [years] Mean (SD)	MMSE Mean (SD)	Aetiology of dementia	
Schwenk et al., 2014 [94]	Quasi RCT	n_r=148 n_a=130	Geriatric hospital	CG	76%	83.9 (6.1)	22.2 (2.3)	n.r.	Usual care treatment (occupational therapy, speech therapy, physiotherapy including exercise)
				IG	84%	84.2 (6.2)	21.4 (2.6)		Usual care treatment (see above) + intensive exercise (2x daily, up to 60min)
				All	n.r.	n.r.	n.r., range: n.r.		During rehabilitation period, average: 18.1 (6.8) days
Schwenk et al., 2014 [95]	RCT	n_r=61 n_a=49	Outpatients	CG	63%	82.3 (7.9)	21.7 (2.9)	n.r.	Low-intensity motor placebo activity program (60min, 2x/week)
				IG	65%	80.4 (7.1)	21.0 (2.9)		Progressive resistance and functional group training (120min, 2x/week)
				All	64%	81.9 (7.5)	21.4 (2.9), range: n.r.		3 months
Schwenk et al., 2010 [126]	RCT	n_r=61 n_a=49	n.r.	CG	63%	82.3 (7.9)	21.7 (2.9)	n.r.	Unspecific low-intensity exercise (60min, 2x/week)
				IG	65%	80.4 (7.1)	21.0 (2.9)		Dual-task–based exercise training (120min, 2x/week)
				All	64%	81.9 (7.5)	21.4 (2.9), range: n.r.		12 weeks
Sobol et al., 2016 [84]	RCT	n_r=200 n_a=189	Community-dwelling	CG	39%	71.3 (7.3)	24.1 (3.8)	AD	Usual care
				IG	48%	69.8 (7.4)	23.8 (3.4)		Moderate-to-high intensity aerobic exercise (3x/week, 60min)
				All	44%	70.5 (7.4)	24.0 (3.6), range: n.r.		16 weeks
Souto Barreto et al., 2017 [90]	Cluster RCT	n_r=98 n_a=91	Care facility	CG	77%	86.9 (5.8)	10.8 (5.5)	AD, VD, MD	Social activity (2x/week, 60min)
				IG	93%	88.3 (5.1)	11.4 (6.2)		Multicomponent training (range of motion, coordination, balance strength, aerobic; 2x/week, 60min)
				All	86%	n.r.	n.r., range: n.r.		24 weeks

Table S1. Study characteristics first search *(Continued)*

Reference	Study design	Sample size	Setting	Group	Sample characteristics				Intervention
					Gender [% female]	Age [years] Mean (SD)	MMSE Mean (SD)	Aetiology of dementia	
Steinberg et al., 2009 [98]	RCT	n_r=27<br>n_a=27	Community-dwelling	CG	69%	74.0 (8.1)	15.5 (5.4)	AD	Home safety assessment
				IG	71%	76.5 (3.9)	20.1 (5.1)		Home-based exercise (aerobic, strength, balance, flexibility; daily)
				All	79%	n.r.	n.r., range: n.r.		12 weeks
Suttanon et al., 2013 [52]	RCT	n_r=40<br>n_a=40	Community-dwelling	CG	57%	80.5 (6.0)	21.7 (4.4)	AD	Home-based education program
				IG	68%	83.4 (5.1)	20.9 (4.7)		Home-based individually tailored exercise (balance, strength, walking; 5x/week)
				All	62.5%	81.9 (5.7)	n.r., range: n.r.		6 months
Tappen et al., 2000 [106]	RCT	n_r=71<br>n_a=65	Care facility	CG	n.r.	89.6 (6.5)	12.5 (5.9)	AD	Conversation (3x/week, 30min)
				IG1	n.r.	84.3 (7.5)	10.8 (6.0)		Walking combined with conversation (3x/week, 30min)
				IG2	n.r.	87.4 (5.9)	9.8 (6.0)		Walking (3x/week, 30min)
				All	84%	87 (n.r.)	10.8 (n.r.), range: 0-23		16 weeks
Telenius et al., 2015 [71]	RCT	n_r=170<br>n_a=160	Care facility	CG	75%	86.4 (7.8)	15.8 (5.0)	n.r.	Leisure activities (2x/week, 50-60min)
				IG	72%	86.9 (7)	15.6 (5.0)		Intensive strengthening and balance exercises (2x/week, 50-60min)
				All	74%	86.7 (7.4)	15.7 (5.0), range: n.r.		12 weeks
Toots et al., 2017 [91]	Cluster RCT	n_r=186<br>n_a=153	Care facility	CG	76%	85.9 (7.8)	14.4 (3.5)	AD, VD, MD, other	Seated attention control activity
				IG	75%	84.4 (6.2)	15.4 (3.4)		High-intensity functional exercise (strength, balance, mobility; 2-3x/week, 45min)
				All	76%	85.1 (7.1)	14.9 (3.5), range: n.r.		4 months

Table S1. Study characteristics first search *(Continued)*

Reference	Study design	Sample size	Setting	Group	Sample characteristics				Intervention
					Gender [% female]	Age [years] Mean (SD)	MMSE Mean (SD)	Aetiology of dementia	
Toots et al., 2016 [72]	Cluster RCT	n_r=186<br>n_a=167	Care facility	CG	76%	85.9 (7.8)	14.4 (3.5)	AD, VD, MD, other	Seated attention control activity
				IG	75%	84.4 (6.2)	15.4 (3.4)		High-intensity functional exercise (strength, balance, mobility; 2-3x/week, 45min)
				All	76%	85.1 (7.1)	14.9 (3.5), range: n.r.		4 months
Toulotte et al., 2003 [85]	RCT	n_r=20<br>n_a=20	n.r.	CG	n.r.	81.9 (4.1)	18.0 (5.4)	AD, PD, stroke, unknown	Daily routine
				IG	n.r.	81.0 (5.6)	14.7 (7.6)		Physical training (2x/week, 45min)
				All	n.r.	81.4 (4.7)	16.3 (6.5), range: n.r.		16 weeks
Venturelli et al., 2011 [107]	RCT	n_r=24<br>n_a=21	Care facility	CG	n.r.	85 (5)	12 (2)	AD	Routine care
				IG	n.r.	83 (6)	13 (2)		Walking program (4x/week, 30min)
				All	n.r.	84 (5)	n.r., range: n.r.		24 weeks
Vreugdenhil et al., 2012 [59]	RCT	n_r=40<br>n_a=40	Community-dwelling	CG	75%	74.7 (n.r.)	21.0 (6.3)	AD	Usual treatment
				IG	45%	73.5 (n.r.)	22.9 (5.0)		Community-based home exercise program (daily)
				All	60%	74.1 (n.r.)	22.0 (n.r.), range 10-28		4 months
Werner et al., 2017 [100]	RCT	n_r=97<br>n_a=80	Geriatric hospital, care facility, community dwelling	CG	78%	82.5 (5.7)	21.5 (3.0)	n.r.	Low-intensity motor placebo activity (2x/week, 60min)
				IG	71%	82.6 (6.1)	22.2 (2.9)		Motor learning exercise program on compensatory sit-to-stand maneuvers (2x/week, 90min)
				All	74%	82.5 (5.9)	21.9 (2.9), range: n.r.		10weeks
Wesson et al., 2013 [61]	RCT	n_r=22<br>n_a=21	Community-dwelling	CG	36%	80.9 (5.0)	22.5 (4.3)	n.r.	Usual care
				IG	46%	78.7 (4.2)	24.5 (3.1)		Strength and balance training exercises and home hazard reduction
				All	41%	n.r.	n.r., range: 15-29		12 weeks

Table S1. Study characteristics first search *(Continued)*

Reference	Study design	Sample size	Setting	Group	Sample characteristics				Intervention
					Gender [% female]	Age [years] Mean (SD)	MMSE Mean (SD)	Aetiology of dementia	
Wiloth et al., 2018 [54]	RCT	n_r=99 n_a=84	Geriatric hospital, care facility, community dwelling	CG	74%	82.2 (5.3)	21.7 (2.9)	n.r.	Supervised motor placebo group training (2x/week, 60min)
				IG	70%	82.7 (6.2)	22.2 (2.8)		Comprehensive motor-cognitive intervention program (game-based training using Physiomat, dual-task training, motor learning exercise program) (2x/week, 90min)
				All	72%	82.9 (5.8)	22.0 (2.9), range: n.r.		10 weeks
Yoon et al., 2013 [73]	RCT	n_r=30 n_a=20	Care facility	CG	n.r.	70.1 (12.2)	18.7 (1.2)	n.r.	Conventional physical therapy (5x/week, 30min) + cognitive activity (3x/week, 20min)
				IG	n.r.	77.9 (7.5)	18.0 (1.5)		Conventional physical therapy (5x/week, 30min) + cognitive activity combined with cycling (3x/week, 20min)
				All	n.r.	n.r.	n.r., range: 16-23		12 weeks

AD: Alzheimer's disease, CG: control group, CI_{95}: 95% confidence interval, FLD: Frontal lobe dementia, IG: intervention group, LBD: Lewy body disease, MD: mixed dementia (AD+VD), MID: multi-infarct dementia, MMSE: Mini-Mental State Examination, n_a: number of analysed participants, n_r: number of randomised participants, n.r.: not reported, PD: Parkinson's disease dementia, RCT: randomised controlled trial, SD: standard deviation, VD: vascular dementia

* Intention-to-treat analysis and complete-case analysis

References

50. Bossers WJR, van der Woude LHV, Boersma F, Hortobágyi T, Scherder EJA, van Heuvelen MJG. A 9-Week Aerobic and Strength Training Program Improves Cognitive and Motor Function in Patients with Dementia: A Randomized, Controlled Trial. Am J Geriatr Psychiatry. 2015;23:1106–16. doi:10.1016/j.jagp.2014.12.191.

52. Suttanon P, Hill KD, Said CM, Williams SB, Byrne KN, LoGiudice D, et al. Feasibility, safety and preliminary evidence of the effectiveness of a home-based exercise programme for older people with Alzheimer's disease: A pilot randomized controlled trial. Clin Rehabil. 2013;27:427–38. doi:10.1177/0269215512460877.

54. Wiloth S, Werner C, Lemke NC, Bauer J, Hauer K. Motor-cognitive effects of a computerized game-based training method in people with dementia: A randomized controlled trial. Aging Ment Health. 2018;22:1124–35. doi:10.1080/13607863.2017.1348472.

56. Arcoverde C, Deslandes A, Moraes H, Almeida C, Araujo NBd, Vasques PE, et al. Treadmill training as an augmentation treatment for Alzheimer's disease: A pilot randomized controlled study. Arq Neuropsiquiatr. 2014;72:190–6. doi:10.1590/0004-282X20130231.

57. Miu D, Szeto S, Mak Y. A randomized controlled trial on the effect of exercise on physical, cognitive, and affective function in dementia subjects. Asian J Gerontol Geriatr. 2008;3:8–16.

58. Netz Y, Axelrad S, Argov E. Group physical activity for demented older adults feasibility and effectiveness. Clin Rehabil. 2007;21:977–86. doi:10.1177/0269215507078318.

59. Vreugdenhil A, Cannell J, Davies A, Razay G. A community-based exercise programme to improve functional ability in people with Alzheimer's disease: A randomized controlled trial. Scand J Caring Sci. 2012;26:12–9. doi:10.1111/j.1471-6712.2011.00895.x.

61. Wesson J, Clemson L, Brodaty H, Lord S, Taylor M, Gitlin L, Close J. A feasibility study and pilot randomised trial of a tailored prevention program to reduce falls in older people with mild dementia. BMC Geriatr. 2013;13:89. doi:10.1186/1471-2318-13-89.

65. Burgener SC, Yang Y, Gilbert R, Marsh-Yant S. The effects of a multimodal intervention on outcomes of persons with early-stage dementia. Am. J. Alzheimers Dis. Other Dement. 2008;23:382–94. doi:10.1177/1533317508317527.

66. Christofoletti G, Oliani MM, Gobbi S, Stella F, Bucken Gobbi LT, Renato Canineu P. A controlled clinical trial on the effects of motor intervention on balance and cognition in institutionalized elderly patients with dementia. Clin Rehabil. 2008;22:618–26. doi:10.1177/0269215507086239.

67. Kim M-J, Han C-W, Min K-Y, Cho C-Y, Lee C-W, Ogawa Y, et al. Physical Exercise with Multicomponent Cognitive Intervention for Older Adults with Alzheimer's Disease: A 6-Month Randomized Controlled Trial. Dement Geriatr Cogn Dis Extra. 2016;6:222–32. doi:10.1159/000446508.

68. Lam FMH, Liao LR, Kwok TCY, Pang MYC. Effects of adding whole-body vibration to routine day activity program on physical functioning in elderly with mild or moderate dementia: A randomized controlled trial. Int J Geriatr Psychiatry. 2018;33:21–30. doi:10.1002/gps.4662.

69. Padala KP, Padala PR, Lensing SY, Dennis RA, Bopp MM, Roberson PK, Sullivan DH. Home-Based Exercise Program Improves Balance and Fear of Falling in Community-Dwelling Older Adults with Mild Alzheimer's Disease: A Pilot Study. J Alzheimers Dis. 2017;59:565–74. doi:10.3233/JAD-170120.

70. Padala KP, Padala PR, Malloy TR, Geske JA, Dubbert PM, Dennis RA, et al. Wii-fit for improving gait and balance in an assisted living facility: A pilot study. J Aging Res. 2012;2012:597573. doi:10.1155/2012/597573.

71. Telenius EW, Engedal K, Bergland A. Effect of a high-intensity exercise program on physical function and mental health in nursing home residents with dementia: An assessor blinded randomized controlled trial. PLoS One. 2015;10:e0126102. doi:10.1371/journal.pone.0126102.

72. Toots A, Littbrand H, Lindelöf N, Wiklund R, Holmberg H, Nordström P, et al. Effects of a High-Intensity Functional Exercise Program on Dependence in Activities of Daily Living and Balance in Older Adults with Dementia. J Am Geriatr Soc. 2016;64:55–64. doi:10.1111/jgs.13880.

73. Yoon JE, Lee SM, Lim HS, Kim TH, Jeon JK, Mun MH. The effects of cognitive activity combined with active extremity exercise on balance, walking activity, memory level and quality of life of an older adult sample with dementia. J. Phys. Ther. Sci. 2013;25:1601–4. doi:10.1589/jpts.25.1601.

74. Dawson N, Judge KS, Gerhart H. Improved Functional Performance in Individuals With Dementia After a Moderate-Intensity Home-Based Exercise Program: A Randomized Controlled Trial. J Geriatr Phys Ther. 2019;42:18–27. doi:10.1519/JPT.0000000000000128.

76. Francese T, Sorrell J, Butler FR. The effects of regular exercise on muscle strength and functional abilities of late stage Alzheimer's residents. Am J Alzheimers Dis Other Demen. 1997;12:122–7. doi:10.1177/153331759701200305.

77. Hauer K, Ullrich P, Dutzi I, Beurskens R, Kern S, Bauer J, Schwenk M. Effects of Standardized Home Training in Patients with Cognitive Impairment following Geriatric Rehabilitation: A Randomized Controlled Pilot Study. Gerontology. 2017;63:495–506. doi:10.1159/000478263.

78. Hauer K, Schwenk M, Zieschang T, Essig M, Becker C, Oster P. Physical training improves motor performance in people with dementia: A randomized controlled trial. J Am Geriatr Soc. 2012;60:8–15. doi:10.1111/j.1532-5415.2011.03778.x.

79. Kovács E, Sztruhár Jónásné I, Karóczi CK, Korpos A, Gondos T. Effects of a multimodal exercise program on balance, functional mobility and fall risk in older adults with cognitive impairment: A randomized controlled single-blind study. Eur J Phys Rehabil Med. 2013;49:639–48.

80. Santana-Sosa E, Barriopedro MI, López-Mojares LM, Pérez M, Lucia A. Exercise training is beneficial for Alzheimer's patients. Int J Sports Med. 2008;29:845–50. doi:10.1055/s-2008-1038432.

82. Cancela JM, Ayán C, Varela S, Seijo M. Effects of a long-term aerobic exercise intervention on institutionalized patients with dementia. J Sci Med Sport. 2016;19:293–8. doi:10.1016/j.jsams.2015.05.007.

83. Kampragkou C, Iakovidis P, Kampragkou E, Kellis E. Effects of a 12-Week Aerobic Exercise Program Combined with Music Therapy and Memory Exercises on Cognitive and Functional Ability in People with Middle Type of Alzheimer's Disease. International Journal of Physiotherapy 2017. doi:10.15621/ijphy/2017/v4i5/159420.

84. Sobol NA, Hoffmann K, Frederiksen KS, Vogel A, Vestergaard K, Brændgaard H, et al. Effect of aerobic exercise on physical performance in patients with Alzheimer's disease. Alzheimers Dement. 2016;12:1207–15. doi:10.1016/j.jalz.2016.05.004.

85. Toulotte C, Fabre C, Dangremont B, Lensel G, Thévenon A. Effects of physical training on the physical capacity of frail, demented patients with a history of falling: A randomised controlled trial. Age Ageing. 2003;32:67–73. doi:10.1093/ageing/32.1.67.

86. Aguiar P, Monteiro L, Feres A, Gomes I, Melo A. Rivastigmine transdermal patch and physical exercises for Alzheimer's disease: A randomized clinical trial. Curr Alzheimer Res. 2014;11:532–7. doi:10.2174/1567205011666140618102224.

89. Rolland Y, Pillard F, Klapouszczak A, Reynish E, Thomas D, Andrieu S, et al. Exercise program for nursing home residents with Alzheimer's disease: A 1-year randomized, controlled trial. J Am Geriatr Soc. 2007;55:158–65. doi:10.1111/j.1532-5415.2007.01035.x.

90. Souto Barreto P de, Cesari M, Denormandie P, Armaingaud D, Vellas B, Rolland Y. Exercise or Social Intervention for Nursing Home Residents with Dementia: A Pilot Randomized, Controlled Trial. J Am Geriatr Soc. 2017;65:E123-E129. doi:10.1111/jgs.14947.

91. Toots A, Littbrand H, Holmberg H, Nordström P, Lundin-Olsson L, Gustafson Y, Rosendahl E. Walking Aids Moderate Exercise Effects on Gait Speed in People With Dementia: A Randomized Controlled Trial. J Am Med Dir Assoc. 2017;18:227–33. doi:10.1016/j.jamda.2016.09.003.

93. Pedrinolla A, Venturelli M, Fonte C, Munari D, Benetti MV, Rudi D, et al. Exercise Training on Locomotion in Patients with Alzheimer's Disease: A Feasibility Study. J Alzheimers Dis. 2018;61:1599–609. doi:10.3233/JAD-170625.

94. Schwenk M, Dutzi I, Englert S, Micol W, Najafi B, Mohler J, Hauer K. An intensive exercise program improves motor performances in patients with dementia: Translational model of geriatric rehabilitation. J Alzheimers Dis. 2014;39:487–98. doi:10.3233/JAD-130470.

95. Schwenk M, Zieschang T, Englert S, Grewal G, Najafi B, Hauer K. Improvements in gait characteristics after intensive resistance and functional training in people with dementia: A randomised controlled trial. BMC Geriatr. 2014;14:73. doi:10.1186/1471-2318-14-73.

96. Kemoun G, Thibaud M, Roumagne N, Carette P, Albinet C, Toussaint L, et al. Effects of a physical training programme on cognitive function and walking efficiency in elderly persons with dementia. Dement Geriatr Cogn Disord. 2010;29:109–14. doi:10.1159/000272435.

98. Steinberg M, Leoutsakos J-MS, Podewils LJ, Lyketsos CG. Evaluation of a home-based exercise program in the treatment of Alzheimer's disease: The Maximizing Independence in Dementia (MIND) study. Int J Geriatr Psychiatry. 2009;24:680–5. doi:10.1002/gps.2175.

100. Werner C, Wiloth S, Lemke NC, Kronbach F, Jansen C-P, Oster P, et al. People with Dementia Can Learn Compensatory Movement Maneuvers for the Sit-to-Stand Task: A Randomized Controlled Trial. J Alzheimers Dis. 2017;60:107–20. doi:10.3233/JAD-170258.

105. Roach KE, Tappen RM, Kirk-Sanchez N, Williams CL, Loewenstein D. A randomized controlled trial of an activity specific exercise program for individuals with Alzheimer disease in long-term care settings. J Geriatr Phys Ther. 2011;34:50–6. doi:10.1519/JPT.0b013e31820aab9c.

106. Tappen RM, Roach KE, Applegate EB, Stowell P. Effect of a combined walking and conversation intervention on functional mobility of nursing home residents with Alzheimer disease. Alzheimer Dis Assoc Disord. 2000;14:196–201. doi:10.1097/00002093-200010000-00002.

107. Venturelli M, Scarsini R, Schena F. Six-month walking program changes cognitive and ADL performance in patients with Alzheimer. Am J Alzheimers Dis Other Demen. 2011;26:381–8. doi:10.1177/1533317511418956.

109. Pitkälä KH, Pöysti MM, Laakkonen M-L, Tilvis RS, Savikko N, Kautiainen H, Strandberg TE. Effects of the Finnish Alzheimer disease exercise trial (FINALEX): A randomized controlled trial. JAMA Intern Med. 2013;173:894–901. doi:10.1001/jamainternmed.2013.359.

112. Bossers WJR, van der Woude LHV, Boersma F, Hortobágyi T, Scherder EJA, van Heuvelen MJG. Comparison of Effect of Two Exercise Programs on Activities of Daily Living in Individuals with Dementia: A 9-Week Randomized, Controlled Trial. J Am Geriatr Soc. 2016;64:1258–66. doi:10.1111/jgs.14160.

113. Henskens M, Nauta IM, Drost KT, Scherder EJ. The effects of movement stimulation on activities of daily living performance and quality of life in nursing home residents with dementia: A randomized controlled trial. Clin Interv Aging. 2018;13:805–17. doi:10.2147/CIA.S160031.

124. Cott CA, Dawson P, Sidani S, Wells D. The effects of a walking/talking program on communication, ambulation, and functional status in residents with Alzheimer disease. Alzheimer Dis Assoc Disord. 2002;16:81–7. doi:10.1097/00002093-200204000-00005.

125. Pomeroy VM, Warren CM, Honeycombe C, Briggs RS, Wilkinson DG, Pickering RM, Steiner A. Mobility and dementia: Is physiotherapy treatment during respite care effective? Int J Geriatr Psychiatry. 1999;14:389–97. doi:10.1002/(SICI)1099-1166(199905)14:5<389::AID-GPS933>3.0.CO;2-8.

126. Schwenk M, Zieschang T, Oster P, Hauer K. Dual-task performances can be improved in patients with dementia: A randomized controlled trial. Neurology. 2010;74:1961–8. doi:10.1212/WNL.0b013e3181e39696.
