# Supplementary material for: Recommended motor assessments based on psychometric properties in individuals with dementia: a systematic review
Source: Eur Rev Aging Phys Act. 2019 Nov 3;16:20. doi: 10.1186/s11556-019-0228-z (PMC6825725; doi:10.1186/s11556-019-0228-z)
Supplement: Supplementary file 6 — Additional file 6. Study characteristics main search. [file 11556_2019_228_MOESM6_ESM.pdf]

Additional file 6

Study characteristics main search

Table S2. Study characteristics main search

Reference	Sample size	Setting	Sample characteristics				Cueing	Psychometric property (time interval if applicable)	Methodology	COSMIN risk of bias	
			Gender [% female]	Age [years] Mean (SD)	MMSE Mean (SD), range	Aetiology of dementia				Relative reliability	Absolute reliability
Alencar et al., 2012 [120]	n=76	Non-institutional-ised	84%	83.9 (5.8)	12.7 (7.2), n.r.<br>SG0: n.r.<br>SG1: n.r.<br>SG2: n.r.<br>SG3: n.r.	AD, VD	(a) n.r.	Between-day TRR (1 week)	ICC (n.r.)	Adequate	N/A
Blankevoort et al., 2013 [17]	n=58	(Day-)care facilities	71%	82.5 (5.3)	19.2 (4.4), 10-28<br>SG1: 22.8 (2.1), 20-28<br>SG2: 15.5 (2.6), 10-19	n.r.	(d) verbal & visual/tactile cueing	Between-day TRR (1 week)	ICC (2-way, random, absolute agreement on single measures model) with CI_{95}<br>SEM with CI_{95}, MDC_{95}	Adequate	Adequate
Bossers et al., 2014 [63]	n=42	Care facilities	79%	86.7 (5.2)	17.1 (4.3), 9-24<br>SG1: n.r., 20-24<br>SG2: n.r., 9-19	AD, VD, MD, LBD	(b) no cueing	Between-day TRR (1 week)	ICC (2-way mixed single measure, absolute agreement model) with CI_{95}<br>SEM with CI_{95}, MDC_{95}	Adequate	Adequate

Table S2. Study characteristics main search *(Continued)*

Reference	Sample size	Setting	Sample characteristics				Cueing	Psychometric property (time interval if applicable)	Methodology	COSMIN risk of bias	
			**Gender [% female]**	**Age [years] Mean (SD)**	**MMSE Mean (SD), range**	**Aetiology of dementia**				**Relative reliability**	**Absolute reliability**
Bronas et al., 2017 [115]	n=44	Community-dwelling	45%	78.4 (6.8)	21.6 (3.3), n.r.	AD	(a) n.r./(c) verbal cueing	Simple associations	Pearson bivariate correlation	N/A	N/A
Graessel et al., 2009 [110]	n=46	Care facility	91%	85.9 (6.6)	16.0 (6.1), 1-28	n.r.	(b) no cueing	Construct validity	Spearman's rank correlation coefficient	N/A	N/A
								Internal consistency	Cronbach's α, Spearman's rank correlation coefficient	N/A	N/A
								Between-day TRR (2 weeks)	Spearman's rank correlation coefficient	Doubtful	N/A
Lee et al., 2017 [43]	n=53	Care facility	n.r.	83.8 (9.9)	13.8 (5.7), n.r.	n.r.	(e) more extensive cueing than (c) & (d) including physical assistance	IRR (3 raters)	ICC (2,3) SEM, MDC_{95}, $MDC_{\%}$	Adequate	Very good
								Between-day TRR (3-7 days)		Adequate	Adequate

Table S2. Study characteristics main search *(Continued)*

Reference	Sample size	Setting	Sample characteristics				Cueing	Psychometric property (time interval if applicable)	Methodology	COSMIN risk of bias	
			Gender [% female]	Age [years] Mean (SD)	MMSE Mean (SD), range	Aetiology of dementia				Relative reliability	Absolute reliability
Lutten-berger et al., 2012 [111]	n=139	Care facility	83%	84.7 (4.9)	15.2 (5.3), n.r.	n.r.	(b) no cueing	Construct validity	Spearman correlation coefficient	N/A	N/A
								Criterion-related validity	Correlations<br>Kruskal-Wallis test<br>Mann-Whitney U-test	N/A	N/A
								Internal consistency	Cronbach's alpha, Spearman correlation coefficient	N/A	N/A
McGough et al., 2013 [26]	n=31 (8)*	Care facility	94%	83.6 (7.0)	12.4 (7.0), 2-26	n.r.	(c) verbal cueing	Concurrent validity	Bivariate and partial correlations	N/A	N/A
								Within-day TRR (4h)	ICC (2-way, random)	Adequate	N/A
Muir-Hunter et al., 2015 [14]	n=15	Community-dwelling (day program)	27%	80.2 (5.0)	20.0 (5.5), n.r.	AD	(e) more extensive cueing than (c) & (d) including physical assistance	IRR	ICC (n.r.) with CI_{95}<br>SEM, MDC_{95}	Adequate	Very good
								Between-day TRR (1 week)		Adequate	Adequate

Table S2. Study characteristics main search *(Continued)*

Reference	Sample size	Setting	Sample characteristics				Cueing	Psychometric property (time interval if applicable)	Methodology	COSMIN risk of bias	
			Gender [% female]	Age [years] Mean (SD)	MMSE Mean (SD), range	Aetiology of dementia				Relative reliability	Absolute reliability
Ries et al., 2009 [6]	n=51	(Day-)care facility	67%	80.7 (8.8)	13.1 (8.2), n.r.<br>SG1/2: 17.4 (4.5), 10-26<br>SG3: 10.2 (8.8), n.r.	AD	(e) more extensive cueing than (c) & (d) including physical assistance	Within-day TRR (30-60 min)	ICC (2,2)/ICC (2,1)<br>SEM, MDC_{90}	Adequate	Adequate
Schwenk et al., 2014 [117]	n=77	Geriatric hospital	70%	81.8 (6.3)	22.1 (3.2), n.r.	n.r.	(a) n.r.	Criterion validity	Mann-Whitney U-test<br>Logistic regression analysis	N/A	N/A
Suttanon et al., 2011 [51]	n=14	Community dwelling	50%	79.6 (6.2)	21.4 (5.0), n.r.	AD	(d) verbal & visual/tactile cueing	Between-day TRR (1 week)	ICC (3,1)<br>SEM, MDC_{95}, CV	Adequate	Adequate
Suzuki et al., 2009 [114]	n=60	Care facility	77%	86.6 (6.2)	n.r., 0-23<br>SG1/2: n.r., 11-23<br>SG3: n.r., 0-10	AD	(a) n.r.	Known group validity	Mann-Whitney U-test/unpaired t-test	N/A	N/A
								Predictive validity	Logistic regression analysis	N/A	N/A
								Within-session TRR (3min)	ICC (n.r.)	Adequate	N/A
Suzuki et al., 2012 [116]	n=54	Care facility	76%	87.0 (5.7)	n.r., 0-24	AD, MD	(a) n.r.	Predictive validity	Chi^2 test/Mann-Whitney U-test/ unpaired t-test<br>Forward stepwise logistic regression analysis	N/A	N/A

Table S2. Study characteristics main search *(Continued)*

Reference	Sample size	Setting	Sample characteristics				Cueing	Psychometric property (time interval if applicable)	Methodology	COSMIN risk of bias	
			Gender [% female]	Age [years] Mean (SD)	MMSE Mean (SD), range	Aetiology of dementia				Relative reliability	Absolute reliability
Tappen et al., 1997 [118]	n=33	Care facility	64%	84.7 (3.9)	9.3 (6.0), 0-24	AD	(e) more extensive cueing than (c) & (d) including physical assistance	IRR	ICC (2,1)	Adequate	N/A
								Between-day and within-day TRR (1 week, AM/ PM)	ICC (3,1)	Adequate	N/A
Telenius et al., 2015 [15]	n=33	Care facility	76%	82.7 (7.2)	15.8 (5.4), n.r.	n.r.	(e) more extensive cueing than (c) & (d) including physical assistance	Internal consistency	Cronbach's α, item-to-total correlation	N/A	N/A
								IRR	Weighted κ<br>ICC model 2.1<br>SEM, MDC_{95}, $MDC_{\%}$	Adequate	Very good
Thomas et al., 2002 [102]	n=12	Day-care facility	100%	80.5 (6.2)	16.9 (7.3), n.r.	Various	(d) verbal & visual/tactile cueing	Between-day TRR (7 days)	ICC (n.r.)	Adequate	N/A

Table S2. Study characteristics main search *(Continued)*

Reference	Sample size	Setting	Sample characteristics				Cueing	Psychometric property (time interval if applicable)	Methodology	COSMIN risk of bias	
			Gender [% female]	Age [years] Mean (SD)	MMSE Mean (SD), range	Aetiology of dementia				Relative reliability	Absolute reliability
Werner et al., 2018 [99]	n=97	Geriatric hospital, care facility, community-dwelling	74%	82.5 (5.9)	21.0 (2.9), n.r.	n.r.	(b) no cueing	Concurrent validity	Point-biserial correlation coefficients	N/A	N/A
								Intra-rater reliability (repeated baseline scoring 4 weeks later)	Percentage agreement, Cohen's κ<br>ICC (3,1) with CI_{95}	Very good	N/A
								IRR	Percentage agreement, Cohen's κ<br>ICC (2,1) with CI_{95}	Very good	N/A
Wiloth et al., 2016 [53]	n=105 (74)*	Geriatric hospital, care facility, community dwelling	72%	82.7 (5.9)	21.9 (2.8), n.r.	n.r.	(b) no cueing	Construct validity	Spearman's rank correlations	N/A	N/A
								Between-day TRR (2-5 days)	Spearman's rank correlations<br>ICC (2-way mixed model) with CI_{95}	Adequate	N/A
Wittwer et al., 2008 [121]	n=20	Community-dwelling	50%	80.6 (5.2)	22.0 (3.5), 13-27	AD	(b) no cueing	Between-day TRR (1 week)	ICC (3,1) with CI_{95}<br>MDC_{95}, CV	Adequate	Adequate

Table S2. Study characteristics main search *(Continued)*

Reference	Sample size	Setting	Sample characteristics				Cueing	Psychometric property (time interval if applicable)	Methodology	COSMIN risk of bias	
			Gender [% female]	Age [years] Mean (SD)	MMSE Mean (SD), range	Aetiology of dementia				Relative reliability	Absolute reliability
Wittwer et al., 2013 [5]	n=16	Community-dwelling	63%	81.1 (5.2)	21.4 (4.0), 13-26	AD	(a) n.r.	Between-day TRR (1 week)	ICC (3,1) with CI_{95}<br>SEM, MDC_{95}	Adequate	Adequate

AD: Alzheimer's disease, AM/PM: morning/afternoon measures, CI_{95}: 95% confidence interval, COSMIN: COnsensus-based Standards for the selection of health Measurement INstruments, CV: coefficient of variation, ICC: intraclass correlation coefficient, IRR: inter-rater reliability, LBD: Lewy body disease, MD: mixed dementia (AD+VD), MDC: minimal detectable change with MDC_{95}=SEM*1.96*Sqrt(2), MDC_{90}=SEM*1.65*Sqrt(2), $MDC_{\%}$=(MDC_{95} / mean)*100, MMSE: Mini-Mental State Examination, n: number of participants, N/A: not applicable, n.r.: not reported, SD: standard deviation, SEM: standard error of measurement with SEM=SD*Sqrt(1-ICC), SEM=SD*Sqrt(1-r), SG0: subgroup borderline, SG1: subgroup mild dementia, SG1/2: subgroup mild to moderate dementia, SG2: subgroup moderate dementia, SG3: subgroup severe dementia, TRR: test-retest reliability, VD: vascular dementia

* Reliability was assessed in a subgroup

References

5. Wittwer JE, Webster KE, Hill K. Reproducibility of gait variability measures in people with Alzheimer's disease. Gait Posture. 2013;38:507–10. doi:10.1016/j.gaitpost.2013.01.021.

6. Ries JD, Echternach JL, Nof L, Gagnon Blodgett M. Test-retest reliability and minimal detectable change scores for the timed "up & go" test, the six-minute walk test, and gait speed in people with Alzheimer disease. Phys Ther. 2009;89:569–79. doi:10.2522/ptj.20080258.

14. Muir-Hunter SW, Graham L, Montero Odasso M. Reliability of the Berg Balance Scale as a Clinical Measure of Balance in Community-Dwelling Older Adults with Mild to Moderate Alzheimer Disease: A Pilot Study. Physiother. Can. 2015;67:255–62. doi:10.3138/ptc.2014-32.

15. Telenius EW, Engedal K, Bergland A. Inter-rater reliability of the Berg Balance Scale, 30 s chair stand test and 6 m walking test, and construct validity of the Berg Balance Scale in nursing home residents with mild-to-moderate dementia. BMJ Open. 2015;5:e008321. doi:10.1136/bmjopen-2015-008321.

17. Blankevoort CG, van Heuvelen MJG, Scherder EJA. Reliability of six physical performance tests in older people with dementia. Phys Ther. 2013;93:69–78. doi:10.2522/ptj.20110164.

26. McGough EL, Logsdon RG, Kelly VE, Teri L. Functional mobility limitations and falls in assisted living residents with dementia: Physical performance assessment and quantitative gait analysis. J Geriatr Phys Ther. 2013;36:78–86. doi:10.1519/JPT.0b013e318268de7f.

43. Lee HS, Park SW, Chung HK. The Korean version of relative and absolute reliability of gait and balance assessment tools for patients with dementia in day care center and nursing home. J. Phys. Ther. Sci. 2017;29:1934–9. doi:10.1589/jpts.29.1934.

51. Suttanon P, Hill KD, Dodd KJ, Said CM. Retest reliability of balance and mobility measurements in people with mild to moderate Alzheimer's disease. Int Psychogeriatr. 2011;23:1152–9. doi:10.1017/S1041610211000639.

53. Wiloth S, Lemke N, Werner C, Hauer K. Validation of a Computerized, Game-based Assessment Strategy to Measure Training Effects on Motor-Cognitive Functions in People With Dementia. JMIR Serious Games. 2016;4:e12. doi:10.2196/games.5696.

63. Bossers WJR, van der Woude LHV, Boersma F, Scherder EJA, van Heuvelen MJG. The Groningen Meander Walking Test: A dynamic walking test for older adults with dementia. Phys Ther. 2014;94:262–72. doi:10.2522/ptj.20130077.

99. Werner C, Wiloth S, Lemke NC, Kronbach F, Hauer K. Development and Validation of a Novel Motor-Cognitive Assessment Strategy of Compensatory Sit-to-Stand Maneuvers in People With Dementia. J Geriatr Phys Ther. 2018;41:143–54. doi:10.1519/JPT.0000000000000116.

102. Thomas VS, Hageman PA. A preliminary study on the reliability of physical performance measures in older day-care center clients with dementia. Int. Psychogeriatr. 2002;14:17–23. doi:10.1017/S1041610202008244.

110. Graessel E, Viegas R, Stemmer R, Küchly B, Kornhuber J, Donath C. The Erlangen Test of Activities of Daily Living: First results on reliability and validity of a short performance test to measure fundamental activities of daily living in dementia patients. Int. Psychogeriatr. 2009;21:103–12. doi:10.1017/S1041610208007710.

111. Luttenberger K, Schmiedeberg A, Gräßel E. Activities of daily living in dementia: Revalidation of the E-ADL Test and suggestions for further development. BMC Psychiatry. 2012;12:208. doi:10.1186/1471-244X-12-208.

114. Suzuki M, Yamada S, Inamura A, Omori Y, Kirimoto H, Sugimura S, Miyamoto M. Reliability and validity of measurements of knee extension strength obtained from nursing home residents with dementia. Am J Phys Med Rehabil. 2009;88:924–33. doi:10.1097/PHM.0b013e3181ae1003.

115. Bronas UG, Salisbury D, Kelly K, Leon A, Chow L, Yu F. Determination of Aerobic Capacity via Cycle Ergometer Exercise Testing in Alzheimer's Disease. Am J Alzheimers Dis Other Demen. 2017;32:500–8. doi:10.1177/1533317517720065.

116. Suzuki M, Kirimoto H, Inamura A, Yagi M, Omori Y, Yamada S. The relationship between knee extension strength and lower extremity functions in nursing home residents with dementia. Disabil. Rehabil. 2012;34:202–9. doi:10.3109/09638288.2011.593678.

117. Schwenk M, Hauer K, Zieschang T, Englert S, Mohler J, Najafi B. Sensor-derived physical activity parameters can predict future falls in people with dementia. Gerontology. 2014;60:483–92. doi:10.1159/000363136.

118. Tappen RM, Roach KE, Buchner D, Barry C, Edelstein J. Reliability of Physical Performance Measures in Nursing Home Residents With Alzheimer's Disease. J. Gerontol. Ser. A-Biol. Sci. Med. Sci. 1997;52A:M52-M55. doi:10.1093/gerona/52A.1.M52.

120. Alencar MA, Dias JMD, Figueiredo LC, Dias RC. Handgrip strength in elderly with dementia: Study of reliability. Rev Bras Fisioter. 2012;16:510–4. doi:10.1590/S1413-35552012005000059.

121. Wittwer JE, Webster KE, Andrews PT, Menz HB. Test-retest reliability of spatial and temporal gait parameters of people with Alzheimer's disease. Gait Posture. 2008;28:392–6. doi:10.1016/j.gaitpost.2008.01.007.
