# Supplementary material for: Recommended motor assessments based on psychometric properties in individuals with dementia: a systematic review
Source: Eur Rev Aging Phys Act. 2019 Nov 3;16:20. doi: 10.1186/s11556-019-0228-z (PMC6825725; doi:10.1186/s11556-019-0228-z)
Supplement: Supplementary file 7 — Additional file 7. Data extraction first search. [file 11556_2019_228_MOESM7_ESM.pdf]

Additional file 7

Data extraction first search

Table S3. Data extraction first search

Reference	Motor assessments		CG Baseline Mean (SD)	CG Post Mean (SD)	CG Difference Mean (SD)	IG Baseline Mean (SD)	IG Post Mean (SD)	IG Difference Mean (SD)	Statistics / p-value	Effect size reported	Effect size calculated [cc]
Aguiar et al., 2014 [86]	TUG [s]		14.5 (4.9)	12.9 (4.2)	-1.6 (2.4)	15.0 (5.2)	15.0 (5.6)	0.0 (1.7)	p=0.062 [a]	n.r.	0.79
Arcoverde et al., 2014 [56]	FR [cm]		19.0 (4.2)	18.3 (n.r.)	-0.7 (4.3)	20.0 (4.3)	25.1 (n.r.)	5.1 (2.9)	p=0.00 [a]	1.48 [y]	1.67
	BBS		n.r. (n.r.)	n.r. (n.r.)	n.r.	n.r. (n.r.)	n.r. (n.r.)	n.r.	p=0.00 [b]	1.04 [y]	N/A
	TUG [m/s]		n.r. (n.r.)	n.r. (n.r.)	n.r. (n.r.)	n.r. (n.r.)	n.r. (n.r.)	n.r. (n.r.)	p=0.00 [b]	1.58 [y]	N/A
	Cognitive TUG [m/s]		n.r. (n.r.)	n.r. (n.r.)	n.r. (n.r.)	n.r. (n.r.)	n.r. (n.r.)	n.r. (n.r.)	p=0.24 [a]	1.03 [y]	N/A
	30s CST		9.0 (3.0)	8.5 (n.r.)	-0.5 (0.9)	9.0 (2.0)	10.0 (n.r.)	1.0 (2.4)	p=0.08 [a]	0.50 [y]	0.87
Bossers et al., 2015 [50]	FICSIT-4		2.3 (1.4)	2.0 (1.4)	n.r.	IG1: 2.3 (1.0)<br>IG2: 2.8 (1.2)	IG1: 2.8 (0.9)<br>IG2: 2.5 (1.1)	n.r.	F(2,105)=5.36, p=0.024 [c]	CG vs. IG1: 0.30 [y]<br>CG vs. IG2: 0.08 [y]<br>IG1 vs. IG2: 0.33 [y]	N/A
	Figure of Eight Test	walking speed [m/s]	0.4 (0.3)	0.3 (0.3)	n.r.	IG1: 0.3 (0.2)<br>IG2: 0.4 (0.3)	IG1: 0.4 (0.3)<br>IG2: 0.4 (0.4)	n.r.			N/A
		oversteps	9.0 (8.0)	9.7 (8.2)	n.r.	IG1: 7.7 (7.6)<br>IG2: 5.9 (7.4)	IG1: 7.3 (7.5)<br>IG2: 7.9 (7.7)	n.r.			N/A
	GMWT	time [s]	21.5 (12.7)	21.2 (13.7)	n.r.	IG1: 23.3 (12.7)<br>IG2: 18.7 (7.7)	IG1: 21.6 (11.4)<br>IG2: 19.0 (8.9)	n.r.			N/A
		oversteps	2.6 (2.9)	2.7 (2.7)	n.r.	IG1: 2.1 (2.1)<br>IG2: 0.8 (1.6)	IG1: 1.7 (2.4)<br>IG2: 1.1 (1.6)	n.r.			N/A

Table S3. Data extraction first search *(Continued)*

Refer-ence	Motor assessments		CG			IG			Statistics / p-value	Effect size	
			Baseline Mean (SD)	Post Mean (SD)	Difference Mean (SD)	Baseline Mean (SD)	Post Mean (SD)	Difference Mean (SD)		reported	calcu-lated [cc]
Bossers et al., 2015 [50]	TUG [s]		27.6 (18.8)	27.7 (19.2)	n.r.	IG1: 23.0 (13.0)<br>IG2: 24.3 (14.0)	IG1: 20.4 (9.2)<br>IG2: 23.8 (15.0)	n.r.	F(2,105)=1.28, p=0.282 [c]	CG vs. IG1: 0.28 [y]<br>CG vs. IG2: 0.06 [y]<br>IG1 vs. IG2: 0.26 [y]	N/A
	6m WT	walking speed [m/s]	0.7 (0.3)	0.6 (0.3)	n.r.	IG1: 0.7 (0.3)<br>IG2: 0.8 (0.3)	IG1: 0.8 (0.3)<br>IG2: 0.7 (0.4)	n.r.			N/A
		step length [m]	0.4 (0.1)	0.4 (0.1)	n.r.	IG1: 0.4 (0.1)<br>IG2: 0.4 (0.1)	IG1: 0.4 (0.1)<br>IG2: 0.4 (0.1)	n.r.			N/A
	Modified 30s CST		6.2 (4.8)	5.4 (6.3)	n.r.	IG1: 6.8 (3.4)<br>IG2: 7.1 (4.4)	IG1: 8.2 (3.6)<br>IG2: 6.3 (4.8)	n.r.	F(2,105)=7.07, p=0.004 [c]	CG vs. IG1: 0.38 [y]<br>CG vs. IG2: 0.04 [y]<br>IG1 vs. IG2: 0.36 [y]	N/A
	Maximum knee extension strength (dynamometer) [N]		218.9 (84.1)	203.2 (74.0)	n.r.	IG1: 205.9 (91.0)<br>IG2: 196.0 (99.0)	IG1: 208.8 (85.6)<br>IG2: 186.1 (84.3)	n.r.			N/A
	6min WT [m]		229.5 (136.4)	221.8 (159.5)	n.r.	IG1: 217.6 (90.3)<br>IG2: 231.5 (136.4)	IG1: 267.2 (101.2)<br>IG2: 235.5 (148.7)	n.r.	F(2,105)=4.53, p<0.049 [c]	CG vs. IG1: 0.47 [y]<br>CG vs. IG2: 0.08 [y]<br>IG1 vs. IG2: 0.38 [y]	N/A

Table S3. Data extraction first search *(Continued)*

Reference	Motor assessments		CG Baseline Mean (SD)	CG Post Mean (SD)	CG Difference Mean (SD)	IG Baseline Mean (SD)	IG Post Mean (SD)	IG Difference Mean (SD)	Statistics / p-value	Effect size reported	Effect size calculated [cc]
Bossers et al., 2016 [112]	Physical Performance Test		12.9 (5.9)	11.2 (6.4)	n.r.	IG1: 12.9 (3.4)<br>IG2: 13.4 (5.1)	IG1: 14.2 (3.5)<br>IG2: 13.2 (4.9)	n.r.	Chi²(2)=11.93, p=0.003 [d]	CG vs. IG1: 0.62 [y]<br>CG vs. IG2: 0.29 [y]<br>IG1 vs. IG2: 0.36 [y]	N/A
	E-ADL Test		26.9 (3.4)	25.3 (5.1)	n.r.	IG1: 26.5 (3.7)<br>IG2: 26.8 (3.5)	IG1: 28.1 (2.7)<br>IG2: 27.3 (3.8)	n.r.	Chi²(2)=16.40, p<0.001 [d]	CG vs. IG1: 0.85 [y]<br>CG vs. IG2: 0.53 [y]<br>IG1 vs. IG2: 0.31 [y]	N/A
Burgener et al., 2008 [65]	Single leg stance [s]	left leg, eyes closed	1.7 (1.1)	3.6 (4.8)	1.9 (n.r.)	3.4 (6.8)	6.3 (14.2)	2.9 (n.r.)	IG: n.s. [e]<br>p=0.62 [f]	n.r.	N/A
		right leg, eyes open	6.0 (5.5)	3.7 (2.1)	-2.3 (n.r.)	5.9 (5.6)	10.4 (15.5)	4.5 (n.r.)	IG: n.s. [e]<br>p=0.09 [f]	n.r.	N/A
	BBS		50.8 (4.2)	50.5 (3.5)	-0.3 (n.r.)	49.1 (5.0)	50.8 (4.3)	1.7 (n.r.)	p=0.87 [f]	n.r.	N/A
Cancela et al., 2016 [82]	TUG [s]		23.4 (6.9)	n.r.	-0.6 (n.r.)<br>-1.8 (n.r.) *	24.0 (10.1)	n.r.	-2.1 (n.r.)<br>-3.0 (n.r.) *	F(1,187)=5.43, p=0.03 [g]<br>F(1,111)=4.10, p=0.04 [h] *	n.r.	0.35<br>0.38 *
Christofoletti et al., 2008 [66]	BBS		35.2 (2.6)	27.4 (3.2)	n.r.	IG1: 39.5 (1.9)<br>IG2: 37.4 (2.0)	IG1: 41.7 (2.4)<br>IG2: 37.7 (2.8)	n.r.	CG vs. IG1: F=10.3, p<0.05 [i]<br>CG vs. IG2: F=7.9, p<0.05 [i]	n.r.	N/A

Table S3. Data extraction first search *(Continued)*

Reference	Motor assessments		CG			IG			Statistics / p-value	Effect size	
			Baseline Mean (SD)	Post Mean (SD)	Difference Mean (SD)	Baseline Mean (SD)	Post Mean (SD)	Difference Mean (SD)		reported	calculated [cc]
Christofoletti et al., 2008 [66]	TUG	time [s]	30.6 (6.5)	35.6 (8.6)	n.r.	IG1: 13.7 (1.2)<br>IG2: 22.3 (4.4)	IG1: 12.9 (1.0)<br>IG2: 22.1 (4.0)	n.r.	n.s. [i]	n.r.	N/A
		steps	31.3 (4.2)	35.3 (6.4)	n.r.	IG1: 19.9 (1.4)<br>IG2: 28.2 (3.6)	IG1: 18.3 (1.2)<br>IG2: 25.5 (3.6)	n.r.	n.s. [i]	n.r.	N/A
Cott et al., 2002 [124]	2-min walk test [m]		48.0 (28.8)	47.7 (33.8)	n.r.	IG1: 52.8 (27.6)<br>IG2: 52.6 (24.2)	IG1: 53.3 (27.5)<br>IG2: 56.4 (34.4)	n.r.	n.s. [e, j]	n.r.	N/A
Dawson et al., 2019 [74]	Modified BBS		38.5 (8.0)	36.6 (8.7)	n.r.	39.5 (3.3)	41.5 (2.2)	n.r.	B=4.0, β=0.3, t=4.1, p=0.001 [k]	n.r.	N/A
	8-foot walk test [m/s]	comfortable pace	0.7 (0.2)	0.6 (0.3)	n.r.	0.7 (0.2)	0.7 (0.1)	n.r.	B=0.01, β=0.2, t=0.6, p=0.6 [k]	n.r.	N/A
		fast pace	1.4 (0.6)	1.3 (0.6)	n.r.	1.2 (0.3)	1.6 (0.3)	n.r.	B=0.3, β=0.4, t=2.6, p=0.02 [k]	n.r.	N/A
	30s CST		15.7 (6.1)	13.2 (4.9)	n.r.	14.0 (5.8)	17.9 (6.8)	n.r.	B=5.9, β=0.5, t=3.3, p=0.004 [k]	n.r.	N/A
Francese et al., 1997 [76]	POMA		1.8 (2.1)	0.4 (0.9)	n.r.	3.0 (2.8)	8.7 (4.3)	n.r.	t(10)=2.00, p<0.05 [f]<br>CG: t(4)=-1.00, p≥.05 [e]<br>IG: t(5)=3.00, p=0.05 [e]	n.r.	N/A
	Physical therapy assessment		38.8 (34.7)	43.6 (37.7)	n.r.	63.8 (18.3)	89.7 (10.0)	n.r.	t(10)=3.20, p=0.01 [f]<br>CG: t(4)=0.83, p≥0.05 [e]<br>IG: t(5)=4.33, p=0.01 [e]	n.r.	N/A

Table S3. Data extraction first search *(Continued)*

Reference	Motor assessments		CG Baseline Mean (SD)	CG Post Mean (SD)	CG Difference Mean (SD)	IG Baseline Mean (SD)	IG Post Mean (SD)	IG Difference Mean (SD)	Statistics / p-value	Effect size reported	Effect size calculated [cc]
Hauer et al., 2017 [77]	POMA	total score	18.4 (7.0)	18.6 (5.2)	n.r.	18.6 (7.5)	23.0 (4.4)	n.r.	p=0.006 [i]	0.25 [z]	N/A
		balance score	11.5 (3.4)	11.7 (1.8)	n.r.	11.0 (3.4)	13.3 (1.7)	n.r.	p=0.034 [i]	0.16 [z]	N/A
		gait score	6.9 (3.9)	6.9 (3.9)	n.r.	7.6 (4.5)	9.7 (2.8)	n.r.	p=0.019 [i]	0.19 [z]	N/A
	Body-fixed-sensor-based STS analysis (DynaPort): sit-to-stand performance	duration [s]	2.0 (0.6)	2.5 (0.9)	n.r.	1.8 (0.4)	1.4 (0.2)	n.r.	p=0.064 [i]	0.30 [z]	N/A
		hip flexion, duration [s]	1.0 (0.4)	1.1 (0.4)	n.r.	0.9 (0.2)	0.8 (0.2)	n.r.	p=0.451 [i]	0.06 [z]	N/A
		hip extension, duration [s]	0.9 (0.2)	1.4 (0.6)	n.r.	0.9 (0.3)	0.6 (0.1)	n.r.	p=0.018 [i]	0.44 [z]	N/A
		hip flexion, max. angular velocity [°/s]	74.0 (21.1)	60.4 (10.0)	n.r.	86.2 (35.9)	106.0 (54.5)	n.r.	p=0.239 [i]	0.14 [z]	N/A
		hip extension, max. angular velocity [°/s]	46.9 (26.5)	41.4 (15.4)	n.r.	42.2 (22.7)	37.7 (17.5)	n.r.	p=0.919 [i]	0.001 [z]	N/A
	Body-fixed-sensor-based STS analysis (DynaPort): stand-to-sit performance	duration [s]	2.0 (0.8)	2.4 (0.7)	n.r.	2.2 (0.5)	1.5 (0.3)	n.r.	p=0.014 [i]	0.47 [z]	N/A
		hip flexion, duration [s]	1.0 (0.4)	1.3 (0.3)	n.r.	1.1 (0.3)	0.7 (0.2)	n.r.	p=0.015 [i]	0.46 [z]	N/A
		hip extension, duration [s]	0.9 (0.4)	1.1 (0.4)	n.r.	1.0 (0.2)	0.8 (0.2)	n.r.	p=0.044 [i]	0.35 [z]	N/A
		hip flexion, max. angular velocity [°/s]	36.8 (17.7)	31.5 (12.4)	n.r.	41.1 (13.8)	46.5 (27.1)	n.r.	p=0.369 [i]	0.08 [z]	N/A

Table S3. Data extraction first search *(Continued)*

Reference	Motor assessments		CG Baseline Mean (SD)	CG Post Mean (SD)	CG Difference Mean (SD)	IG Baseline Mean (SD)	IG Post Mean (SD)	IG Difference Mean (SD)	Statistics / p-value	Effect size reported	Effect size calculated [cc]
Hauer et al., 2017 [77]	Body-fixed-sensor-based STS analysis (DynaPort): stand-to-sit performance	hip extension, max. angular velocity [°/s]	81.8 (39.0)	52.5 (10.3)	n.r.	75.3 (22.6)	107.7 (50.1)	n.r.	p=0.006 [i]	0.55 [z]	N/A
	SPPB	total score	4.4 (2.9)	4.7 (2.4)	n.r.	5.0 (2.7)	7.0 (2.7)	n.r.	p=0.010 [i]	0.23 [z]	N/A
		chair rise score	0.7 (1.3)	0.7 (1.1)	n.r.	0.6 (0.8)	1.5 (1.4)	n.r.	p=0.007 [i]	0.25 [z]	N/A
		balance score	2.6 (.7)	2.7 (.7)	n.r.	2.9 (.9)	3.6 (0.7)	n.r.	p=0.066 [i]	0.12 [z]	N/A
		gait score	1.1 (1.4)	1.3 (1.1)	n.r.	1.4 (1.3)	1.9 (1.3)	n.r.	p=0.395 [i]	0.03 [z]	N/A
		8-foot walk test [m/s]	0.4 (0.2)	0.4 (0.2)	n.r.	0.5 (0.2)	0.6 (0.2)	n.r.	p=0.153 [i]	0.12 [z]	N/A
		5x STS [s]	19.1 (9.8)	24.4 (12.0)	n.r.	20.9 (5.7)	16.0 (4.0)	n.r.	p=0.009 [i]	0.45 [z]	N/A
Hauer et al., 2012 [78]	POMA	total score	19.8 (5.4)	20.6 (6.0)	n.r.	20.1 (4.8)	24.5 (3.7)	n.r.	p<0.001 [c]	0.22 [aa]	N/A
		balance score	10.8 (3.1)	11.1 (3.2)	n.r.	10.8 (2.8)	13.2 (1.9)	n.r.	p<0.001 [c]	0.23 [aa]	N/A
		gait score	9.0 (2.7)	9.4 (3.1)	n.r.	9.3 (2.3)	11.4 (1.9)	n.r.	p<0.001 [c]	0.19 [aa]	N/A
	TUG [s]		17.9 (16.0)	17.5 (17.3)	n.r.	14.9 (6.7)	11.2 (4.5)	n.r.	p=0.009 [c]	0.06 [aa]	N/A
	Handgrip dynamometer [KPa]		59.7 (16.6)	59.7 (15.7)	n.r.	59.1 (17.8)	60.9 (17.4)	n.r.	p=0.55 [c]	0.004 [aa]	N/A
	One-repetition maximum in leg press [kg]		140.9 (44.0)	136.5 (45.4)	n.r.	148.7 (57.9)	225.2 (79.7)	n.r.	p<0.001 [c]	0.43 [aa]	N/A

Table S3. Data extraction first search *(Continued)*

Reference	Motor assessments		CG Baseline Mean (SD)	CG Post Mean (SD)	CG Difference Mean (SD)	IG Baseline Mean (SD)	IG Post Mean (SD)	IG Difference Mean (SD)	Statistics / p-value	Effect size reported	Effect size calculated [cc]
Hauer et al., 2012 [78]	Maximum isometric strength (dynamometer)	knee extension [N]	65.8 (24.8)	66.8 (25.5)	n.r.	68.3 (27.3)	81.3 (27.4)	n.r.	p<0.001 [c]	0.12 [aa]	N/A
		knee extension [Ns]	267.7 (101.9)	270.5 (103.0)	n.r.	277.2 (114.4)	324.6 (122.6)	n.r.	p=0.001 [c]	0.09 [aa]	N/A
		knee flexion [N]	31.4 (12.1)	34.9 (12.9)	n.r.	33.3 (12.6)	43.7 (14.5)	n.r.	p<0.001 [c]	0.15 [aa]	N/A
		knee flexion [Ns]	137.2 (52.7)	143.5 (54.9)	n.r.	145.1 (55.3)	178.2 (61.3)	n.r.	p<0.001 [c]	0.14 [aa]	N/A
		ankle flexion [N]	52.3 (21.8)	52.5 (23.7)	n.r.	56.5 (24.7)	63.1 (26.1)	n.r.	p=0.01 [c]	0.06 [aa]	N/A
		ankle flexion [Ns]	212.6 (93.2)	214.3 (99.0)	n.r.	229.0 (100.3)	257.1 (107.5)	n.r.	p=0.01 [c]	0.06 [aa]	N/A
	Gait performance	walking sped [m/s]	0.9 (0.3)	1.0 (0.3)	n.r.	0.9 (0.3)	1.2 (0.4)	n.r.	p<0.001 [c]	0.28 [aa]	N/A
		step length [m]	0.5 (0.1)	0.5 (0.1)	n.r.	0.5 (0.2)	0.6 (0.1)	n.r.	p<0.001 [c]	0.16 [aa]	N/A
		cadence [steps/min]	116.7 (18.9)	117.9 (20.7)	n.r.	117.1 (18.7)	131.7 (17.1)	n.r.	p<0.001 [c]	0.18 [aa]	N/A
	5x STS [s]		17.6 (9.3)	19.7 (15.9)	n.r.	17.3 (6.8)	11.8 (3.2)	n.r.	p<0.001 [c]	0.15 [aa]	N/A
	Stair-climbing performance [s]		16.0 (11.0)	14.7 (12.4)	n.r.	13.3 (6.6)	9.8 (4.0)	n.r.	p=0.006 [c]	0.07 [aa]	N/A
Henskens et al., 2018 [113]	E-ADL Test		n.r.	n.r.	n.r.	n.r.	n.r.	n.r.	n.r.	n.r.	N/A
Kamprag-kou et al., 2017 [83]	One Leg Standing Balance Test [s]		4.0 (2.2)	3.5 (2.2)	n.r.	4.4 (3.1)	5.7 (3.0)	n.r.	F(1,26)=39.03, p<0.05 [i] CG: p>0.05 [l] IG: p=0.0001 [l]	n.r.	2.36

Table S3. Data extraction first search *(Continued)*

Reference	Motor assessments		CG Baseline Mean (SD)	CG Post Mean (SD)	CG Difference Mean (SD)	IG Baseline Mean (SD)	IG Post Mean (SD)	IG Difference Mean (SD)	Statistics / p-value	Effect size reported	Effect size calculated [cc]
Kamprag-kou et al., 2017 [83]	TUG [s]		19.7 (4.4)	21.1 (4.8)	n.r.	19.3 (5.0)	18.2 (4.2)	n.r.	F(1,26)=22.09, p<0.05 [i]<br>CG: p>0.05 [n]<br>IG: p>0.05 [n]	n.r.	1.78
Kemoun et al., 2010 [96]	Gait analysis	walking speed [m/s]	0.9 (0.2)	0.8 (0.2)	n.r.	0.7 (0.1)	1.0 (0.2)	n.r.	F(1,29)=53.4, p=0.01 [i]	n.r.	2.72
		stride length [m]	1.0 (0.2)	0.9 (0.2)	n.r.	0.9 (0.2)	1.0 (0.2)	n.r.	F(1,29)=16.3, p=0.01 [i]	n.r.	1.50
		double limb support time [s]	0.1 (0.0)	0.1 (0.0)	n.r.	0.2 (0.0)	0.1 (0.0)	n.r.	F(1,29)=27.0, p=0.01 [i]	n.r.	1.93
Kim et al., 2016 [67]	BBS		n.r.	n.r.	n.r.	28.2 (17.6)	21.5 (17.3)	n.r.	IG: p=0.04 [m]	n.r.	N/A
	Handgrip dynamometer [kg]		n.r.	n.r.	n.r.	7.9 (5.9)	11.8 (7.7)	n.r.	IG: p=0.02 [m]	n.r.	N/A
	Pedal Power	pedal rotation	n.r.	n.r.	n.r.	97.7 (89.9)	285.8 (197.5)	n.r.	IG: p=0.004 [m]	n.r.	N/A
		total load [W*number of pedal rotation/s]	n.r.	n.r.	n.r.	6.3 (7.5)	10.0 (6.8)	n.r.	IG: p=0.06 [m]	n.r.	N/A
Kovács et al., 2013 [79]	POMA	total score	10 (n.r.)	11 (n.r.)	n.r.	14 (n.r.)	17 (n.r.)	n.r.	CG: p=0.624 [n]<br>IG: p<0.0001 [n]	n.r.	N/A
		balance score	6 (n.r.)	7 (n.r.)	n.r.	7 (n.r.)	11 (n.r.)	n.r.	CG: p=0.640 [n]<br>IG: p<0.0001 [n]	n.r.	N/A
		gait score	4 (n.r.)	4 (n.r.)	n.r.	5 (n.r.)	7 (n.r.)	n.r.	CG: p=0.530 [n]<br>IG: p<0.0001 [n]	n.r.	N/A
	TUG [s]		32.1 (n.r.)	33.3 (n.r.)	n.r.	32.6 (n.r.)	31.1	n.r.	CG: p=0.171 [n]<br>IG: p<0.0001 [n]	n.r.	N/A

Table S3. Data extraction first search *(Continued)*

Reference	Motor assessments		CG			IG			Statistics / p-value	Effect size	
			Baseline Mean (SD)	Post Mean (SD)	Difference Mean (SD)	Baseline Mean (SD)	Post Mean (SD)	Difference Mean (SD)		reported	calculated [cc]
Lam et al., 2018 [68]	BBS		42.5 (10.7)	45.5 (10.6)	3.1 (n.r.)	43.6 (9.7)	45.2 (8.9)	1.6 (n.r.)	p=0.571 [o]	0.011 [z]	N/A
	POMA	total score	24.3 (5.8)	25.4 (5.5)	1.2 (n.r.)	25.4 (3.8)	26.0 (4.0)	0.6 (n.r.)	p=0.382 [o]	0.017 [z]	N/A
		balance score	14.1 (2.3)	14.0 (3.4)	0.7 (n.r.)	14.1 (2.3)	14.7 (2.4)	0.7 (n.r.)	p=0.705 [o]	0.006 [z]	N/A
		gait score	11.3 (1.6)	11.4 (2.3)	0.5 (n.r.)	11.3 (1.6)	11.3 (1.8)	-0.1 (n.r.)	p=0.178 [o]	0.034 [z]	N/A
	TUG [s]		23.0 (15.7)	21.3 (15.9)	-1.8 (n.r.)	20.3 (10.5)	19.8 (12.6)	-0.5 (n.r.)	p=0.707 [o]	0.006 [z]	N/A
	5x STS [s]		25.7 (16.6)	22.9 (11.2)	-2.8 (n.r.)	21.6 (8.2)	21.0 (8.4)	-0.6 (n.r.)	p=0.720 [o]	0.006 [z]	N/A
Miu et al., 2008 [57]	FR [cm]		19.9 (8.3)	n.r.	n.r.	19.7 (7)	n.r.	n.r.	IG: p=0.007 [m]	n.r.	N/A
	BBS		n.r.	n.r.	n.r.	n.r.	n.r.	n.r.	IG: p<0.001 [m]	n.r.	N/A
	6min WT [m]		264 (100)	n.r.	n.r.	245 (90)	n.r.	38 (42)	IG: p<0.001 [m]	n.r.	N/A
Netz et al., 2007 [58]	FR [cm]		19.3 (10.3)	22.9 (7.0)	3.6 (SE: 3.7)	20.1 (7.9)	21.7 (6.9)	1.6 (SE: 1.3)	n.s. [i]	n.r.	0.27
	TUG [s]		16.5 (9.3)	14.7 (7.3)	-1.8 (SE: 0.8)	17.8 (8.4)	18.1 (8.8)	0.3 (SE:0.7)	n.s. [i]	n.r.	0.85
	5x STS [s]		14.0 (4.1)	14.6 (4.1)	0.6 (SE: 1.0)	15.4 (4.2)	16.1 (5.8)	0.7 (SE: 1.0)	n.s. [i]	n.r.	0.03
Padala et al., 2017 [69]	BBS		45.8 (2.5)	n.r.	n.r.	46.5 (2.4)	n.r.	n.r.	p=0.048 [i]	n.r.	N/A
Padala et al., 2012 [70]	BBS		41.3 (7.6)	46.6 (8.7)	n.r.	43.4 (8.9)	49.6 (5.7)	n.r.	p=0.56 [i]	n.r.	N/A
	POMA		22.9 (2.6)	24.9 (3.4)	n.r.	23.5 (3.7)	25.3 (2.8)	n.r.	p=0.97 [i]	n.r.	N/A
	TUG [s]		14.9 (4.7)	12.8 (3.2)	n.r.	14.7 (7.2)	13.9 (7.9)	n.r.	p=0.52 [i]	n.r.	N/A
Pedrinolla et al., 2018 [93]	Gait analysis	speed [cm/s]	92.3 (5.7)	n.r.	n.r.	92.5 (10.2)	n.r.	n.r.	z=-1.77, p=0.076 [p]	n.r.	N/A

Table S3. Data extraction first search *(Continued)*

Reference	Motor assessments		CG Baseline Mean (SD)	CG Post Mean (SD)	CG Difference Mean (SD)	IG Baseline Mean (SD)	IG Post Mean (SD)	IG Difference Mean (SD)	Statistics / p-value	Effect size reported	Effect size calculated [cc]
Pedrinolla et al., 2018 [93]	Gait analysis	stride [cm]	104.3 (4.6)	n.r.	n.r.	111.3 (7.2)	n.r.	n.r.	z=-0.86, p=0.391 [p]	n.r.	N/A
		step [cm]	52.0 (2.3)	n.r.	n.r.	55.7 (3.6)	n.r.	n.r.	z=-0.72, p=0.471 [p]	n.r.	N/A
		single support [%]	36.1 (0.4)	n.r.	n.r.	37.2 (1.1)	n.r.	n.r.	z=0.00, p=1.000 [p]	n.r.	N/A
		double support [%]	27.5 (0.9)	n.r.	n.r.	26.2 (1.3)	n.r.	n.r.	z=0.85, p=0.394 [p]	n.r.	N/A
	3-speed walking test, VO_2 [ml/min*kg^{-1}]	speed 1	11.0 (0.9)	n.r.	n.r.	13.3 (0.6)	n.r.	n.r.	t=2.28, p=0.030 [a]	n.r.	0.81
		speed 2	12.0 (0.8)	n.r.	n.r.	12.0 (0.6)	n.r.	n.r.	t=2.94, p=0.006 [a]	n.r.	1.04
		speed 3	17.1 (1.0)	n.r.	n.r.	14.8 (0.7)	n.r.	n.r.	t=2.09, p=0.054 [a]	n.r.	0.74
	3-speed walking test, heart rate [bpm]	speed 1	93.0 (2.8)	n.r.	n.r.	98.3 (2.3)	n.r.	n.r.	t=1.58, p=0.126 [a]	n.r.	0.56
		speed 2	99.2 (2.8)	n.r.	n.r.	103.9 (3.0)	n.r.	n.r.	t=2.72, p=0.011 [a]	n.r.	0.96
		speed 3	106.8 (3.2)	n.r.	n.r.	108.2 (4.6)	n.r.	n.r.	t=1.75, p=0.107 [a]	n.r.	0.62
	3-speed walking test, energy cost of walking [J/kg*m^{-1}]	speed 1	6.5 (2.6)	n.r.	n.r.	6.2 (1.1)	n.r.	n.r.	z=2.04, p=0.041 [p]	n.r.	N/A
		speed 2	4.6 (1.0)	n.r.	n.r.	5.1 (0.5)	n.r.	n.r.	z=2.96, p=0.003 [p]	n.r.	N/A
		speed 3	5.9 (0.9)	n.r.	n.r.	6.2 (1.6)	n.r.	n.r.	z=1.47, p=0.142 [p]	n.r.	N/A
Pitkälä et al., 2013 [109]	SPPB		9.7 (2.1)	n.r.	n.r.	IG1: 9.3 (2.4) IG2: 9.8 (2.2)	n.r.	n.r.	p=0.90 [q]	n.r.	N/A

Table S3. Data extraction first search *(Continued)*

Reference	Motor assessments		CG Baseline Mean (SD)	CG Post Mean (SD)	CG Difference Mean (SD)	IG Baseline Mean (SD)	IG Post Mean (SD)	IG Difference Mean (SD)	Statistics / p-value	Effect size reported	Effect size calculated [cc]
Pomeroy et al., 1999 [125]	Southampton Assessment of Mobility		13.3 (6.0)	12.2 (6.4)	-1.1 (4.1)	15.4 (4.1)	15.1 (4.2)	-0.4 (2.2)	p=0.614 [b]	n.r.	0.22
	2-min walk test [m]		23.8 (22.1)	24.4 (20.4)	n.r.	32.2 (15.7)	35.3 (18.6)	3.1 (9.3)	p=0.325 [a]	n.r.	N/A
Roach et al., 2011 [105]	Acute Care Index of Function	transfer score	0.8 (0.2)	0.8 (0.3)	n.r.	IG1: 0.8 (0.2)<br>IG2: 0.9 (0.2)	IG1: 0.9 (0.2)<br>IG2: 0.8 (0.2)	n.r.	p=0.04 [i]	n.r.	N/A
		bed mobility score	0.9 (0.3)	0.8 (0.3)	n.r.	IG1: 0.9 (0.2)<br>IG2: 0.8 (0.3)	IG1: 0.9 (0.2)<br>IG2: 0.8 (0.3)	n.r.	p=0.77 [i]	n.r.	N/A
	6min WT [ft]		296.6 (229.4)	324.8 (274.4)	n.r.	IG1: 387.1 (214.8)<br>IG2: 329.9 (247.4)	IG1: 384.9 (217.6)<br>IG2: 367.5 (300.2)	n.r.	p=0.61 [i]	n.r.	N/A
Rolland et al., 2007 [89]	One-leg balance test [% abnormal score]		(92.5%)	(98.1%)	n.r.	(91.0%)	(94.6%)	n.r.	p=0.34 [r]	n.r.	N/A
	Get-Up and Go Test		2.7 (0.8)	3.2 (1.2)	n.r.	2.7 (0.8)	3.1 (1.1)	n.r.	p=0.31 [r]	n.r.	N/A
	6m WT [m/s]		0.3 (0.1)	0.4 (0.2)	n.r.	0.3 (0.1)	0.4 (0.2)	n.r.	p=0.002 [r]	n.r.	N/A
Santana-Sosa et al., 2008 [80]	POMA		n.r.	n.r.	n.r.	n.r.	n.r.	n.r.	F(1,14)=45.13, p<0.001 [i]	0.887 [z]	3.59
	Senior Fitness test	30s CST	n.r.	n.r.	n.r.	n.r.	n.r.	n.r.	F(1,14)=48.74, p<0.001 [i]	0.777 [z]	3.73
		Arm curl test	n.r.	n.r.	n.r.	n.r.	n.r.	n.r.	F(1,14)=73.15, p<0.001 [i]	0.839 [z]	N/A
		Chair sit-and-reach test [cm]	n.r.	n.r.	n.r.	n.r.	n.r.	n.r.	F(1,14)=40.18, p<0.001 [i]	0.742 [z]	N/A
		Back scratch test [cm]	n.r.	n.r.	n.r.	n.r.	n.r.	n.r.	F(1,14)=36.04, p<0.001 [i]	0.720 [z]	N/A

Table S3. Data extraction first search *(Continued)*

Reference	Motor assessments		CG Baseline Mean (SD)	CG Post Mean (SD)	CG Difference Mean (SD)	IG Baseline Mean (SD)	IG Post Mean (SD)	IG Difference Mean (SD)	Statistics / p-value	Effect size reported	Effect size calculated [cc]
Santana-Sosa et al., 2008 [80]	Senior Fitness test	8-foot up-and-go test [s]	n.r.	n.r.	n.r.	n.r.	n.r.	n.r.	F(1,14)=36.78, p<0.001 [i]	0.724 [z]	N/A
		2-min step test	n.r.	n.r.	n.r.	n.r.	n.r.	n.r.	F(1,14)=8.96, p=0.010 [i]	0.390 [z]	N/A
Schwenk et al., 2014 [94]	Postural sway (inertial sensors) [sq cm]		6.6 (0.8)	6.5 (0.8)	n.r.	6.5 (0.6)	6.1 (0.6)	n.r.	p=0.023 [i]	0.06 [z]	N/A
	Gait analysis	speed [cm/sec]	73.3 (37.1)	89.4 (36.8)	n.r.	72.7 (38.6)	92.8 (37.6)	n.r.	p=0.354 [i]	0.01 [z]	N/A
		stride length [cm]	80.9 (28.9)	91.2 (27.8)	n.r.	83.2 (31.0)	96.4 (29.9)	n.r.	p=0.354 [i]	0.01 [z]	N/A
		cadence [steps/min]	105.8 (22.0)	115.7 (19.8)	n.r.	100.6 (25.7)	113.5 (24.6)	n.r.	p=0.343 [i]	0.01 [z]	N/A
	Hierarchical Assessment of Balance and Mobility		40.9 (13.6)	46.4 (13.0)	n.r.	38.5 (15.1)	46.6 (10.2)	n.r.	p=0.162 [i]	0.02 [z]	N/A
	5x STS [s]		16.4 (6.8)	15.8 (10.9)	n.r.	17.0 (6.4)	13.1 (4.6)	n.r.	p=0.037 [i]	0.06 [z]	N/A
	Handgrip dynamometer [kg]		14.6 (6.2)	15.1 (6.6)	n.r.	14.4 (6.2)	14.8 (6.7)	n.r.	p=0.834 [i]	0.00 [z]	N/A
	One-repetition maximum in leg press [kg]		97.0 (51.0)	102.2 (54.4)	n.r.	99.7 (59.4)	140.0 (70.2)	n.r.	p<0.001 [i]	0.36 [z]	N/A
	One-repetition maximum of abductor [kg]		66.9 (28.8)	69.7 (29.0)	n.r.	70.8 (34.9)	88.2 (36.8)	n.r.	p<0.001 [i]	0.11 [z]	N/A
Schwenk et al., 2014 [95]	Gait analysis	speed [cm/sec]	128.7 (38.2)	127.6 (35.7)	n.r.	132.7 (55.7)	149.3 (48.2)	n.r.	p<0.001 [c]	1.27 [y]	N/A
		cadence [steps/min]	134.5 (17.9)	132.0 (19.2)	n.r.	137.1 (21.1)	145.4 (20.8)	n.r.	p=0.002 [c]	0.96 [y]	N/A
		stride length [cm]	115.3 (29.5)	115.9 (25.7)	n.r.	116.6 (42.6)	124.8 (37.4)	n.r.	p=0.008 [c]	0.80 [y]	N/A

Table S3. Data extraction first search *(Continued)*

Refer-ence	Motor assessments		CG			IG			Statistics / p-value	Effect size	
			Baseline Mean (SD)	Post Mean (SD)	Difference Mean (SD)	Baseline Mean (SD)	Post Mean (SD)	Difference Mean (SD)		reported	calcu-lated [cc]
Schwenk et al., 2014 [95]	Gait analysis	stride time [sec]	0.9 (0.1)	0.9 (0.1)	n.r.	0.9 (0.2)	0.8 (0.1)	n.r.	p=0.001 [c]	0.99 [y]	N/A
		double support [%]	25.9 (6.1)	25.4 (6.0)	n.r.	26.9 (8.9)	23.0 (7.8)	n.r.	p=0.001 [c]	1.03 [y]	N/A
		step width [cm]	10.2 (4.2)	9.9 (4.4)	n.r.	11.3 (4.2)	11.1 (5.0)	n.r.	p=0.999 [c]	0.00 [y]	N/A
		step time variability [CV]	5.0 (2.5)	5.4 (2.6)	n.r.	5.2 (3.4)	5.1 (2.1)	n.r.	p=0.425 [c]	0.22 [y]	N/A
		Walk-Ratio	0.4 (0.1)	0.4 (0.1)	n.r.	0.4 (0.2)	0.4 (0.2)	n.r.	p=0.554 [c]	0.18 [y]	N/A
Schwenk et al., 2010 [126]	Gait analysis, dual task cost, serial 2 condition [%]	gait speed	-22.6 (18.4)	-20.8 (15.8)	n.r.	-21.9 (11.9)	-13.5 (9.4)	n.r.	p=0.086 [i]	n.r.	N/A
		cadence	-18.8 (15.4)	-14.9 (12.5)	n.r.	-17.5 (10.4)	-12.8 (10.1)	n.r.	p=0.846 [i]	n.r.	N/A
		stride length	-5.7 (12.3)	-6.8 (13.6)	n.r.	-5.3 (9.2)	-0.1 (8.2)	n.r.	p=0.074 [i]	n.r.	N/A
		stride time	30.4 (45.8)	21.0 (23.0)	n.r.	24.1 (20.0)	16.8 (17.7)	n.r.	p=0.750 [i]	n.r.	N/A
		single support	-6.0 (10.7)	-6.2 (7.0)	n.r.	-4.7 (6.4)	-3.2 (4.2)	n.r.	p=0.459 [i]	n.r.	N/A
		motor + cognitive perfor-mance	-14.7 (21.3)	-13.6 (17.1)	n.r.	-18.1 (15.2)	-12.3 (7.8)	n.r.	p=0.378 [i]	n.r.	N/A
	Gait analysis, dual task cost, serial 3 condition [%]	gait speed	-39.8 (18.9)	-37.2 (16.7)	n.r.	-41.6 (18.4)	-20.0 (12.7)	n.r.	p<0.001 [i]	n.r.	N/A
		cadence	-26.8 (15.8)	-23.6 (14.1)	n.r.	-27.9 (18.5)	-15.3 (11.0)	n.r.	p=0.007 [i]	n.r.	N/A
		stride length	-18.8 (14.0)	-18.0 (15.6)	n.r.	-20.7 (12.2)	-5.6 (11.7)	n.r.	p=0.001 [i]	n.r.	N/A
		stride time	44.0 (35.8)	35.8 (27.8)	n.r.	62.0 (102.0)	20.9 (20.7)	n.r.	p=0.056 [i]	n.r.	N/A
		single support	-9.7 (11.3)	-10.6 (8.7)	n.r.	-13.8 (13.4)	-5.1 (5.0)	n.r.	p=0.003 [i]	n.r.	N/A

Table S3. Data extraction first search *(Continued)*

Reference	Motor assessments		CG Baseline Mean (SD)	CG Post Mean (SD)	CG Difference Mean (SD)	IG Baseline Mean (SD)	IG Post Mean (SD)	IG Difference Mean (SD)	Statistics / p-value	Effect size reported	Effect size calculated [cc]
Schwenk et al., 2010 [126]	Gait analysis, dual task cost, serial 3 condition [%]	motor + cognitive performance	-31.9 (20.3)	-29.7 (17.5)	n.r.	-32.7 (24.8)	-12.1 (18.4)	n.r.	p=0.026 [i]	n.r.	N/A
Sobol et al., 2016 [84]	TUG [s]		6.6 (1.62)	6.6 (1.9)	n.r.	6.7 (1.8)	6.5 (1.7)	n.r.	p=0.151 [s]	n.r.	N/A
	10-metre walk test [m/s]	single task	1.4 (0.3)	1.4 (0.2)	n.r.	1.3 (0.2)	1.3 (0.2)	n.r.	p=0.108 [s]	n.r.	N/A
		dual task months	1.0 (0.3)	1.1 (0.3)	n.r.	0.9 (0.3)	1.0 (0.4)	n.r.	p=0.051 [s]	n.r.	N/A
	10-metre walk test [m/s]	dual task numbers	1.1 (0.3)	1.1 (0.3)	n.r.	0.9 (0.3)	1.0 (0.4)	n.r.	p=0.155 [s]	n.r.	N/A
	30s CST		14.9 (4.2)	15.5 (4.3)	n.r.	13.9 (3.6)	14.3 (3.5)	n.r.	p=0.408 [s]	n.r.	N/A
	400-m walk test [s]		305 (71.5)	303 (70.5)	n.r.	306 (92.3)	296 (79.1)	n.r.	p=0.118 [s]	n.r.	N/A
	6-min Astrand Cycle Ergometer test, VO_{2max} [ml/kg/min]		26.2 (9.0)	27.2 (8.7)	n.r.	25.3 (7.5)	30.1 (7.5)	n.r.	p<0.0001 [s]	n.r.	N/A
Souto Barreto et al., 2017 [90]	4m WT [m/s]		0.5 (0.2)	n.r.	0.03 (SE: 0.03)	0.5 (0.2)	n.r.	0.07 (SE: 0.03)	β=0.01, p=0.30 [t]	n.r.	0.20
	SPPB		4.5 (2.3)	n.r.	-0.8 (SE: 0.34)	4.4 (2.4)	n.r.	-0.2 (SE: 0.36)	β=0.10, p=0.22 [t]	n.r.	0.26
Steinberg et al., 2009 [98]	8-foot walk test [s]		3.7 (1.6)	n.r.	n.r.	3.6 (1.8)	n.r.	n.r.	β=-0.08 (0.27), p=0.77 [u]	n.r.	N/A
	5x STS [s]		16.1 (6.5)	n.r.	n.r.	16.8 (7.4)	n.r.	n.r.	β=-4.4 (3.6), p=0.22 [u]	n.r.	N/A
	Jebsen Total Time [s]		107.3 (49.9)	n.r.	n.r.	83.5 (41.9)	n.r.	n.r.	β=-23.39 (11.6), p=0.04 [u]	n.r.	N/A

Table S3. Data extraction first search *(Continued)*

Refer-ence	Motor assessments		CG			IG			Statistics / p-value	Effect size	
			Baseline Mean (SD)	Post Mean (SD)	Difference Mean (SD)	Baseline Mean (SD)	Post Mean (SD)	Difference Mean (SD)		reported	calcu-lated [cc]
Suttanon et al., 2013 [52]	Modified Clinical Test of Sensory Interaction of Balance [deg/s]		1.5 (0.7)	1.7 (0.8)	n.r.	1.9 (0.7)	1.8 (0.7)	n.r.	p=0.086 [s]	n.r.	N/A
	Limits of stability	reaction time [ms]	1.1 (0.2)	1.2 (0.3)	n.r.	1.2 (0.3)	1.1 (0.2)	n.r.	p=0.365 [s]	n.r.	N/A
		Move-ment velocity [degrees/s]	3.1 (1.2)	3.4 (1.0)	n.r.	3.0 (1.3)	3.0 (1.1)	n.r.	p=0.016 [s]	n.r.	N/A
		maximum excursion [%]	72.4 (12.0)	72.7 (12.1)	n.r.	66.3 (14.4)	68.3 (15.5)	n.r.	p=0.817 [s]	n.r.	N/A
	Limits of stability	directional control [%]	64.4 (10.0)	61.3 (11.0)	n.r.	60.3 (12.3)	60.7 (11.3)	n.r.	p=0.446 [s]	n.r.	N/A
	FR [cm]		28.5 (4.7)	25.5 (5.3)	n.r.	23.5 (5.7)	25.8 (5.6)	n.r.	p=0.002 [s]	n.r.	N/A
	Hill Step Test		13.0 (3.2)	11.8 (3.5)	n.r.	12.3 (2.4)	12.3 (3.0)	n.r.	p=.082 [s]	n.r.	N/A
	Step quick turn, worse side	time [s]	3.3 (1.0)	3.1 (1.1)	n.r.	3.8 (1.7)	3.7 (2.0)	n.r.	p=0.283 [s]	n.r.	N/A
		sway [deg]	48.9 (8.2)	47.3 (6.7)	n.r.	49.0 (11.1)	48.5 (13.0)	n.r.	p=0.452 [s]	n.r.	N/A
	TUG [s]		16.4 (6.6)	16.6 (6.2)	n.r.	16.2 (5.0)	16.2 (5.6)	n.r.	p=0.571 [s]	n.r.	N/A
	Cognitive TUG [s]		18.1 (3.4)	19.2 (6.0)	n.r.	25.4 (8.0)	23.2 (7.7)	n.r.	p=0.994 [s]	n.r.	N/A
	Manual TUG [s]		18.0 (6.8)	19.0 (7.3)	n.r.	18.4 (5.8)	18.2 (6.6)	n.r.	p=0.088 [s]	n.r.	N/A
	Gait analysis	step width [cm]	15.6 (4.5)	16.2 (4.0)	n.r.	16.2 (2.3)	15.6 (2.5)	n.r.	p=0.125 [s]	n.r.	N/A
		step length [cm]	36.8 (13.2)	36.0 (9.5)	n.r.	32.5 (8.3)	31.8 (10.7)	n.r.	p=0.907 [s]	n.r.	N/A
		speed [cm/s]	40.4 (13.5)	41.7 (14.3)	n.r.	39.4 (11.6)	38.9 (13.6)	n.r.	p=0.244 [s]	n.r.	N/A
	5x STS [s]		13.3 (5.0)	13.3 (3.7)	n.r.	13.2 (4.2)	14.6 (5.1)	n.r.	p=0.945 [s]	n.r.	N/A

Table S3. Data extraction first search *(Continued)*

Reference	Motor assessments		CG Baseline Mean (SD)	CG Post Mean (SD)	CG Difference Mean (SD)	IG Baseline Mean (SD)	IG Post Mean (SD)	IG Difference Mean (SD)	Statistics / p-value	Effect size reported	Effect size calculated [cc]
Suttanon et al., 2013 [52]	STS on NeuroCom Balance Master	raising index [% body weight]	16.3 (4.8)	17.0 (6.9)	n.r.	13.5 (4.7)	14.5 (6.1)	n.r.	p=0.725 [s]	n.r.	N/A
		sway [deg/s]	4.2 (1.3)	4.7 (1.5)	n.r.	4.0 (1.1)	4.3 (1.1)	n.r.	p=0.290 [s]	n.r.	N/A
	Physiological Profile Assessment [score]		1.4 (1.2)	1.8 (1.2)	n.r.	1.8 (1.2)	1.9 (0.3)	n.r.	p=0.314 [s]	n.r.	N/A
Tappen et al., 2000 [106]	6min WT [ft]		261.1 (175.0)	212.1 (168.8)	n.r.	IG1: 330.2 (250.0)<br>IG2: 391.7 (233.3)	IG1: 321.9 (223.2)<br>IG2: 310.6 (219.3)	n.r.	p<0.05 [i]<br>CG: p=0.0874 [e]<br>IG1: n.s. [e]<br>IG2: p=0.0119 [e]<br>F=5.59, p<0.01 [v]	n.r.	N/A
Telenius et al., 2015 [71]	BBS [score]		35.4 (13.7)	36.6 (14.4)	n.r.	34.3 (14.5)	37.2 (14.0)	n.r.	p=0.02 [a]	0.4 [y]	N/A
	6m WT [m/s]		0.5 (0.2)	0.5 (0.3)	n.r.	0.5 (0.2)	0.5 (0.2)	n.r.	p=0.86 [a]	0.0 [y]	N/A
	30s CST		6.2 (2.9)	6.6 (3.7)	n.r.	6.0 (3.1)	7 (3.3)	n.r.	p=0.11 [a]	0.2 [y]	N/A
Toots et al., 2017 [91]	4m WT [m/s]	walking aid	0.5 (0.2)	n.r.	-0.02 (SE: 0.02)	0.5 (0.2)	n.r.	-0.02 (SE: 0.02)	p=0.777 [w]	-0.05 [bb]	0.05
		no walking aid	0.5 (0.2)	n.r.	-0.02 (SE: 0.02)	0.4 (0.2)	n.r.	0.01 (SE: 0.02)	p=0.242 [w]	0.20 [bb]	0.20
Toots et al., 2016 [72]	BBS		29.3 (14.7)	n.r.	-1.8 (SE: 0.9)	28.6 (14.3)	n.r.	2.4 (SE: 0.9)	p<0.001 [w]	0.52 [bb]	0.53
Toulotte et al., 2003 [85]	Postural sway (posturography platform) [mm²]		292.3 (94.5)	n.r.	n.r.	398.7 (229.6)	n.r.	n.r.	p<0.01 [i]	n.r.	N/A
	TUG [s]		39.4 (17.7)	n.r.	n.r.	67.6 (38.9)	n.r.	n.r.	p<0.01 [i]	n.r.	N/A
	10-metre walk test [s]		63.4 (51.1)	n.r.	n.r.	60.6 (49.9)	n.r.	n.r.	p<0.05 [i]	n.r.	N/A

Table S3. Data extraction first search *(Continued)*

Reference	Motor assessments		CG Baseline Mean (SD)	CG Post Mean (SD)	CG Difference Mean (SD)	IG Baseline Mean (SD)	IG Post Mean (SD)	IG Difference Mean (SD)	Statistics / p-value	Effect size reported	Effect size calculated [cc]
Toulotte et al., 2003 [85]	Chair sit-and-reach test [cm]		10.3 (8.0)	n.r.	n.r.	11.4 (7.1)	n.r.	n.r.	p<0.05 [i]	n.r.	N/A
Venturelli et al., 2011 [107]	6min WT [m]		238 (47)	168 (34)	n.r.	245 (31)	294 (49)	n.r.	p<0.001 [i]	n.r.	N/A
Vreugdenhil et al., 2012 [59]	FR [cm]		24.0 (6.4)	22.1 (7.9)	-1.9 (SE: 1.3)	27.6 (7.4)	30.6 (7.0)	2.3 (SE: 1.1)	p=0.032 [s]	n.r.	0.80
	TUG [s]		11.1 (3.3)	12.8 (4.1)	2.0 (SE: 0.7)	9.7 (3.7)	9.1 (3.8)	-0.9 (SE: 0.5)	p=0.004 [s]	n.r.	1.09
	10-s chair-stand test		8.5 (2.9)	7.2 (3.2)	-1.0 (SE: 0.4)	9.2 (2.5)	10.8 (2.0)	1.7 (SE: 0.4)	p<0.001 [s]	n.r.	1.55
Werner et al., 2017 [100]	Body-fixed-sensor-based STS analysis (DynaPort)	trunk flexion, range [°]	33.1 (9.9)	34.9 (10.5)	0.9 (9.0)	33.6 (7.5)	40.2 (12.8)	8.3 (13.4)	p=0.006 [i]	0.099 [z]	0.66
		trunk flexion, duration [s]	1.1 (0.5)	1.1 (0.5)	-0.1 (0.6)	1.2 (0.9)	1.8 (1.1)	0.8 (1.2)	p<0.001 [i]	0.188 [z]	0.96
		maximum trunk flexion, angular velocity [°/s]	73.9 (26.6)	81.4 (26.8)	5.9 (22.0)	79.0 (27.4)	64.8 (27.1)	-12.3 (26.4)	p=0.002 [i]	0.127 [z]	0.76
		STS movement duration [s]	2.1 (0.9)	2.0 (0.7)	-0.1 (0.9)	2.1 (1.1)	2.8 (1.4)	0.9 (1.5)	p<0.001 [i]	0.158 [z]	0.87
	ACSID	recall and initiation score	1.8 (1.0)	1.7 (.9)	-0.1 (0.9)	1.8 (1.0)	3.3 (1.3)	1.7 (1.7)	p<0.001 [i]	0.319 [z]	1.35

Table S3. Data extraction first search *(Continued)*

Reference	Motor assessments		CG			IG			Statistics / p-value	Effect size	
			Baseline Mean (SD)	Post Mean (SD)	Difference Mean (SD)	Baseline Mean (SD)	Post Mean (SD)	Difference Mean (SD)		reported	calculated [cc]
Werner et al., 2017 [100]	ACSID	effective performance score	1.9 (.9)	1.8 (.8)	-0.1 (0.9)	2.1 (.9)	3.1 (1.0)	1.0 (0.9)	p<0.001 [i]	0.261 [z]	1.24
		total score	3.7 (1.4)	3.5 (1.5)	-0.1 (1.4)	3.9 (1.4)	6.4 (2.1)	2.7 (2.2)	p<0.001 [i]	0.372 [z]	1.54
Wesson et al., 2013 [61]	Near-tandem test [n.r.]		5.7 (3.0)	6.3 (3.7)	n.r.	5.2 (3.6)	5.4 (3.7)	n.r.	p=0.32 [b]	n.r.	N/A
	Hill Step Test		14.5 (5.0)	14.2 (7.7)	n.r.	19.2 (6.5)	15.0 (5.1)	n.r.	p=0.1 [b]	n.r.	N/A
	Physiological Profile Assessment		1.7 (1.7)	2.7 (1.8)	n.r.	0.8 (1.2)	1.4 (1.6)	n.r.	p=0.82 [b]	n.r.	N/A
Wiloth et al., 2018 [54]	Physiomat-Follow-The-Ball Task, trained	duration [s]	28.9 (15.7)	23.4 (5.5)	n.r.	30.9 (17.5)	19.3 (4.6)	n.r.	p<0.001 [c]	0.253 [aa]	N/A
		accuracy [digits/ms]	4164.3 (3922.4)	3776.3 (1286.9)	n.r.	4450.4 (2859.8)	3169.7 (557.2)	n.r.	p<0.001 [c]	0.144 [aa]	N/A
	Physiomat-Trail-Making Task, Level 1 trained	duration [s]	11.5 (4.7)	9.7 (3.1)	n.r.	16.7 (20.3)	7.2 (1.9)	n.r.	p<0.001 [c]	0.260 [aa]	N/A
		accuracy [digits/ms]	2108.1 (911.9)	2124.9 (773.9)	n.r.	2849.1 (4199.2)	1782.0 (339.7)	n.r.	p=0.007 [c]	0.092 [aa]	N/A
	Physiomat-Trail-Making Task, Level 2 trained	duration [s]	19.9 (11.5)	16.7 (4.9)	n.r.	21.8 (9.7)	14.3 (5.6)	n.r.	p<0.001 [c]	0.311 [aa]	N/A
		accuracy [digits/ms]	3005.7 (1066.2)	3187.0 (940.1)	n.r.	3390.1 (1800.1)	2923.1 (803.0)	n.r.	p=0.003 [c]	0.127 [aa]	N/A
	Physiomat-Trail-Making Task, Level 3 trained	duration [s]	25.9 (10.4)	22.7 (5.3)	n.r.	28.6 (11.8)	20.0 (7.4)	n.r.	p<0.001 [c]	0.293 [aa]	N/A
		accuracy [digits/ms]	3742.8 (557.8)	3992.0 (945.8)	n.r.	4376.7 (1528.5)	3806.1 (1246.3)	n.r.	p=0.047 [c]	0.065 [aa]	N/A
	Physiomat-Trail-Making Task, Level 4 trained	duration [s]	43.9 (9.0)	44.0 (15.5)	n.r.	51.3 (16.6)	34.9 (7.9)	n.r.	p<0.001 [c]	0.340 [aa]	N/A
		accuracy [digits/ms]	7724.4 (1676.4)	7880.0 (2238.8)	n.r.	8176.3 (2484.2)	6599.3 (1468.6)	n.r.	p<0.001 [c]	0.365 [aa]	N/A

Table S3. Data extraction first search *(Continued)*

Reference	Motor assessments		CG			IG			Statistics / p-value	Effect size	
			Baseline Mean (SD)	Post Mean (SD)	Difference Mean (SD)	Baseline Mean (SD)	Post Mean (SD)	Difference Mean (SD)		reported	calculated [cc]
Wiloth et al., 2018 [54]	Physiomat-Trail-Making Task, Level 5 trained	duration [s]	58.7 (17.6)	56.2 (16.7)	n.r.	56.3 (12.1)	48.7 (14.7)	n.r.	p<0.001 [c]	0.589 [aa]	N/A
		accuracy [digits/ms]	8467.8 (1646.8)	9360.2 (2855.1)	n.r.	8444.3 (2261.5)	8005.0 (1906.4)	n.r.	p=0.007 [c]	0.329 [aa]	N/A
	Physiomat-Trail-Making Task score, trained		3.2 (1.5)	3.6 (1.4)	n.r.	3.4 (1.4)	4.7 (.9)	n.r.	p<0.001 [c]	0.211 [aa]	N/A
	Physiomat-Trail-Making Task, Level 1 untrained	duration [s]	11.2 (5.4)	9.7 (3.1)	n.r.	15.6 (15.6)	7.5 (2.1)	n.r.	p<0.001 [c]	0.219 [aa]	N/A
		accuracy [digits/ms]	2043.6 (1246.8)	1959.8 (543.6)	n.r.	2523.1 (3040.6)	1735.4 (317.1)	n.r.	p=0.017 [c]	0.073 [aa]	N/A
	Physiomat-Trail-Making Task, Level 2 untrained	duration [s]	17.9 (8.4)	14.7 (3.7)	n.r.	18.0 (7.4)	13.9 (9.9)	n.r.	p<0.001 [c]	0.236 [aa]	N/A
		accuracy [digits/ms]	2770.9 (1598.6)	2661.6 (785.8)	n.r.	2703.4 (1586.1)	2683.1 (1746.5)	n.r.	p=0.121 [c]	0.037 [aa]	N/A
	Physiomat-Trail-Making Task, Level 3 untrained	duration [s]	29.6 (15.9)	25.4 (7.0)	n.r.	32.9 (16.5)	23.1 (10.9)	n.r.	p<0.001 [c]	0.204 [aa]	N/A
		accuracy [digits/ms]	4467.0 (1093.5)	4816.0 (1671.0)	n.r.	5316.9 (2637.1)	4539.6 (2924.3)	n.r.	p=0.008 [c]	0.122 [aa]	N/A
	Physiomat-Trail-Making Task, Level 4 untrained	duration [s]	41.4 (9.5)	38.7 (12.2)	n.r.	38.7 (11.8)	33.0 (7.7)	n.r.	p=0.005 [c]	0.280 [aa]	N/A
		accuracy [digits/ms]	8137.4 (2374.7)	7671.3 (3642.1)	n.r.	7285.2 (2346.8)	5857.7 (1296.5)	n.r.	p=0.009 [c]	0.244 [aa]	N/A
	Physiomat-Trail-Making Task, Level 5 untrained	duration [s]	55.5 (11.8)	54.5 (15.1)	n.r.	55.7 (16.5)	48.5 (13.7)	n.r.	p=0.003 [c]	0.384 [aa]	N/A
		accuracy [digits/ms]	8598.2 (1539.8)	9027.3 (2444.4)	n.r.	9227.8 (4138.9)	8278.5 (2000.1)	n.r.	p=0.001 [c]	0.459 [aa]	N/A
	Physiomat-Trail-Making Task score, untrained		3.0 (1.5)	3.5 (1.4)	n.r.	3.1 (1.4)	4.5 (1.0)	n.r.	p<0.001 [c]	0.184 [aa]	N/A

Table S3. Data extraction first search *(Continued)*

Reference	Motor assessments		CG			IG			Statistics / p-value	Effect size	
			Baseline Mean (SD)	Post Mean (SD)	Difference Mean (SD)	Baseline Mean (SD)	Post Mean (SD)	Difference Mean (SD)		reported	calculated [cc]
Yoon et al, 2013 [73]	Postural sway (Wii Balance Board), eyes closed	wide base, COP velocity [cm/s]	3.5 (1.7)	3.2 (1.3)	0.8 (1.8)	4.8 (1.4)	3.6 (1.2)	1.2 (1.0)	$p<0.05$ [b] IG: $p<0.05$ [x]	n.r.	0.30
		narrow base, COP path length [cm/s]	104.7 (46.6)	95.4 (37.5)	9.3 (14.1)	146.4 (64.5)	93.9 (38.9)	52.5 (59.7)	$p<0.05$ [b] IG: $p<0.05$ [x]	n.r.	1.00
	BBS		34.9 (4.6)	35.1 (4.4)	-0.2 (2.4)	35.3 (1.8)	38.0 (2.0)	-2.7 (0.9)	$p<0.05$ [b] CG / IG: $p<0.05$ [x]	n.r.	1.52
	TUG [s]		28.8 (5.7)	n.r.	n.r.	27.7 (6.1)	n.r.	n.r.	n.r.	n.r.	N/A

4m WT: 4-metre walk test, 5x STS: Five Times Sit-to-Stand Test, 6m WT: 6-metre walk test, 6min WT: 6-minute walk test, 30s CST: 30-second chair stand test, ACSID: Assessment of Compensatory Sit-to-Stand Maneuvers in People With Dementia, BBS: Berg Balance Scale, bpm: beats per minute, CG: control group, COP: centre of pressure, E-ADL Test: Erlangen Test of Activities of Daily Living, FICSIT-4: Frailty and Injuries: Cooperative Studies of Intervention Techniques - subtest 4, FR: Functional Reach Test, GMWT: Groningen Meander Walking Test, IG: intervention group, N/A: not applicable, n.s.: not significant, POMA: Performance Oriented Mobility Assessment, SD: standard deviation, SE: standard error, SPPB: Short Physical Performance Battery, STS: Sit-to-Stand, TUG: Timed Up & Go Test, VO_2(max): (maximum) oxygen uptake

[a] independent t-test, between-group baseline-post difference, [b] Mann-Whitney test, between-group baseline-post difference, [c] Analysis of covariance with baseline scores as covariates, between-group post difference, [d] Kruskal-Wallis tests, between-group baseline-post difference, [e] dependent t-test, within-group baseline-post difference, [f] independent t-test, between-group post difference, [g] mixed model analysis of covariance with repeated measures, [h] general linear model analysis of covariance with repeated-measures, [i] two-way analysis of variance with repeated measures, group*time interaction, [j] one-way analysis of variance, between-group post difference, [k] linear regression analyses, between-group baseline-post difference, [l] Tukey test, within-group baseline-post difference, [m] one-way analysis of variance/analysis of variance with repeated measures, within-group baseline-post difference, [n] Friedman analysis of variance, within-group baseline-post difference, [o] mixed design multivariate analysis of variance, group*time interaction, [p] Wilcoxon's test, between-group baseline-post difference, [q] Analysis of covariance with age, sex, and use of mobility devices, between-group baseline-post difference, [r] ?, between-group baseline-post difference, [s] general linear model analysis, between-group baseline-post difference, [t] three level regression model, between-group baseline-post difference, [u] linear random effects models, time*group interaction, [v] Analysis of covariance with baseline scores and treatment fidelity as covariates, between-group post difference, [w] linear mixed effects models between-group baseline-post difference, [x] Wilcoxon's signed-rank test, within-group baseline-post difference, [y] standardized mean difference/Cohen's d, between-group, [z] partial eta squared, time*group interaction effect, [aa] partial eta squared, group effect, [bb] between-group difference/unadjusted pooled standard deviation of baseline-post difference, [cc] Cohen's d, time*group interaction

* Intention-to-treat analysis and complete-case analysis

References

50. Bossers WJR, van der Woude LHV, Boersma F, Hortobágyi T, Scherder EJA, van Heuvelen MJG. A 9-Week Aerobic and Strength Training Program Improves Cognitive and Motor Function in Patients with Dementia: A Randomized, Controlled Trial. Am J Geriatr Psychiatry. 2015;23:1106–16. doi:10.1016/j.jagp.2014.12.191.

52. Suttanon P, Hill KD, Said CM, Williams SB, Byrne KN, LoGiudice D, et al. Feasibility, safety and preliminary evidence of the effectiveness of a home-based exercise programme for older people with Alzheimer's disease: A pilot randomized controlled trial. Clin Rehabil. 2013;27:427–38. doi:10.1177/0269215512460877.

54. Wiloth S, Werner C, Lemke NC, Bauer J, Hauer K. Motor-cognitive effects of a computerized game-based training method in people with dementia: A randomized controlled trial. Aging Ment Health. 2018;22:1124–35. doi:10.1080/13607863.2017.1348472.

56. Arcoverde C, Deslandes A, Moraes H, Almeida C, Araujo NBd, Vasques PE, et al. Treadmill training as an augmentation treatment for Alzheimer's disease: A pilot randomized controlled study. Arq Neuropsiquiatr. 2014;72:190–6. doi:10.1590/0004-282X20130231.

57. Miu D, Szeto S, Mak Y. A randomized controlled trial on the effect of exercise on physical, cognitive, and affective function in dementia subjects. Asian J Gerontol Geriatr. 2008;3:8–16.

58. Netz Y, Axelrad S, Argov E. Group physical activity for demented older adults feasibility and effectiveness. Clin Rehabil. 2007;21:977–86. doi:10.1177/0269215507078318.

59. Vreugdenhil A, Cannell J, Davies A, Razay G. A community-based exercise programme to improve functional ability in people with Alzheimer's disease: A randomized controlled trial. Scand J Caring Sci. 2012;26:12–9. doi:10.1111/j.1471-6712.2011.00895.x.

61. Wesson J, Clemson L, Brodaty H, Lord S, Taylor M, Gitlin L, Close J. A feasibility study and pilot randomised trial of a tailored prevention program to reduce falls in older people with mild dementia. BMC Geriatr. 2013;13:89. doi:10.1186/1471-2318-13-89.

65. Burgener SC, Yang Y, Gilbert R, Marsh-Yant S. The effects of a multimodal intervention on outcomes of persons with early-stage dementia. Am. J. Alzheimers Dis. Other Dement. 2008;23:382–94. doi:10.1177/1533317508317527.

66. Christofoletti G, Oliani MM, Gobbi S, Stella F, Bucken Gobbi LT, Renato Canineu P. A controlled clinical trial on the effects of motor intervention on balance and cognition in institutionalized elderly patients with dementia. Clin Rehabil. 2008;22:618–26. doi:10.1177/0269215507086239.

67. Kim M-J, Han C-W, Min K-Y, Cho C-Y, Lee C-W, Ogawa Y, et al. Physical Exercise with Multicomponent Cognitive Intervention for Older Adults with Alzheimer's Disease: A 6-Month Randomized Controlled Trial. Dement Geriatr Cogn Dis Extra. 2016;6:222–32. doi:10.1159/000446508.

68. Lam FMH, Liao LR, Kwok TCY, Pang MYC. Effects of adding whole-body vibration to routine day activity program on physical functioning in elderly with mild or moderate dementia: A randomized controlled trial. Int J Geriatr Psychiatry. 2018;33:21–30. doi:10.1002/gps.4662.

69. Padala KP, Padala PR, Lensing SY, Dennis RA, Bopp MM, Roberson PK, Sullivan DH. Home-Based Exercise Program Improves Balance and Fear of Falling in Community-Dwelling Older Adults with Mild Alzheimer's Disease: A Pilot Study. J Alzheimers Dis. 2017;59:565–74. doi:10.3233/JAD-170120.

70. Padala KP, Padala PR, Malloy TR, Geske JA, Dubbert PM, Dennis RA, et al. Wii-fit for improving gait and balance in an assisted living facility: A pilot study. J Aging Res. 2012;2012:597573. doi:10.1155/2012/597573.

71. Telenius EW, Engedal K, Bergland A. Effect of a high-intensity exercise program on physical function and mental health in nursing home residents with dementia: An assessor blinded randomized controlled trial. PLoS One. 2015;10:e0126102. doi:10.1371/journal.pone.0126102.

72. Toots A, Littbrand H, Lindelöf N, Wiklund R, Holmberg H, Nordström P, et al. Effects of a High-Intensity Functional Exercise Program on Dependence in Activities of Daily Living and Balance in Older Adults with Dementia. J Am Geriatr Soc. 2016;64:55–64. doi:10.1111/jgs.13880.

73. Yoon JE, Lee SM, Lim HS, Kim TH, Jeon JK, Mun MH. The effects of cognitive activity combined with active extremity exercise on balance, walking activity, memory level and quality of life of an older adult sample with dementia. J. Phys. Ther. Sci. 2013;25:1601–4. doi:10.1589/jpts.25.1601.

74. Dawson N, Judge KS, Gerhart H. Improved Functional Performance in Individuals With Dementia After a Moderate-Intensity Home-Based Exercise Program: A Randomized Controlled Trial. J Geriatr Phys Ther. 2019;42:18–27. doi:10.1519/JPT.0000000000000128.

76. Francese T, Sorrell J, Butler FR. The effects of regular exercise on muscle strength and functional abilities of late stage Alzheimer's residents. Am J Alzheimers Dis Other Demen. 1997;12:122–7. doi:10.1177/153331759701200305.

77. Hauer K, Ullrich P, Dutzi I, Beurskens R, Kern S, Bauer J, Schwenk M. Effects of Standardized Home Training in Patients with Cognitive Impairment following Geriatric Rehabilitation: A Randomized Controlled Pilot Study. Gerontology. 2017;63:495–506. doi:10.1159/000478263.

78. Hauer K, Schwenk M, Zieschang T, Essig M, Becker C, Oster P. Physical training improves motor performance in people with dementia: A randomized controlled trial. J Am Geriatr Soc. 2012;60:8–15. doi:10.1111/j.1532-5415.2011.03778.x.

79. Kovács E, Sztruhár Jónásné I, Karóczi CK, Korpos A, Gondos T. Effects of a multimodal exercise program on balance, functional mobility and fall risk in older adults with cognitive impairment: A randomized controlled single-blind study. Eur J Phys Rehabil Med. 2013;49:639–48.

80. Santana-Sosa E, Barriopedro MI, López-Mojares LM, Pérez M, Lucia A. Exercise training is beneficial for Alzheimer's patients. Int J Sports Med. 2008;29:845–50. doi:10.1055/s-2008-1038432.

82. Cancela JM, Ayán C, Varela S, Seijo M. Effects of a long-term aerobic exercise intervention on institutionalized patients with dementia. J Sci Med Sport. 2016;19:293–8. doi:10.1016/j.jsams.2015.05.007.

83. Kampragkou C, Iakovidis P, Kampragkou E, Kellis E. Effects of a 12-Week Aerobic Exercise Program Combined with Music Therapy and Memory Exercises on Cognitive and Functional Ability in People with Middle Type of Alzheimer's Disease. International Journal of Physiotherapy 2017. doi:10.15621/ijphy/2017/v4i5/159420.

84. Sobol NA, Hoffmann K, Frederiksen KS, Vogel A, Vestergaard K, Brændgaard H, et al. Effect of aerobic exercise on physical performance in patients with Alzheimer's disease. Alzheimers Dement. 2016;12:1207–15. doi:10.1016/j.jalz.2016.05.004.

85. Toulotte C, Fabre C, Dangremont B, Lensel G, Thévenon A. Effects of physical training on the physical capacity of frail, demented patients with a history of falling: A randomised controlled trial. Age Ageing. 2003;32:67–73. doi:10.1093/ageing/32.1.67.

86. Aguiar P, Monteiro L, Feres A, Gomes I, Melo A. Rivastigmine transdermal patch and physical exercises for Alzheimer's disease: A randomized clinical trial. Curr Alzheimer Res. 2014;11:532–7. doi:10.2174/1567205011666140618102224.

89. Rolland Y, Pillard F, Klapouszczak A, Reynish E, Thomas D, Andrieu S, et al. Exercise program for nursing home residents with Alzheimer's disease: A 1-year randomized, controlled trial. J Am Geriatr Soc. 2007;55:158–65. doi:10.1111/j.1532-5415.2007.01035.x.

90. Souto Barreto P de, Cesari M, Denormandie P, Armaingaud D, Vellas B, Rolland Y. Exercise or Social Intervention for Nursing Home Residents with Dementia: A Pilot Randomized, Controlled Trial. J Am Geriatr Soc. 2017;65:E123-E129. doi:10.1111/jgs.14947.

91. Toots A, Littbrand H, Holmberg H, Nordström P, Lundin-Olsson L, Gustafson Y, Rosendahl E. Walking Aids Moderate Exercise Effects on Gait Speed in People With Dementia: A Randomized Controlled Trial. J Am Med Dir Assoc. 2017;18:227–33. doi:10.1016/j.jamda.2016.09.003.

93. Pedrinolla A, Venturelli M, Fonte C, Munari D, Benetti MV, Rudi D, et al. Exercise Training on Locomotion in Patients with Alzheimer's Disease: A Feasibility Study. J Alzheimers Dis. 2018;61:1599–609. doi:10.3233/JAD-170625.

94. Schwenk M, Dutzi I, Englert S, Micol W, Najafi B, Mohler J, Hauer K. An intensive exercise program improves motor performances in patients with dementia: Translational model of geriatric rehabilitation. J Alzheimers Dis. 2014;39:487–98. doi:10.3233/JAD-130470.

95. Schwenk M, Zieschang T, Englert S, Grewal G, Najafi B, Hauer K. Improvements in gait characteristics after intensive resistance and functional training in people with dementia: A randomised controlled trial. BMC Geriatr. 2014;14:73. doi:10.1186/1471-2318-14-73.

96. Kemoun G, Thibaud M, Roumagne N, Carette P, Albinet C, Toussaint L, et al. Effects of a physical training programme on cognitive function and walking efficiency in elderly persons with dementia. Dement Geriatr Cogn Disord. 2010;29:109–14. doi:10.1159/000272435.

98. Steinberg M, Leoutsakos J-MS, Podewils LJ, Lyketsos CG. Evaluation of a home-based exercise program in the treatment of Alzheimer’s disease: The Maximizing Independence in Dementia (MIND) study. Int J Geriatr Psychiatry. 2009;24:680–5. doi:10.1002/gps.2175.

100. Werner C, Wiloth S, Lemke NC, Kronbach F, Jansen C-P, Oster P, et al. People with Dementia Can Learn Compensatory Movement Maneuvers for the Sit-to-Stand Task: A Randomized Controlled Trial. J Alzheimers Dis. 2017;60:107–20. doi:10.3233/JAD-170258.

105. Roach KE, Tappen RM, Kirk-Sanchez N, Williams CL, Loewenstein D. A randomized controlled trial of an activity specific exercise program for individuals with Alzheimer disease in long-term care settings. J Geriatr Phys Ther. 2011;34:50–6. doi:10.1519/JPT.0b013e31820aab9c.

106. Tappen RM, Roach KE, Applegate EB, Stowell P. Effect of a combined walking and conversation intervention on functional mobility of nursing home residents with Alzheimer disease. Alzheimer Dis Assoc Disord. 2000;14:196–201. doi:10.1097/00002093-200010000-00002.

107. Venturelli M, Scarsini R, Schena F. Six-month walking program changes cognitive and ADL performance in patients with Alzheimer. Am J Alzheimers Dis Other Demen. 2011;26:381–8. doi:10.1177/1533317511418956.

109. Pitkälä KH, Pöysti MM, Laakkonen M-L, Tilvis RS, Savikko N, Kautiainen H, Strandberg TE. Effects of the Finnish Alzheimer disease exercise trial (FINALEX): A randomized controlled trial. JAMA Intern Med. 2013;173:894–901. doi:10.1001/jamainternmed.2013.359.

112. Bossers WJR, van der Woude LHV, Boersma F, Hortobágyi T, Scherder EJA, van Heuvelen MJG. Comparison of Effect of Two Exercise Programs on Activities of Daily Living in Individuals with Dementia: A 9-Week Randomized, Controlled Trial. J Am Geriatr Soc. 2016;64:1258–66. doi:10.1111/jgs.14160.

113. Henskens M, Nauta IM, Drost KT, Scherder EJ. The effects of movement stimulation on activities of daily living performance and quality of life in nursing home residents with dementia: A randomized controlled trial. Clin Interv Aging. 2018;13:805–17. doi:10.2147/CIA.S160031.

124. Cott CA, Dawson P, Sidani S, Wells D. The effects of a walking/talking program on communication, ambulation, and functional status in residents with Alzheimer disease. Alzheimer Dis Assoc Disord. 2002;16:81–7. doi:10.1097/00002093-200204000-00005.

125. Pomeroy VM, Warren CM, Honeycombe C, Briggs RS, Wilkinson DG, Pickering RM, Steiner A. Mobility and dementia: Is physiotherapy treatment during respite care effective? Int J Geriatr Psychiatry. 1999;14:389–97. doi:10.1002/(SICI)1099-1166(199905)14:5<389::AID-GPS933>3.0.CO;2-8.

126. Schwenk M, Zieschang T, Oster P, Hauer K. Dual-task performances can be improved in patients with dementia: A randomized controlled trial. Neurology. 2010;74:1961–8. doi:10.1212/WNL.0b013e3181e39696.
