# Supplementary material for: Recommended motor assessments based on psychometric properties in individuals with dementia: a systematic review
Source: Eur Rev Aging Phys Act. 2019 Nov 3;16:20. doi: 10.1186/s11556-019-0228-z (PMC6825725; doi:10.1186/s11556-019-0228-z)
Supplement: Supplementary file 8 — Additional file 8. Data extraction main search. [file 11556_2019_228_MOESM8_ESM.pdf]

Additional file 8

Data extraction main search

Content, construct, and criterion validity, internal consistency, and intra-rater reliability

Table S4. Data extraction main search - content, construct, and criterion validity, internal consistency, and intra-rater reliability

Motor Assessment	Outcome	Reference
	Construct validity	
**Physiomat-Trail-Making Task**	Hypotheses testing<br>1) moderate-to-high associations with MMSE: r=0.29-0.66, p≤0.001-0.004 → Yes<br>2) more pronounced associations with modified Trail-Making-Test A: r=0.36-0.83, p≤0.001 → Yes<br>3) moderate associations with memory tests: r=(-0.42)-(-0.16), p=0.004-0.12 → Yes<br>4) higher associations of cognitive outcome measures with increasing complexity: r=(-0.33)-0.36, p≤0.001-0.02 vs. r=(-0.42)-0.44, p≤0.001-0.12 vs. r=(-0.22)-0.83, p≤0.001-0.12 → Yes (repeating numbers)<br>5) associations with TUG and POMA: r=(-0.40)-0.48, p≤0.001-0.71 → Yes<br>6) pronounced associations with Physiomat-Follow-The-Ball Task: r=0.61-0.71, p≤0.001 → Yes<br>7) less association with moderate Physiomat-Balance-Task (10 seconds): r=(-0.34)-0.11, p=0.10-0.71 → Yes<br>8) higher associations of motor-functional outcomes with decreasing complexity: r=(-0.22)-0.22, p=0.004-0.03 vs. r=(-0.40)-0.48, p≤0.001 vs. r=0.08-0.19, p=0.35-0.71 → No	Wiloth et al., 2016 [53]
**Maximum isometric strength assessed with dynamometers** (knee extensor strength)	Mann-Whitney U-test/unpaired t-test, independent gait/STS performance vs. dependent gait/STS performance: p<0.0001	Suzuki et al., 2009 [114]
**E-ADL Test**	Hypotheses testing<br>Correlation with severity of dementia: r=(-0.47)-0.72, p≤0.001<br>Correlation with Nurses' Observations Scale for Geriatric Patients - instrumental activities of daily living/activities of daily living: r=(-0.45)-(-0.33), p≤0.001-0.023<br>Correlation with Nurses' Observations Scale for Geriatric Patients - mood/disturbing behaviour: r=(-0.40)-(-0.33), p=0.007-0.027<br>Correlation with Nurses' Observations Scale for Geriatric Patients - total score: r=-0.60, p<0.001	Graessel et al., 2009 [110]

Table S4. Data extraction main search - content, construct, and criterion validity, internal consistency, and intra-rater reliability *(Continued)*

Motor Assessment	Outcome	Reference
**E-ADL Test**	Hypotheses testing<br>Spearman correlation with cognition: r=0.39-0.43<br>Spearman correlation with everyday practical capabilities: r=0.39-0.64<br>Spearman correlation with mood/behaviour: r=0.11-0.39	Luttenberger et al., 2012 [111]
	Criterion validity (concurrent and predictive validity)/correlation with/prediction of external criteria	
**Modified BBS**	Bivariate correlation with spatiotemporal gait parameters: r=(-0.85)-0.73, n.s./$p<0.05$/$p<0.01$<br>Partial correlation with spatiotemporal gait parameters: r=(-0.67)-0.72, n.s./$p<0.05$/$p<0.01$	McGough et al., 2013 [26]
**POMA**	Mann-Whitney U-test, fallers vs. non-fallers: p=0.928<br>Univariate logistic regression analysis to predict risk of falling in the next three months: R^2=0.000, OR=1.002, CI_{95}=0.904-1.111, p=0.966	Schwenk et al., 2014 [117]
**TUG**	Mann-Whitney U-test: fallers vs. non-fallers: p=0.236<br>Univariate logistic regression analysis to predict risk of falling in the next three months: R^2=0.011, OR=0.966, CI_{95}=0.883-1.056, p=0.612	Schwenk et al., 2014 [117]
**5x STS**	Mann-Whitney U-test, fallers vs. non-fallers: p=0.553<br>Univariate logistic regression analysis to predict risk of falling in the next three months: R^2=0.005, OR=1.023, CI_{95}=0.937-1.118, p=0.966	Schwenk et al., 2014 [117]
**ACSID**	Correlation with 2D video-motion analysis: r=(-0.73)-0.84, $p<0.001$	Werner et al., 2018 [99]
**Maximum isometric strength assessed with dynamometers** (knee extensor strength)	Logistic regression analysis: knee extensor strength was a significant predictor of<br>- Gait performance (OR: 443.02, CI_{95}: 9.20-21325.69)<br>- STS performance (OR: 47.32, CI_{95}: 3.31-675.81)	Suzuki et al., 2009 [114]
	Chi^2 test/Mann-Whitney U-test/ unpaired t-test, independent activities of daily living/gait performance vs. dependent activities of daily living/gait performance: p≤0.0001<br>Logistic regression analysis: knee extensor strength muscles was a significant predictor of<br>- Dressing the lower body (OR: 109.90, CI_{95}: 7.60-1589.49)<br>- Toileting (OR: 18.29, CI_{95}: 2.41-138.84)<br>- Transfers to bed/toilet/shower (OR: 39.70, CI_{95}: 4.51-349.08)<br>- Gait performance (OR: 12.77, CI_{95}: 2.30-70.77)	Suzuki et al., 2012 [116]
**6min WT**	Pearson bivariate correlation with peak cycle ergometer test: r=0.33-0.51, $p<0.05$	Bronas et al., 2017 [115]

Table S4. Data extraction main search - content, construct, and criterion validity, internal consistency, and intra-rater reliability *(Continued)*

Motor Assessment	Outcome	Reference
**SPPB**	Bivariate correlation with spatiotemporal gait parameters: r=(-0.71)-0.66, n.s./p<0.01<br>Partial correlation with spatiotemporal gait parameters: r=(-0.65)-0.71, n.s./p<0.05/p<0.01	McGough et al., 2013 [26]
	Pearson bivariate correlation with peak cycle ergometer test: r=0.35, p<0.05	Bronas et al., 2017 [115]
**E-ADL Test**	Correlation with level of care: eta=0.39<br>Degree of level of care in relation to E-ADL score: eta=0.48<br>Kruskal-Wallis test: p<0.001 (df=2) for care level at baseline and after 22 months (df=3)<br>Mann-Whitney U-test, unchanged care level vs. increased care level: p=0.01, U=376, achieved power at p=0.01: 0.48	Luttenberger et al., 2012 [111]
	Internal consistency	
**BBS**	Cronbach's α=0.95<br>Item-to-total correlations: r>0.4 for all items except item 3, n.s./p<0.05/p<0.01	Telenius et al., 2015 [15]
**E-ADL Test**	Cronbach's α=0.77<br>Correlation between items: r=0.18-0.51, p<0.001-0.224	Graessel et al., 2009 [110]
	Cronbach's α=0.68 (total sample), α=0.37 (mild dementia), α=0.64 (moderate dementia), α=0.73 (severe dementia)<br>Correlation between the 5 items: r=0.21-0.44	Luttenberger et al., 2012 [111]
	Intra-rater reliability	
**ACSID**	Percentage agreement=90.2-100.0<br>Cohen's κ=0.77-0.91<br>ICC (CI_{95})=0.84 (0.76-0.89)	Werner et al., 2018 [99]

5x STS: Five Times Sit-to-Stand Test, 6min WT: 6-minute walk test, ACSID: Assessment of Compensatory Sit-to-Stand Maneuvers in People With Dementia, BBS: Berg Balance Scale, CI95: 95% confidence interval, E-ADL Test: Erlangen Test of Activities of Daily Living, ICC: intraclass correlation coefficient, MMSE: Mini-Mental-State Examination, n.s.: not significant, OR: odds ratio, POMA: Performance Oriented Mobility Assessment, SPPB: Short Physical Performance Battery, STS: Sit-to-Stand, TUG: Timed Up & Go Test

Inter-rater reliability

Table S5. Data extraction main search - inter-rater reliability

Motor assessment	Variable	Relative inter-rater reliability	Absolute inter-rater reliability	Reference
		Balance		
FR	Distance [cm]	ICC (CI_{95})=0.79 (0.43-0.94)	N/A	Muir-Hunter et al., 2015 [14]
GMWT	Time [s]	ICC=0.99	SEM=1.00<br>MDC_{95}=2.78<br>$MDC_{95\%}$=14.5%	Lee et al., 2017 [43]
	Number of oversteps	ICC=0.99	SEM=0.76<br>MDC_{95}=2.12<br>$MDC_{95\%}$=17.1%	Lee et al., 2017 [43]
BBS	Score	ICC=0.99	SEM=0.78<br>MDC_{95}=2.18<br>$MDC_{95\%}$=5.1%	Lee et al., 2017 [43]
		ICC (CI_{95})=0.72 (0.31-0.91)	N/A	Muir-Hunter et al., 2015 [14]
		Weighted κ=0.94<br>ICC=0.99	SEM=0.97<br>MDC_{95}=1.92<br>$MDC_{95\%}$=7.0%	Telenius et al., 2015 [15]
		Mobility and gait		
TUG	Time [s]	ICC=0.99	SEM=0.63<br>MDC_{95}=1.75<br>$MDC_{95\%}$=7.9%	Lee et al., 2017 [43]
		ICC (CI_{95})=0.98 (0.93-0.99)	N/A	Muir-Hunter et al., 2015 [14]
6m WT	Walking speed [m/s]	ICC=0.97	SEM=0.03<br>MDC_{95}=0.06<br>$MDC_{95\%}$=15.2%	Telenius et al., 2015 [15]

Table S5. Data extraction main search - inter-rater reliability *(Continued)*

Motor assessment	Variable	Relative inter-rater reliability	Absolute inter-rater reliability	Reference
4m WT	Walking speed [m/s]	ICC=0.82	SEM=0.74<br>MDC_{95}=2.06<br>$MDC_{95\%}$=98.0%	Lee et al., 2017 [43]
		Strength		
30s CST	Repetitions	ICC=1.00	SEM=0.00<br>MDC_{95}=0.00<br>$MDC_{95\%}$=0.0%	Telenius et al., 2015 [15]
ACSID	Score	Percentage agreement=92.1-100.0<br>Cohen's κ=0.64-0.99<br>ICC (CI_{95})=0.85 (0.78-0.90)	N/A	Werner et al., 2018 [99]
		Endurance		
6min WT	Distance [ft]	AM: ICC=0.99<br>PM: ICC=0.97	N/A	Tappen et al., 1997 [118]
	Walking speed [ft/s]	AM: ICC=0.98<br>PM: ICC=0.96	N/A	Tappen et al., 1997 [118]

4m WT: 4-metre walk test, 6m WT: 6-metre walk test, 6min WT: 6-minute walk test, 30s CST: 30-second chair stand test, ACSID: Assessment of Compensatory Sit-to-Stand Maneuvers in People With Dementia, AM: morning measures, BBS: Berg Balance Scale, CI_{95}: 95% confidence interval, FR: Functional Reach Test, GMWT: Groningen Meander Walking Test, ICC: intraclass correlation coefficient, MDC_{95}: minimal detectable changes at 95% confidence interval, $MDC_{95\%}$: percentage minimal detectable changes at 95% confidence interval, N/A: not applicable, PM: afternoon measures, SEM: standard error of measurement, TUG: Timed Up & Go Test

Test-retest reliability

Table S6. Data extraction main search - test-retest reliability

Motor assessment	Variable	Relative test-retest reliability	Absolute test-retest reliability	Reference
		Balance		
**FICSIT-4**	Score	ICC (CI_{95})=0.79 (0.67-0.87)	SEM (CI_{95})=0.55 (0.47-0.69)<br>MDC_{95}=1.52<br>$MDC_{95\%}$=59.4%	Blankenvoort et al., 2013 [17]
		ICC (CI_{95})=0.82 (0.65-0.91)	SEM (CI_{95})=0.59 (0.48-0.81)<br>MDC_{95}=1.64<br>$MDC_{95\%}$=58.9%	Blankenvoort et al., 2013 [17][SG1]
		ICC (CI_{95})=0.80 (0.61-0.90)	SEM (CI_{95})=0.60 (0.48-0.82)<br>MDC_{95}=1.66<br>$MDC_{95\%}$=71.1%	Blankenvoort et al., 2013 [17][SG2]
**Modified Clinical Test of Sensory Interaction of Balance**	Sway velocity [deg/s]	ICC=0.91	SEM=0.17<br>MDC_{95}=0.34<br>$MDC_{95\%}$=36.5%<br>CV=14.9%	Suttanon et al., 2011 [51]
**Limits of Stability**	Reaction time [s]	ICC=0.52	SEM=0.15<br>MDC_{95}=0.29<br>$MDC_{95\%}$=38.0%<br>CV=14.2%	Suttanon et al., 2011 [51]
	Movement velocity [deg/s]	ICC=0.48	SEM=0.46<br>MDC_{95}=0.91<br>$MDC_{95\%}$=38.9%<br>CV=14.7%	Suttanon et al., 2011 [51]
	Maximum excursion [%]	ICC=0.68	SEM=4.44<br>MDC_{95}=8.71<br>$MDC_{95\%}$=15.9%<br>CV=6.2%	Suttanon et al., 2011 [51]

Table S6. Data extraction main search - test-retest reliability *(Continued)*

Motor assessment	Variable	Relative test-retest reliability	Absolute test-retest reliability	Reference
**Limits of Stability**	Directional control [%]	ICC=0.71	SEM=5.24<br>MDC_{95}=10.27<br>$MDC_{95\%}$=21.8%<br>CV=8.3%	Suttanon et al., 2011 [51]
**Physiomat-Trail-Making Task**	Score	r_s=0.89<br>ICC (CI_{95})=0.90 (0.85-0.95)	N/A	Wiloth et al., 2016 [53]
**Physiomat-Trail-Making Task** simple	Sway path [mm/s]	r_s=0.59<br>ICC (CI_{95})=0.47 (0.27-0.63)	N/A	Wiloth et al., 2016 [53]
	Time [s]	r_s=0.60<br>ICC (CI_{95})=0.55 (0.37-0.69)	N/A	Wiloth et al., 2016 [53]
**Physiomat-Trail-Making Task** moderate	Sway path [mm/s]	r_s=0.78<br>ICC (CI_{95})=0.74 (0.61-0.82)	N/A	Wiloth et al., 2016 [53]
	Time [s]	r_s=0.74<br>ICC (CI_{95})=0.79 (0.68-0.87)	N/A	Wiloth et al., 2016 [53]
**Physiomat-Trail-Making Task** complex	Sway path [mm/s]	r_s=0.80<br>ICC (CI_{95})=0.82 (0.69-0.89)	N/A	Wiloth et al., 2016 [53]
	Time [s]	r_s=0.87<br>ICC (CI_{95})=0.83 (0.72-0.91)	N/A	Wiloth et al., 2016 [53]
**Physiomat-Follow-the-Ball Task**	Sway path [mm/s]	r_s=0.74<br>ICC (CI_{95})=0.84 (0.76-0.89)	N/A	Wiloth et al., 2016 [53]
	Duration [s]	r_s=0.69<br>ICC (CI_{95})=0.79 (0.68-0.86)	N/A	Wiloth et al., 2016 [53]
**FR**	Distance [cm]	ICC (CI_{95})=0.81 (0.52-0.94)	SEM=4.56<br>MDC_{95}=12.64<br>$MDC_{95\%}$=68.9%	Muir-Hunter et al., 2015 [14]

Table S6. Data extraction main search - test-retest reliability *(Continued)*

Motor assessment	Variable	Relative test-retest reliability	Absolute test-retest reliability	Reference
**FR**	Distance [cm]	ICC=0.84	SEM=1.61<br>MDC_{95}=3.15<br>$MDC_{95\%}$=15.4%<br>CV=5.7%	Suttanon et al., 2011 [51]
**Hill Step Test**	Number of steps (worst leg)	ICC=0.87	SEM=1.24<br>MCD_{95}=2.42<br>$MDC_{95\%}$=26.2%<br>CV(%)=11.3%	Suttanon et al., 2011 [51]
**Step Quick Turn**	Time [s]	ICC=0.55	SEM=0.33<br>MDC_{95}=0.64<br>$MDC_{95\%}$=38.1%<br>CV=14.4%	Suttanon et al., 2011 [51]
	Sway [deg/s]	ICC=0.64	SEM=4.56<br>MDC_{95}=8.93<br>$MDC_{95\%}$=29.7%<br>CV=10.5%	Suttanon et al., 2011 [51]
**Figure of Eight**	Time [s]	ICC (CI_{95})=0.91 (0.85-0.95)	SEM (CI_{95})=6.26 (5.41-8.21)<br>MDC_{95}=17.35<br>$MDC_{95\%}$=37.9%	Blankenvoort et al., 2013 [17]
		ICC (CI_{95})=0.94 (0.86-0.97)	SEM (CI_{95})=6.24 (5.63-10.03)<br>MDC_{95}=17.30<br>$MDC_{95\%}$=36.9%	Blankenvoort et al., 2013 [17][SG1]
		ICC (CI_{95})=0.85 (0.67-0.94)	SEM (CI_{95})=6.00 (4.01-7.58)<br>MDC_{95}=16.63<br>$MDC_{95\%}$=37.4%	Blankenvoort et al., 2013 [17][SG2]
**GMWT**	Time [s]	ICC (CI_{95})=0.94 (0.90-0.97)	SEM (CI_{95})=1.93 (1.64-2.54)<br>MDC_{95}=5.35<br>$MDC_{95\%}$=31.2%	Bossers et al., 2014 [63]

Table S6. Data extraction main search - test-retest reliability *(Continued)*

Motor assessment	Variable	Relative test-retest reliability	Absolute test-retest reliability	Reference
**GMWT**	Time [s]	ICC=0.96	n.r.	Bossers et al., 2014 [63][SG1]
		ICC=0.93	n.r.	Bossers et al., 2014 [63][SG2]
		ICC=0.99	SEM=1.36<br>MDC_{95}=3.78<br>$MDC_{95\%}$=19.6%	Lee et al., 2017 [43]
	Number of oversteps	ICC (CI_{95})=0.63 (0.41-0.78)	SEM (CI_{95})=1.58 (1.31-2.03)<br>MDC_{95}=4.38<br>$MDC_{95\%}$=225.7%	Bossers et al., 2014 [63]
		ICC=0.79	n.r.	Bossers et al., 2014 [63][SG1]
		ICC=0.57	n.r.	Bossers et al., 2014 [63][SG2]
		ICC=0.96	SEM=1.49<br>MDC_{95}=4.13<br>$MDC_{95\%}$=33.3%	Lee et al., 2017 [43]
**BBS**	Score	ICC=0.99	SEM=1.36<br>MDC_{95}=3.78<br>$MDC_{95\%}$=10.2%	Lee et al., 2017 [43]
		ICC (CI_{95})=0.95 (0.85-0.98)	SEM=6.01<br>MDC_{95}=16.66<br>$MDC_{95\%}$=38.6%	Muir-Hunter et al., 2015 [14]
		Mobility and gait		
**TUG**	Time [s]	ICC (CI_{95})=0.94 (0.92-0.97)	SEM (CI_{95})=2.12 (1.74-2.52)<br>MDC_{95}=5.88<br>$MDC_{95\%}$=31.6%	Blankenvoort et al., 2013 [17]
		ICC (CI_{95})=0.96 (0.92-0.98)	SEM (CI_{95})=1.43 (1.06-1.79)<br>MDC_{95}=3.96<br>$MDC_{95\%}$=23.3%	Blankenvoort et al., 2013 [17][SG1]

Table S6. Data extraction main search - test-retest reliability *(Continued)*

Motor assessment	Variable	Relative test-retest reliability	Absolute test-retest reliability	Reference
**TUG**	Time [s]	ICC (CI_{95})=0.94 (0.87-0.97)	SEM (CI_{95})=2.91 (2.10-3.61)<br>MDC_{95}=8.07<br>$MDC_{95\%}$=39.6%	Blankenvoort et al., 2013 [17][SG2]
		ICC=0.99	SEM=1.27<br>MDC_{95}=3.52<br>$MDC_{95\%}$=15.8%	Lee et al., 2017 [43]
		ICC=0.99	SEM=2.48<br>MDC_{90}=4.09<br>$MDC_{95\%}$=27.7%	Ries et al., 2009 [6]
		ICC=0.99	SEM=1.52<br>$MDC_{95\%}$=21.1%	Ries et al., 2009 [6][SG1/2]
		ICC=99	SEM=3.03<br>$MDC_{95\%}$=30.0%	Ries et al., 2009 [6][SG3]
		ICC (CI_{95})=0.72 (0.33-0.90)	SEM=1.24<br>MDC_{95}=3.44<br>$MDC_{95\%}$=20.3%	Muir-Hunter et al., 2015 [14]
		ICC=0.76	SEM=1.24<br>MDC_{95}=2.42<br>$MDC_{95\%}$=24.9%<br>CV=9.4%	Suttanon et al., 2011 [51]
		ICC=0.87	N/A	Thomas et al., 2002 [102]
**Cognitive TUG**	Time [s]	ICC=0.51	SEM=2.39<br>MDC_{95}=4.69<br>$MDC_{95\%}$=36.2%<br>CV=14.1%	Suttanon et al., 2011 [51]

Table S6. Data extraction main search - test-retest reliability *(Continued)*

Motor assessment	Variable	Relative test-retest reliability	Absolute test-retest reliability	Reference
**Manual TUG**	Time [s]	ICC=0.70	SEM=1.45<br>MDC_{95}=2.83<br>$MDC_{95\%}$=26.7%<br>CV(%)=10.1%	Suttanon et al., 2011 [51]
**6m WT** (comfortable pace)	Walking speed [m/s]	ICC (CI_{95})=0.86 (0.78-0.92)	SEM (CI_{95})=0.10 (0.08-0.12)<br>MDC_{95}=0.27<br>$MDC_{95\%}$=36.5%	Blankenvoort et al., 2013 [17]
		ICC (CI_{95})=0.83 (0.67-0.91)	SEM (CI_{95})=0.11 (0.09-0.11)<br>MDC_{95}=0.29<br>$MDC_{95\%}$=41.5%	Blankenvoort et al., 2013 [17][SG1]
		ICC (CI_{95})=0.89 (0.78-0.95)	SEM (CI_{95})=0.09 (0.07-0.13)<br>MDC_{95}=0.25<br>$MDC_{95\%}$=31.6%	Blankenvoort et al., 2013 [17][SG2]
	Time [s]	ICC=0.92	N/A	Thomas et al., 2002 [102]
	Number of steps	ICC=0.80	N/A	Thomas et al., 2002 [102]
**6m WT** (fast pace)	Time [s]	ICC=0.95	N/A	Thomas et al., 2002 [102]
	Number of steps	ICC=0.90	N/A	Thomas et al., 2002 [102]
**4m WT**	Walking speed [m/s]	ICC=0.85	SEM=0.64<br>MDC_{95}=1.78<br>$MDC_{95\%}$=84.3%	Lee et al., 2017 [43]
**Gait analysis** (GAITRite)	Walking speed [m/s]	ICC (CI_{95})=0.95 (0.81-0.99)	N/A	McGough et al., 2013 [26]
	Walking speed [cm/s]	ICC=0.98	SEM=5.72<br>MDC_{90}=9.44<br>$MDC_{95\%}$=27.4%	Ries et al., 2009 [6]
		ICC=0.97-0.98*	SEM=6.07<br>$MDC_{95\%}$=25.5%	Ries et al., 2009 [6][SG1/2]
		ICC=0.97-0.98*	SEM=5.48<br>$MDC_{95\%}$=29.0%	Ries et al., 2009 [6][SG3]

Table S6. Data extraction main search - test-retest reliability *(Continued)*

Motor assessment	Variable	Relative test-retest reliability	Absolute test-retest reliability	Reference
**Gait analysis** (GAITRite)	Walking speed [m/s]	ICC (CI_{95})=0.95 (0.88–0.98)	MCD_{95}=0.13<br>$MDC_{95\%}$=12.0%<br>CV(%)=4.2%	Wittwer et al., 2008 [121][3 walks]
		ICC (CI_{95})=0.96 (0.91–0.99)	MCD_{95}=0.11<br>$MDC_{95\%}$=10.2%<br>CV=3.8%	Wittwer et al., 2008 [121][10 walks]
	Step length [cm]	ICC (CI_{95})=0.97 (0.93-0.99)	MCD_{95}=5.27<br>$MDC_{95\%}$=8.9%<br>CV=3.1%	Wittwer et al., 2008 [121][3 walks, r]
		ICC (CI_{95})=0.98 (0.96-0.99)	MCD_{95}=4.15<br>$MDC_{95\%}$=7.0%<br>CV=2.5%	Wittwer et al., 2008 [121][10 walks, r]
	Step width [cm]	ICC (CI_{95})=0.92 (0.82-0.97)	MCD_{95}=2.23<br>$MDC_{95\%}$=24.7%<br>CV=8.9%	Wittwer et al., 2008 [121][3 walks, r]
		ICC (CI_{95})=0.95 (0.87-0.98)	MCD_{95}=1.83<br>$MDC_{95\%}$=20.0%<br>CV=7.0%	Wittwer et al., 2008 [121][10 walks, r]
	Stride length (cm)	ICC (CI_{95})=0.97 (0.87-0.99)	N/A	McGough et al., 2013 [26]
		ICC (CI_{95})=0.97 (0.93-0.99)	MCD_{95}=10.24<br>$MDC_{95\%}$=8.5%<br>CV=3.0%	Wittwer et al., 2008 [121][3 walks, r]
		ICC (CI_{95})=0.98 (0.96-0.99)	MCD_{95}=8.12<br>$MDC_{95\%}$=6.8%<br>CV=2.4%	Wittwer et al., 2008 [121][10 walks, r]
	Cadence [steps/ min]	ICC (CI_{95})=0.91 (0.62-0.98)	N/A	McGough et al., 2013 [26]

Table S6. Data extraction main search - test-retest reliability *(Continued)*

Motor assessment	Variable	Relative test-retest reliability	Absolute test-retest reliability	Reference
**Gait analysis** (GAITRite)	Cadence [steps/ min]	ICC (CI_{95})=0.88 (0.72–0.95)	MCD_{95}=8.13<br>$MDC_{95\%}$=7.5%<br>CV=2.7%	Wittwer et al., 2008 [121] 3 walks
		ICC (CI_{95})=0.89 (0.74–0.95)	MDC_{95}=7.64<br>$MDC_{95\%}$=7.1%<br>CV=2.5%	Wittwer et al., 2008 [121] 10 walks
	Swing time [s]	ICC (CI_{95})=0.96 (0.81-0.99)	N/A	McGough et al., 2013 [26]
		ICC (CI_{95})=0.90 (0.76–0.96)	MCD_{95}=0.03<br>$MDC_{95\%}$=7.1%<br>CV=2.7%	Wittwer et al., 2008 [121] 3 walks, r
		ICC (CI_{95})=0.89 (0.75–0.96)	MCD_{95}=0.03<br>$MDC_{95\%}$=7.0%<br>CV=2.8%	Wittwer et al., 2008 [121] 10 walks, r
	Stance time [s]	ICC (CI_{95})=0.87 (0.70-0.95)	MCD_{95}=0.06<br>$MDC_{95\%}$=8.7%<br>CV=3.3%	Wittwer et al., 2008 [121] 3 walks, r
		ICC (CI_{95})=0.88 (0.73-0.95)	MCD_{95}=0.06<br>$MDC_{95\%}$=8.6%<br>CV=2.9%	Wittwer et al., 2008 [121] 10 walks, r
	Toe in/out angle [deg]	ICC (CI_{95})=0.91 (0.78-0.96)	MCD_{95}=3.06<br>$MDC_{95\%}$=33.5%<br>CV=12.9%	Wittwer et al., 2008 [121] 3 walks, r
		ICC (CI_{95})=0.93 (0.82-0.97)	MCD_{95}=2.58<br>$MDC_{95\%}$=28.2%<br>CV=10.8%	Wittwer et al., 2008 [121] 10 walks, r
	Walking speed variability [%]	ICC (CI_{95})=0.66 (0.26-0.87)	SEM=1.60<br>MDC_{95}=4.40<br>$MDC_{95\%}$=77.8%	Wittwer et al., 2013 [5]

Table S6. Data extraction main search - test-retest reliability *(Continued)*

Motor assessment	Variable	Relative test-retest reliability	Absolute test-retest reliability	Reference
**Gait analysis** (GAITRite)	Stride length variability [%]	ICC (CI_{95})=0.80 (0.52-0.93)	SEM=1.10<br>MDC_{95}=3.10<br>$MDC_{95\%}$=71.7%	Wittwer et al., 2013 [5]
	Stride width variability [%]	ICC (CI_{95})=0.83 (0.59-0.94)	SEM=3.00<br>MDC_{95}=8.30<br>$MDC_{95\%}$=47.0%	Wittwer et al., 2013 [5]
	Cadence variability [%]	ICC (CI_{95})=0.65 (0.25-0.86)	SEM=0.80<br>MDC_{95}=2.30<br>$MDC_{95\%}$=41.4%	Wittwer et al., 2013 [5]
**Gait analysis** (NeuroCom Balance Master)	Walking speed [cm/s]	ICC=0.50	SEM=7.58<br>MDC_{95}=14.86<br>$MDC_{95\%}$=48.3%<br>CV=20.6%	Suttanon et al., 2011 [51]
	Step length [cm]	ICC=0.75	SEM=4.59<br>MDC_{95}=9.00<br>$MDC_{95\%}$=35.6%<br>CV=13.9%	Suttanon et al., 2011 [51]
	Step width [cm]	ICC=0.89	SEM=1.26<br>MDC_{95}=2.48<br>$MDC_{95\%}$=22.0%<br>CV=14.7%	Suttanon et al., 2011 [51]
		Strength		
**5x STS**	Time [s]	ICC=0.80	SEM=1.39<br>MCD_{95}=2.73<br>$MDC_{95\%}$=29.9%<br>CV(%)=10.5%	Suttanon et al., 2011 [51]
		ICC=0.94	N/A	Thomas et al., 2002 [102]

Table S6. Data extraction main search - test-retest reliability *(Continued)*

Motor assessment	Variable	Relative test-retest reliability	Absolute test-retest reliability	Reference
**STS on NeuroCom Balance Master**	Rising index [% body weight]	ICC=0.95	SEM=1.25<br>MCD_{95}=2.44<br>$MDC_{95\%}$=21.8%<br>CV=7.7%	Suttanon et al., 2011 [51]
	COG sway velocity [deg/s]	ICC=0.02	SEM=1.20<br>MCD_{95}=2.35<br>$MDC_{95\%}$=80.2%<br>CV=39.2%	Suttanon et al., 2011 [51]
**Modified 30s CST**	Repetitions	ICC (CI_{95})=0.84 (0.73-0.90)	SEM (CI_{95})=1.26 (1.06-1.57)<br>MDC_{95}=3.49<br>$MDC_{95\%}$=42.5%	Blankenvoort et al., 2013 [17]
		ICC (CI_{95})=0.79 (0.60-0.90)	SEM (CI_{95})=1.52 (1.22-2.08)<br>MDC_{95}=4.21<br>$MDC_{95\%}$=45.7%	Blankenvoort et al., 2013 [17][SG1]
		ICC (CI_{95})=0.88 (0.73-0.95)	SEM (CI_{95})=0.83 (0.65-1.04)<br>MDC_{95}=2.30<br>$MDC_{95\%}$=33.2%	Blankenvoort et al., 2013 [17][SG2]
**Handgrip dynamometer**	Force [kgf]	ICC=0.98	N/A	Alencar et al., 2012 [120][SG0]
		ICC=0.97	N/A	Alencar et al., 2012 [120][SG1]
		ICC=0.96	N/A	Alencar et al., 2012 [120][SG2]
		ICC=0.42	N/A	Alencar et al., 2012 [120][SG3]
	Force [kg]	ICC (CI_{95})=0.90 (0.84-0.94)	SEM (CI_{95})=2.74 (2.05-2.98)<br>MDC_{95}=7.59<br>$MDC_{95\%}$=36.8%	Blankenvoort et al., 2013 [17]
		ICC (CI_{95})=0.86 (0.72-0.93)	SEM (CI_{95})=2.75 (1.85-3.15)<br>MDC_{95}=7.62<br>$MDC_{95\%}$=36.5%	Blankenvoort et al., 2013 [17][SG1]

Table S6. Data extraction main search - test-retest reliability *(Continued)*

Motor assessment	Variable	Relative test-retest reliability	Absolute test-retest reliability	Reference
**Handgrip dynamometer**	Force [kg]	ICC (CI_{95})=0.94 (0.87-0.97)	SEM (CI_{95})=2.57 (2.02-3.47)<br>MDC_{95}=7.11<br>$MDC_{95\%}$=34.9%	Blankenvoort et al., 2013 [17][SG2]
		Right: ICC=0.68<br>Left: ICC=0.70	N/A	Thomas et al., 2002 [102]
**Maximal isometric strength assessed with dynamometers**<br>(knee extension strength)	Torque [Nm]/normalized torque [Nm/kg]	ICC=0.97		Suzuki et al., 2009 [114]
		ICC=0.98	N/A	Suzuki et al., 2009 [114][SG1/2]
		ICC=0.95	N/A	Suzuki et al., 2009 [114][SG3]
	Peak force [kgf]	Right: ICC=0.63<br>Left: ICC=0.56	N/A	Thomas et al., 2002 [102]
**Maximal isometric strength assessed with dynamometers**<br>(hip flexor strength)	Peak force [kgf]	Right: ICC=0.71<br>Left: ICC=0.62	N/A	Thomas et al., 2002 [102]
**Maximal isometric strength assessed with dynamometers**<br>(dorsiflexor muscles strength)	Peak force [kgf]	Right: ICC=0.63<br>Left: ICC=0.77	N/A	Thomas et al., 2002 [102]
		Endurance		
**6min WT**	Distance [m]	ICC=0.99	SEM=20.28<br>MDC_{90}=33.47<br>$MDC_{95\%}$=23.9%	Ries et al., 2009 [6]
		ICC=0.98-0.99*	SEM=21.86<br>$MDC_{95\%}$=21.2%	Ries et al., 2009 [6][SG1/2]
		ICC=0.98-0.99*	SEM=19.57<br>$MDC_{95\%}$=28.9%	Ries et al., 2009 [6][SG3]

Table S6. Data extraction main search - test-retest reliability *(Continued)*

Motor assessment	Variable	Relative test-retest reliability	Absolute test-retest reliability	Reference
**6min WT**	Distance [ft]	Examiner 1, week 1: ICC=0.90<br>Examiner 1, week 2: ICC=0.80<br>Examiner 2, week 2: ICC=0.84	N/A	Tappen et al., 1997 [118]
		AM: ICC=0.84<br>PM: ICC=0.76	N/A	Tappen et al., 1997 [118]
	Walking speed [ft/s]	Examiner 1, week 1: ICC=0.89<br>Examiner 1, week 2: ICC=0.79<br>Examiner 2, week 2: ICC=0.84	N/A	Tappen et al., 1997 [118]
		AM: ICC=0.84<br>PM: ICC=0.75	N/A	Tappen et al., 1997 [118]
		Functional performance		
**E-ADL Test**	Score	r=0.73<br>(items: r=0.35-0.63)	N/A	Graessel et al., 2009 [110]

4m WT: 4-metre walk test, 5x STS: Five Times Sit-to-Stand Test, 6m WT: 6-metre walk test, 6min WT: 6-minute walk test, 30s CST: 30-second chair stand test, AM: morning measures, BBS: Berg Balance Scale, CI_{95}: 95% confidence interval, COG: centre of gravity, CV: coefficient of variation, E-ADL Test: Erlangen Test of Activities of Daily Living, FICSIT-4: Frailty and Injuries: Cooperative Studies of Intervention Techniques - subtest 4, FR: Functional Reach Test, GMWT: Groningen Meander Walking Test, ICC: intraclass correlation coefficient, kgf: kilogram-force, MDC_{90}: minimal detectable changes at 90% confidence interval, MDC_{95}: minimal detectable changes at 95% confidence interval, MDC_{95}%: percentage minimal detectable changes at 95% confidence interval, N/A: not applicable, n.r.: not reported, PM: afternoon measures, SEM: standard error of measurement, SG: subgroup, STS: Sit-to-Stand, TUG: Timed Up & Go Test

* range of ICC for several subgroups, no exact ICC reported

References

5. Wittwer JE, Webster KE, Hill K. Reproducibility of gait variability measures in people with Alzheimer's disease. Gait Posture. 2013;38:507–10. doi:10.1016/j.gaitpost.2013.01.021.

6. Ries JD, Echternach JL, Nof L, Gagnon Blodgett M. Test-retest reliability and minimal detectable change scores for the timed "up & go" test, the six-minute walk test, and gait speed in people with Alzheimer disease. Phys Ther. 2009;89:569–79. doi:10.2522/ptj.20080258.

14. Muir-Hunter SW, Graham L, Montero Odasso M. Reliability of the Berg Balance Scale as a Clinical Measure of Balance in Community-Dwelling Older Adults with Mild to Moderate Alzheimer Disease: A Pilot Study. Physiother. Can. 2015;67:255–62. doi:10.3138/ptc.2014-32.

15. Telenius EW, Engedal K, Bergland A. Inter-rater reliability of the Berg Balance Scale, 30 s chair stand test and 6 m walking test, and construct validity of the Berg Balance Scale in nursing home residents with mild-to-moderate dementia. BMJ Open. 2015;5:e008321. doi:10.1136/bmjopen-2015-008321.

17. Blankevoort CG, van Heuvelen MJG, Scherder EJA. Reliability of six physical performance tests in older people with dementia. Phys Ther. 2013;93:69–78. doi:10.2522/ptj.20110164.

26. McGough EL, Logsdon RG, Kelly VE, Teri L. Functional mobility limitations and falls in assisted living residents with dementia: Physical performance assessment and quantitative gait analysis. J Geriatr Phys Ther. 2013;36:78–86. doi:10.1519/JPT.0b013e318268de7f.

43. Lee HS, Park SW, Chung HK. The Korean version of relative and absolute reliability of gait and balance assessment tools for patients with dementia in day care center and nursing home. J. Phys. Ther. Sci. 2017;29:1934–9. doi:10.1589/jpts.29.1934.

51. Suttanon P, Hill KD, Dodd KJ, Said CM. Retest reliability of balance and mobility measurements in people with mild to moderate Alzheimer's disease. Int Psychogeriatr. 2011;23:1152–9. doi:10.1017/S1041610211000639.

53. Wiloth S, Lemke N, Werner C, Hauer K. Validation of a Computerized, Game-based Assessment Strategy to Measure Training Effects on Motor-Cognitive Functions in People With Dementia. JMIR Serious Games. 2016;4:e12. doi:10.2196/games.5696.

63. Bossers WJR, van der Woude LHV, Boersma F, Scherder EJA, van Heuvelen MJG. The Groningen Meander Walking Test: A dynamic walking test for older adults with dementia. Phys Ther. 2014;94:262–72. doi:10.2522/ptj.20130077.

99. Werner C, Wiloth S, Lemke NC, Kronbach F, Hauer K. Development and Validation of a Novel Motor-Cognitive Assessment Strategy of Compensatory Sit-to-Stand Maneuvers in People With Dementia. J Geriatr Phys Ther. 2018;41:143–54. doi:10.1519/JPT.0000000000000116.

102. Thomas VS, Hageman PA. A preliminary study on the reliability of physical performance measures in older day-care center clients with dementia. Int. Psychogeriatr. 2002;14:17–23. doi:10.1017/S1041610202008244.

110. Graessel E, Viegas R, Stemmer R, Küchly B, Kornhuber J, Donath C. The Erlangen Test of Activities of Daily Living: First results on reliability and validity of a short performance test to measure fundamental activities of daily living in dementia patients. Int. Psychogeriatr. 2009;21:103–12. doi:10.1017/S1041610208007710.

111. Luttenberger K, Schmiedeberg A, Gräßel E. Activities of daily living in dementia: Revalidation of the E-ADL Test and suggestions for further development. BMC Psychiatry. 2012;12:208. doi:10.1186/1471-244X-12-208.

114. Suzuki M, Yamada S, Inamura A, Omori Y, Kirimoto H, Sugimura S, Miyamoto M. Reliability and validity of measurements of knee extension strength obtained from nursing home residents with dementia. Am J Phys Med Rehabil. 2009;88:924–33. doi:10.1097/PHM.0b013e3181ae1003.

115. Bronas UG, Salisbury D, Kelly K, Leon A, Chow L, Yu F. Determination of Aerobic Capacity via Cycle Ergometer Exercise Testing in Alzheimer's Disease. Am J Alzheimers Dis Other Demen. 2017;32:500–8. doi:10.1177/1533317517720065.

116. Suzuki M, Kirimoto H, Inamura A, Yagi M, Omori Y, Yamada S. The relationship between knee extension strength and lower extremity functions in nursing home residents with dementia. Disabil. Rehabil. 2012;34:202–9. doi:10.3109/09638288.2011.593678.

117. Schwenk M, Hauer K, Zieschang T, Englert S, Mohler J, Najafi B. Sensor-derived physical activity parameters can predict future falls in people with dementia. Gerontology. 2014;60:483–92. doi:10.1159/000363136.

118. Tappen RM, Roach KE, Buchner D, Barry C, Edelstein J. Reliability of Physical Performance Measures in Nursing Home Residents With Alzheimer's Disease. J. Gerontol. Ser. A-Biol. Sci. Med. Sci. 1997;52A:M52-M55. doi:10.1093/gerona/52A.1.M52.

120. Alencar MA, Dias JMD, Figueiredo LC, Dias RC. Handgrip strength in elderly with dementia: Study of reliability. Rev Bras Fisioter. 2012;16:510–4. doi:10.1590/S1413-35552012005000059.

121. Wittwer JE, Webster KE, Andrews PT, Menz HB. Test-retest reliability of spatial and temporal gait parameters of people with Alzheimer's disease. Gait Posture. 2008;28:392–6. doi:10.1016/j.gaitpost.2008.01.007.
