# Supplementary material for: Recommended motor assessments based on psychometric properties in individuals with dementia: a systematic review
Source: Eur Rev Aging Phys Act. 2019 Nov 3;16:20. doi: 10.1186/s11556-019-0228-z (PMC6825725; doi:10.1186/s11556-019-0228-z)
Supplement: Supplementary file 9 — Additional file 9. Description, frequency of use, and effect sizes of motor assessments applied in previous randomised controlled trials without available information on psychometric properties. [file 11556_2019_228_MOESM9_ESM.pdf]

Additional file 9

Description, frequency of use, and effect sizes of motor assessments applied in previous randomised controlled trials without available information on psychometric properties

Table S7. Description, frequency of use, and effect sizes of motor assessments applied in randomised controlled trials (no psychometric properties investigations available)

Motor Assessment	Description	Frequency of use	Time*group interaction effect size
	Balance		
**Near-tandem test**	*Task*: adopting near-tandem position with eyes closed (not exactly specified)<br>*Measurement*: not specified	1 RCT (n=19) [61]	-
**Single leg stance/One Leg Standing Balance Test** [127]	*Task*: standing on a single leg alternately for 60/30 seconds with both eyes open and closed<br>*Measurement*: time [s]	2 RCT (n=63) [65, 83]	Large [c]
**One-leg balance test** [128]	*Task*: standing on one leg unsupported for five seconds (preferred leg)<br>*Measurement*: recording if participant is able to maintain one-leg stance for five seconds [≥5s: normal, <5s: abnormal]	1 RCT (n=110) [89]	-
**Inertial sensors assessing postural sway** [129]	*Task*: standing quietly with feet together for 30 seconds, while wearing an inertial sensor (DynaPort)<br>*Measurement*: sway area [sq cm]	1 RCT (n=81) [94]	Small/medium [r]
**Posturography platforms assessing postural sway**	*Task*: standing quietly on a posturography platform (QFP) for 51.2 seconds with eyes open<br>*Measurement*: elliptical area covered by moving centre of gravity [mm²]	1 RCT (n=20) [85]	-
**Wii Balance Board assessing postural sway** [130]	*Task*: standing quietly on a Wii Balance Board for 15/30 seconds under four conditions: feet apart with eyes open and closed, feet together with eyes open and closed<br>*Measurement*: centre or pressure velocity [cm/s], centre of pressure path length [cm/s]	1 RCT (n=20) [73]	-

Table S7. Description, frequency of use, and effect sizes of motor assessments applied in randomised controlled trials (no psychometric properties investigations available) *(Continued)*

Motor Assessment	Description	Frequency of use	Time*group interaction effect size
	Mobility and gait		
**Get-Up and Go Test** [131]	*Task*: standing up from a chair, walking three metres, turning around, walking back to the chair, and sitting down, use of a walking aid is allowed<br>*Measurement*: score from 1 to 5 [1=no instability to 5=very abnormal]	1 RCT (n=110) [89]	-
**10-meter walk test** [88]	*Task*: walking ten metres with comfortable pace, use of walking aid is allowed<br>*Measurement*: walking speed [m/s], time [s]	2 RCT (n=209) [84, 98]	-
**10-meter walk test with dual Task** [88]	*Task*: walking ten metres with comfortable pace while naming month backwards/counting backwards from 50, use of walking aid is allowed<br>*Measurement*: walking speed [m/s]	1 RCT (n=189) [84]	-
**8-foot walk test** [88]	*Task*: walking eight foot with comfortable/fast pace, use of walking aid is allowed<br>*Measurement*: walking speed [m/s], time [s]	3 RCT (n=78) [74, 77, 98]	Medium [r]
**Gait analysis with dual task performance** [92]	*Task*: walking with comfortable pace over an electronic walkway (GAITRite) while counting forward by twos/backward by threes<br>*Measurement*: dual-task cost in motor performance [%] for walking speed, cadence, stride length, stride time, single support, dual-task cost in combined performance [%]	1 RCT (n=49) [126]	-
**Southampton Assessment of Mobility** [132]	*Task*: different tasks of mobility (sit-to-stand, standing balance, gait, stand-to-sit)<br>*Measurement*: score [0=immobile, 18=able to walk four steps]	1 RCT (n=78) [125]	Small [c]
**Hierarchical Assessment of Balance and Mobility** [133]	*Task*: getting up from bedside and walking, which is rated in three areas: in-bed mobility, transfers, and walking<br>*Measurement*: score [0-65]	1 RCT (n=123) [94]	Small [r]
**Acute Care Index of Function** [134]	*Task*: 20 items that are divided into four subscales - mental status, bed mobility, transfers, and mobility<br>*Measurement*: score [0-1]	1 RCT (n=82) [105]	-

Table S7. Description, frequency of use, and effect sizes of motor assessments applied in randomised controlled trials (no psychometric properties investigations available) *(Continued)*

Motor Assessment	Description	Frequency of use	Time*group interaction effect size
	Strength		
**Body-fixed-sensor-based Sit-to-Stand analysis using DynaPort** [40]	*Task*: performing five repetitions of the sit-to-stand task without upper extremity assistance<br>*Measurement*: total duration [s], duration of hip extension/flexion [s], maximum angular velocity during hip extension/flexion [deg/s] of stand-to-sit and sit-to-stand movements and trunk flexion range [deg], trunk flexion duration [s], maximum trunk flexion angular velocity [deg/s], sit-to-stand movement duration	2 RCT (n=106) [77, 100]	No to large [c/r]
**10-s chair-stand test** [135]	*Task*: performing as many repetitions of sit-to-stand task as possible in 10 seconds without upper extremity assistance<br>*Measurement*: number of repetitions	1 RCT (n=40) [59]	Large [c]
**One-repetition maximum assessed with fitness machines**	*Task*: one-repetition maximum as achieved in the leg-press training machine for maximum dynamic concentric muscle strength in hip and knee extensors and in the abductor training machine for maximum strength in hip abductors, respectively<br>*Measurement*: one-repetition maximum [kg]	2 RCT (n=232) [78, 94]	Medium/large [r]
**Stair-climbing performance** [136]	*Task*: climbing a flight with thirteen stairs<br>*Measurement*: time [s]	1 RCT (n=107) [78]	-
**Physical therapy assessment**	*Task*: measure muscle strength in upper and lower extremities (not exactly specified)<br>*Measurement*: score [not specified]	1 RCT (n=11) [76]	-
	Endurance		
**2-min walk test** [137]	*Task*: walking for two minutes with comfortable pace, use of usual walking aids is allowed<br>*Measurement*: distance [m]	2 RCT (n=152) [124, 125]	-
**400-m walk test** [138]	*Task*: walking 400m with fast pace<br>*Measurement*: time [s]	1 RCT (n=189) [84]	-
**3-speed walking test**	*Task*: walking on a treadmill with 80%, 100%, and 120% of self-selected pace for five minutes<br>*Measurement*: cost of walking [J/kg/m], VO_2 [ml/kg/min], heart rate [bpm]	1 RCT (n=34) [93]	Medium to large [c]
**6-min Astrand Cycle Ergometer test** [139]	*Task*: submaximal 6-minute cycle test<br>*Measurement*: estimated maximum oxygen uptake [ml/kg/min]	1 RCT (n=189) [84]	-

Table S7. Description, frequency of use, and effect sizes of motor assessments applied in randomised controlled trials (no psychometric properties investigations available) *(Continued)*

**Motor Assessment**	**Description**	**Frequency of use**	**Time*group interaction effect size**
**Pedal Power**	*Task*: pedalling in seven steps from 10 to 70 W (not exactly specified)<br>*Measurement*: exercise time [s], pedal rotations [number], total load [W/s]	1 RCT (n=31) [67]	-
	Flexibility		
**Chair sit and reach** [140]	*Task*: stretching one leg keeping heel on the floor and trying to touch the toes with the fingers while sitting on a chair<br>*Measurement*: distance between the fingers and toes [cm]	1 RCT (n=20) [85]	-
	Functional performance		
**Physical Performance Test** [136]	*Task*: seven items (writing a sentence, transferring five beans from a bowl to a cup with a teaspoon, lifting a book onto a shelf, putting on a coat, picking up a coin from the floor, walking 50 feet, and turning 360° while standing in one place), which are scored on a 4-point Likert scale<br>*Measurement*: score [0-28]	1 RCT (n=105) [112]	-
**Senior Fitness Test** [141]	*Task*: battery of tests including:<br>(1) muscle dynamic strength endurance of legs (30-s chair stand test) and upper body (arm curl test),<br>(2) flexibility of lower (chair sit-and-reach test) and upper body (back scratch test),<br>(3) speed, agility and balance while moving (8-foot up-and-go test),<br>(4) aerobic endurance (2-minute step test)<br>*Measurement*:<br>(1) number of repetitions,<br>(2) distance between toes and fingers / fingers [cm],<br>(3) time [s],<br>(4) number of repetitions	1 RCT (n=16) [80]	Large [c/r]
**Jebsen Total Time** [142]	*Task*: performing a range of seven hand functions required for activities of daily living (writing, turning over 3 by 5 inch cards, picking up small common objects, simulated feeding, stacking checkers; picking up large objects, and picking up large heavy objects), while total time is evaluated<br>*Measurement*: total time [s]	1 RCT (n=27) [98]	-

Table S7. Description, frequency of use, and effect sizes of motor assessments applied in randomised controlled trials (no psychometric properties investigations available) *(Continued)*

Motor Assessment	Description	Frequency of use	Time*group interaction effect size
**Physiological Profile Assessment** [143]	*Task*: five items evaluating visual contrast sensitivity, knee joint proprioception, quadriceps strength, simple reaction time, and postural sway while standing on a foam rubber mat with eyes open<br>*Measurement*: fall risk score [z-score]	2 RCT (n=60) [52, 61]	-

n: number of analysed participants, RCT: randomised controlled trial/s

[c] calculated effect size, [r] effect size provided of randomised controlled trial

References

40. Schwenk M, Gogulla S, Englert S, Czempik A, Hauer K. Test-retest reliability and minimal detectable change of repeated sit-to-stand analysis using one body fixed sensor in geriatric patients. Physiol Meas. 2012;33:1931–46. doi:10.1088/0967-3334/33/11/1931.

52. Suttanon P, Hill KD, Said CM, Williams SB, Byrne KN, LoGiudice D, et al. Feasibility, safety and preliminary evidence of the effectiveness of a home-based exercise programme for older people with Alzheimer’s disease: A pilot randomized controlled trial. Clin Rehabil. 2013;27:427–38. doi:10.1177/0269215512460877.

59. Vreugdenhil A, Cannell J, Davies A, Razay G. A community-based exercise programme to improve functional ability in people with Alzheimer’s disease: A randomized controlled trial. Scand J Caring Sci. 2012;26:12–9. doi:10.1111/j.1471-6712.2011.00895.x.

61. Wesson J, Clemson L, Brodaty H, Lord S, Taylor M, Gitlin L, Close J. A feasibility study and pilot randomised trial of a tailored prevention program to reduce falls in older people with mild dementia. BMC Geriatr. 2013;13:89. doi:10.1186/1471-2318-13-89.

65. Burgener SC, Yang Y, Gilbert R, Marsh-Yant S. The effects of a multimodal intervention on outcomes of persons with early-stage dementia. Am. J. Alzheimers Dis. Other Dement. 2008;23:382–94. doi:10.1177/1533317508317527.

67. Kim M-J, Han C-W, Min K-Y, Cho C-Y, Lee C-W, Ogawa Y, et al. Physical Exercise with Multicomponent Cognitive Intervention for Older Adults with Alzheimer’s Disease: A 6-Month Randomized Controlled Trial. Dement Geriatr Cogn Dis Extra. 2016;6:222–32. doi:10.1159/000446508.

73. Yoon JE, Lee SM, Lim HS, Kim TH, Jeon JK, Mun MH. The effects of cognitive activity combined with active extremity exercise on balance, walking activity, memory level and quality of life of an older adult sample with dementia. J. Phys. Ther. Sci. 2013;25:1601–4. doi:10.1589/jpts.25.1601.

74. Dawson N, Judge KS, Gerhart H. Improved Functional Performance in Individuals With Dementia After a Moderate-Intensity Home-Based Exercise Program: A Randomized Controlled Trial. J Geriatr Phys Ther. 2019;42:18–27. doi:10.1519/JPT.0000000000000128.

76. Francese T, Sorrell J, Butler FR. The effects of regular exercise on muscle strength and functional abilities of late stage Alzheimer’s residents. Am J Alzheimers Dis Other Demen. 1997;12:122–7. doi:10.1177/153331759701200305.

77. Hauer K, Ullrich P, Dutzi I, Beurskens R, Kern S, Bauer J, Schwenk M. Effects of Standardized Home Training in Patients with Cognitive Impairment following Geriatric Rehabilitation: A Randomized Controlled Pilot Study. Gerontology. 2017;63:495–506. doi:10.1159/000478263.

78. Hauer K, Schwenk M, Zieschang T, Essig M, Becker C, Oster P. Physical training improves motor performance in people with dementia: A randomized controlled trial. J Am Geriatr Soc. 2012;60:8–15. doi:10.1111/j.1532-5415.2011.03778.x.

80. Santana-Sosa E, Barriopedro MI, López-Mojares LM, Pérez M, Lucia A. Exercise training is beneficial for Alzheimer’s patients. Int J Sports Med. 2008;29:845–50. doi:10.1055/s-2008-1038432.

83. Kampragkou C, Iakovidis P, Kampragkou E, Kellis E. Effects of a 12-Week Aerobic Exercise Program Combined with Music Therapy and Memory Exercises on Cognitive and Functional Ability in People with Middle Type of Alzheimer’s Disease. International Journal of Physiotherapy 2017. doi:10.15621/ijphy/2017/v4i5/159420.

84. Sobol NA, Hoffmann K, Frederiksen KS, Vogel A, Vestergaard K, Brændgaard H, et al. Effect of aerobic exercise on physical performance in patients with Alzheimer’s disease. Alzheimers Dement. 2016;12:1207–15. doi:10.1016/j.jalz.2016.05.004.

85. Toulotte C, Fabre C, Dangremont B, Lensel G, Thévenon A. Effects of physical training on the physical capacity of frail, demented patients with a history of falling: A randomised controlled trial. Age Ageing. 2003;32:67–73. doi:10.1093/ageing/32.1.67.

88. Guralnik JM, Seeman TE, Tinetti ME, Nevitt MC, Berkman LF. Validation and use of performance measures of functioning in a non-disabled older population: MacArthur studies of successful aging. Aging (Milano). 1994;6:410–9. doi:10.1007/BF03324272.

89. Rolland Y, Pillard F, Klapouszczak A, Reynish E, Thomas D, Andrieu S, et al. Exercise program for nursing home residents with Alzheimer’s disease: A 1-year randomized, controlled trial. J Am Geriatr Soc. 2007;55:158–65. doi:10.1111/j.1532-5415.2007.01035.x.

92. Kressig RW, Beauchet O. Guidelines for clinical applications of spatio-temporal gait analysis in older adults. Aging Clin Exp Res. 2006;18:174–6. doi:10.1007/BF03327437.

93. Pedrinolla A, Venturelli M, Fonte C, Munari D, Benetti MV, Rudi D, et al. Exercise Training on Locomotion in Patients with Alzheimer’s Disease: A Feasibility Study. J Alzheimers Dis. 2018;61:1599–609. doi:10.3233/JAD-170625.

94. Schwenk M, Dutzi I, Englert S, Micol W, Najafi B, Mohler J, Hauer K. An intensive exercise program improves motor performances in patients with dementia: Translational model of geriatric rehabilitation. J Alzheimers Dis. 2014;39:487–98. doi:10.3233/JAD-130470.

98. Steinberg M, Leoutsakos J-MS, Podewils LJ, Lyketsos CG. Evaluation of a home-based exercise program in the treatment of Alzheimer’s disease: The Maximizing Independence in Dementia (MIND) study. Int J Geriatr Psychiatry. 2009;24:680–5. doi:10.1002/gps.2175.

100. Werner C, Wiloth S, Lemke NC, Kronbach F, Jansen C-P, Oster P, et al. People with Dementia Can Learn Compensatory Movement Maneuvers for the Sit-to-Stand Task: A Randomized Controlled Trial. J Alzheimers Dis. 2017;60:107–20. doi:10.3233/JAD-170258.

105. Roach KE, Tappen RM, Kirk-Sanchez N, Williams CL, Loewenstein D. A randomized controlled trial of an activity specific exercise program for individuals with Alzheimer disease in long-term care settings. J Geriatr Phys Ther. 2011;34:50–6. doi:10.1519/JPT.0b013e31820aab9c.

112. Bossers WJR, van der Woude LHV, Boersma F, Hortobágyi T, Scherder EJA, van Heuvelen MJG. Comparison of Effect of Two Exercise Programs on Activities of Daily Living in Individuals with Dementia: A 9-Week Randomized, Controlled Trial. J Am Geriatr Soc. 2016;64:1258–66. doi:10.1111/jgs.14160.

124. Cott CA, Dawson P, Sidani S, Wells D. The effects of a walking/talking program on communication, ambulation, and functional status in residents with Alzheimer disease. Alzheimer Dis Assoc Disord. 2002;16:81–7. doi:10.1097/00002093-200204000-00005.

125. Pomeroy VM, Warren CM, Honeycombe C, Briggs RS, Wilkinson DG, Pickering RM, Steiner A. Mobility and dementia: Is physiotherapy treatment during respite care effective? Int J Geriatr Psychiatry. 1999;14:389–97. doi:10.1002/(SICI)1099-1166(199905)14:5<389::AID-GPS933>3.0.CO;2-8.

126. Schwenk M, Zieschang T, Oster P, Hauer K. Dual-task performances can be improved in patients with dementia: A randomized controlled trial. Neurology. 2010;74:1961–8. doi:10.1212/WNL.0b013e3181e39696.

127. Hawk C, Hyland JK, Rupert R, Colonvega M, Hall S. Assessment of balance and risk for falls in a sample of community-dwelling adults aged 65 and older. Chiropr Osteopat. 2006;14:3. doi:10.1186/1746-1340-14-3.

128. Vellas BJ, Rubenstein LZ, Ousset PJ, Faisant C, Kostek V, Nourhashemi F, et al. One-leg standing balance and functional status in a population of 512 community-living elderly persons. Aging Clin Exp Res. 1997;9:95–8. doi:10.1007/BF03340133.

129. Moe-Nilssen R. Test-retest reliability of trunk accelerometry during standing and walking. Arch Phys Med Rehabil. 1998;79:1377–85. doi:10.1016/S0003-9993(98)90231-3.

130. Clark RA, Bryant AL, Pua Y, McCrory P, Bennell K, Hunt M. Validity and reliability of the Nintendo Wii Balance Board for assessment of standing balance. Gait Posture. 2010;31:307–10. doi:10.1016/j.gaitpost.2009.11.012.

131. Mathias S, Nayak US, Isaacs B. Balance in elderly patients: the “get-up and go” test. Arch Phys Med Rehabil. 1986;67:387–9.

132. Pomeroy V. Development of an ADL Oriented Assessment-of-Mobility Scale Suitable for Use with Elderly People with Dementia. Physiotherapy. 1990;76:446–8. doi:10.1016/S0031-9406(10)62968-9.

133. Rockwood K, Rockwood MRH, Andrew MK, Mitnitski A. Reliability of the hierarchical assessment of balance and mobility in frail older adults. J Am Geriatr Soc. 2008;56:1213–7. doi:10.1111/j.1532-5415.2008.01773.x.

134. Roach KE, van Dillen LR. Development of an Acute Care Index of Functional status for patients with neurologic impairment. Phys. Ther. 1988;68:1102–8. doi:10.1093/ptj/68.7.1102.

135. Bohannon RW. Sit-to-stand test for measuring performance of lower extremity muscles. Percept Mot Skills. 1995;80:163–6. doi:10.2466/pms.1995.80.1.163.

136. Reuben DB, Siu AL. An objective measure of physical function of elderly outpatients. The Physical Performance Test. J Am Geriatr Soc. 1990;38:1105–12. doi:10.1111/j.1532-5415.1990.tb01373.x.

137. Stewart DA, Burns J, Dunn SG, Roberts MA. The two-minute walking test: A sensitive index of mobility in the rehabilitation of elderly patients. Clin Rehabil. 2016;4:273–6. doi:10.1177/026921559000400404.

138. Rolland YM, Cesari M, Miller ME, Penninx BW, Atkinson HH, Pahor M. Reliability of the 400-m usual-pace walk test as an assessment of mobility limitation in older adults. J Am Geriatr Soc. 2004;52:972–6. doi:10.1111/j.1532-5415.2004.52267.x.

139. Astrand PO, Ryhming I. A nomogram for calculation of aerobic capacity (physical fitness) from pulse rate during sub-maximal work. J Appl Physiol. 1954;7:218–21. doi:10.1152/jappl.1954.7.2.218.

140. Jones CJ, Rikli RE, Max J, Noffal G. The reliability and validity of a chair sit-and-reach test as a measure of hamstring flexibility in older adults. Res Q Exerc Sport. 1998;69:338–43. doi:10.1080/02701367.1998.10607708.

141. Rikli RE, Jones CJ. Senior fitness test manual. Champaign, IL: Human Kinetics; 2006.

142. Jebsen RH, Taylor N, Trieschmann RB, Trotter MJ, Howard LA. An objective and standardized test of hand function. Arch Phys Med Rehabil. 1969;50:311–9.

143. Lord SR, Menz HB, Tiedemann A. A physiological profile approach to falls risk assessment and prevention. Phys. Ther. 2003;83:237–52. doi:10.1093/ptj/83.3.237.
